# Genes and Gene Ontologies Common to Airflow Obstruction and Emphysema in the Lungs of Patients with COPD

**DOI:** 10.1371/journal.pone.0017442

**Published:** 2011-03-15

**Authors:** Santiyagu M. Savarimuthu Francis, Jill E. Larsen, Sandra J. Pavey, Edwina E. Duhig, Belinda E. Clarke, Rayleen V. Bowman, Nick K. Hayward, Kwun M. Fong, Ian A. Yang

**Affiliations:** 1 Department of Thoracic Medicine, The Prince Charles Hospital, Brisbane, Australia; 2 Department of Anatomical Pathology, The Prince Charles Hospital, Brisbane, Australia; 3 School of Medicine, The University of Queensland, Brisbane, Australia; 4 Queensland Institute of Medical Research, Brisbane, Australia; Johns Hopkins School of Medicine, United States of America

## Abstract

Chronic obstructive pulmonary disease (COPD) is a major public health problem with increasing prevalence worldwide. The primary aim of this study was to identify genes and gene ontologies associated with COPD severity. Gene expression profiling was performed on total RNA extracted from lung tissue of 18 former smokers with COPD. Class comparison analysis on mild (n = 9, FEV_1_ 80–110% predicted) and moderate (n = 9, FEV_1_ 50–60% predicted) COPD patients identified 46 differentially expressed genes (p<0.01), of which 14 genes were technically confirmed by quantitative real-time-PCR. Biological replication in an independent test set of 58 lung samples confirmed the altered expression of ten genes with increasing COPD severity, with eight of these genes (*NNMT*, *THBS1*, *HLA-DPB1*, *IGHD*, *ETS2*, *ELF1*, *PTGDS* and *CYRBD1*) being differentially expressed by greater than 1.8 fold between mild and moderate COPD, identifying these as candidate determinants of COPD severity. These genes belonged to ontologies potentially implicated in COPD including angiogenesis, cell migration, proliferation and apoptosis. Our secondary aim was to identify gene ontologies common to airway obstruction, indicated by impaired FEV_1_ and KCO. Using gene ontology enrichment analysis we have identified relevant biological and molecular processes including regulation of cell-matrix adhesion, leukocyte activation, cell and substrate adhesion, cell adhesion, angiogenesis, cell activation that are enriched among genes involved in airflow obstruction. Exploring the functional significance of these genes and their gene ontologies will provide clues to molecular changes involved in severity of COPD, which could be developed as targets for therapy or biomarkers for early diagnosis.

## Introduction

COPD is predicted to become the fifth most common cause of disease burden worldwide by 2020 [1]. COPD contributes a huge economic burden; estimates from Australia indicate over US $9 billion in health care costs annually. COPD is often under-diagnosed, as it is not usually recognized until its severity is moderately advanced, when preventive measures are too late to prevent severe disease [2]. Understanding the pathogenesis of COPD severity, especially in the earlier mild and moderate stages of COPD, would greatly enhance diagnostic and therapeutic strategies for this deadly disease.

Because of the complexity of COPD pathophysiology in the lungs, measuring COPD severity comprehensively is challenging. The different compartments of the tracheobronchial tree (large airways, small airways (bronchioles) and alveoli) contribute differently and specifically to the pathophysiological changes of COPD. FEV_1_ is a physiological measure of airway obstruction particularly in the small airways [3], but fails to correlate with the extent of emphysema severity in the parenchyma [4]. Gas transfer (KCO) correlates with emphysema severity (alveolar destruction in the lung parenchyma), and emphysema contributes to airflow obstruction due to loss of elastic recoil; however, KCO does not correlate strongly with FEV_1_ in COPD. Additionally, the bronchioles and parenchyma demonstrate different patterns of gene expression in COPD [5]. Clearly, pathophysiological studies of markers of COPD severity should assess spirometric indicators of airflow obstruction (reduced FEV_1_ and FEV_1_/VC), as well as impairment of gas transfer (KCO) as an indicator of emphysema.

Microarrays are useful high-throughput tools for studying the expression of multiple genes simultaneously, as markers of disease pathogenesis. Microarrays have been previously used to profile biospecimens from COPD patients to identify characteristic gene expression patterns associated with the presence of COPD, its severity and subphenotypes. Several studies have addressed gene expression in specific cell types [6], [7], [8]. Whilst studying specific cell types is important, multiple cell responses are likely to drive the severity of COPD. Only two previous studies by Wang *et al* [9] and Bhattacharya *et al* [10], have used lung tissues to profile COPD severity. The former study included limited number of patients in the more severe subgroups, with 21 GOLD stage 0, 9 GOLD stage I, 10 GOLD stage II and only 3 GOLD stage III patients. The gene signature was correlated to forced expiratory flow between 25% and 75% of forced expiratory volume (FEF_25–75%_). The latter study profiles 56 lung tissues, but compared normal cases to COPD patients with any degree of severity (GOLD 0-IV). Similarly, profiles linked to emphysema has been reported by Spira *et al* [11], Golpon *et al* [12] and Savarimuthu *et al* [13].

We primarily sought to identify disease-associated genes and gene ontologies related to airflow obstruction. To better understand the pathobiology of disease severity in COPD, we searched for genes differentially expressed in lung tissue using microarrays between mild (GOLD Stage I) and moderate (GOLD Stage II) COPD patients, stratifying them by FEV_1_. These genes could provide clues to molecular changes involved in the severity of COPD that could potentially be developed as targets for therapy or biomarkers for early diagnosis. Secondarily, we sought to identify gene ontologies common to both airflow obstruction, indicated by reduced FEV_1_, and emphysema, indicated by KCO. In a complex disease such as COPD, it is probable that groups of genes rather than single genes are involved in disease development and severity. To further explore common ontologies and pathways, differentially expressed genes were collated with those previously identified as associated with COPD severity [4]–[9].

## Methods

### Sample selection, sample processing and microarray experiments

Patients with varying severity of COPD undergoing curative resection for lung cancer at The Prince Charles Hospital, Brisbane, provided written informed consent for the collection and study of samples of macroscopically normal non-tumour lung. The study was approved by the institutional ethics committees of The Prince Charles Hospital (TPCH) and The University of Queensland.

Inclusion and exclusion criteria for the COPD patients were as previously published by our laboratory [13]. Nine patients with mild COPD who had post-bronchodilator FEV_1_/VC<0.7 and FEV_1_>80% predicted (GOLD Stage I) were compared with nine patients with moderate COPD who had post-bronchodilator FEV_1_/VC<0.7 and FEV_1_ measurements between 50 and 60% predicted (within GOLD Stage II). To ensure a distinct separation of mild and moderate COPD severity, patients with FEV_1_ between 60 and 80% predicted were not included in the training set, in order to polarize the comparison groups. Spirometry was performed as described previously [15] and conformed to standard guidelines [16], [17].

Sample processing and microarray experiments were as previously described [13], [14]. Briefly, twenty nanograms of total RNA was isolated from lung tissue, cleaned, quality evaluated, and quantified using TRIzol (Invitrogen Corporation, Carlsbad, CA, USA), Qiagen RNeasy mini kit (Qiagen, Hilden, Germany) and Bioanalyzer (Agilent Technologies Inc., Santa Clara, CA, USA) respectively. The study design for microarray experiments conformed to MIAME guidelines guidelines (http://www.mged.org/Workgroups/MIAME/miame_checklist.html). Test lung RNA and commercial universal reference (RNA Stratagene, La Jolla, CA, USA) was reverse transcribed to cDNA along with. Lung cDNA and reference cDNA were labeled with Cy5 and Cy3 dyes respectively (Amersham/GE Healthcare, Buckinghamshire, England) and then co-hybridized on an Operon V2.0 12k element microarray chip (http://www.operon.com). The slides were washed and scanned to obtain raw images which were combined and filtered in Imagene V5.1 (BioDiscovery, Inc., El Segundo, CA, USA). The samples were normalized using Lowess and probes with missing values in 50% or more samples were removed from the analysis in Avadis V4.3 (Strand Genomics, Bangalore, India). All normalized data has been deposited in the NCBI Gene Expression Omnibus (GEO) public repository (http://www.ncbi.nlm.nih.gov/geo) and can be accessed through the accession number GSE17770. Log ratio variation gene filtration was performed in BRB-Array Tools V3.8 (developed by Dr Richard Simon and Amy Peng Lam, freely accessible online at http://linus.nci.nih.gov/BRB-ArrayTools.html), to only include probes that varied significantly (*P*<1.0E-6) from the median of all samples.

### Data analysis

#### Gene discovery and validation

Class comparison analysis using an independent *t*-test was performed in BRB-ArrayTools to find genes differentially expressed between mild and moderate COPD patients at p<0.01. Genes identified were technically validated using qRT-PCR with primers designed to amplify the same transcripts represented on the microarray platform. Technical variability in amount of mRNA per sample was normalized to the universally expressed housekeeping genes, 18s rRNA, *HGS* and *ACTN4* [18]. The average gene expression for cases with moderate COPD (GOLD Stage II) divided by the arithmetic average of expression in cases with mild COPD (GOLD Stage I) was calculated as the fold change. Genes that displayed concordant increase or decrease in fold change as measured by both microarray and qRT-PCR were determined to be technically validated.

Genes that were technically validated were selected for biological validation in an independent set of 58 lung samples from the TPCH tissue bank; these samples were selected on the basis of the patients' FEV_1_ and FVC measurements. No exclusions for smoking status, cumulative pack years of smoking, histology, steroid use, or comorbidity were applied to the test set, aiming for wide inclusion in the test set to confirm generalizability. Total RNA from the non-tumour lung samples was extracted using TRIzol and reverse transcribed to cDNA using Superscript III (Invitrogen). Genes were quantitated using qRT-PCR and normalized to housekeepers as described above. Quantitative expression of each gene was calculated using Pfaffl's method [19]. The change in fold expression was calculated between patients with mild and moderate COPD in the test set. Genes with concordant fold change in the test and training set and greater than 1.8 fold differences in the test set were determined to be biologically validated.

#### Biological validation of candidate genes in published datasets

External validation in public gene expression microarray datasets was performed to ensure that differentially expressed genes were authentic candidates, which were not overfitted to a particular cohort. The Bhattacharya *et al* [10] and Wang *et al* [9] datasets were downloaded from GEO Omnibus (GSE8581 and GSE8500, repectively using array platforms, Affymetrix Human U133Plus 2.0 and Rosetta hu25k respectively). Chip comparer (http://tenero.duhs.duke.edu/genearray/perl/chip/chipcomparer.pl) was used to find probes represented on Affymetrix (46,000 probes) or Rosetta (21,000 probes) and the Operon V2.0 platform. Normalized signals were imported into BRB-Array Tools and only signals that were called present were used in the analysis. Leave one out cross validation (LOOCV) analysis was performed to test the prediction accuracy of our candidate genes in other datasets.

#### Gene Enrichment and Pathway analysis

GOEast (Gene Ontology Enrichment Analysis Software Toolkit) was used to identify gene ontologies enriched significantly (*p*<0.05) between the various studies [20]. The ontologies of gene candidates reported by previous studies, Ning [6], Wang [9], Bhattacharya [10] and this study (henceforth refered to as TPCH-FEV_1_) were individually analysed and overlayed to find commonality between their gene ontologies. Additionally, we used the above method to identify gene ontologies common to severity of COPD (airflow obstruction) and emphysema, indicated by impaired FEV_1_ (TPCH-FEV1, and KCO, respectively. Ingenuity Pathway Analysis (IPA) was performed to find direct relationships between candidate genes.

## Results

### Demographics

Characteristics of patients included in the test and independent set are listed in Table 1. The training samples were classified according to GOLD classification into mild COPD (Stage I) (n = 9, median FEV_1_ 94.7% predicted, range 80–110% predicted) and moderate COPD (Stage II) (n = 9, median FEV_1_ 52.6% predicted, range 50–60% predicted). All subjects included in the study were Caucasians. The training set consisted of former smokers, with greater than 20 pack year smoking history who had quit at least 10 months prior to surgery. The subjects did not have evidence of obstructive pneumonitis due to tumor obstruction and were not taking any inhaled or oral steroids. The test set samples consisted of patients with mild COPD (n = 39, median FEV_1_ 91% predicted, range 80–135% predicted) and moderate COPD (n = 19, median FEV_1_ 54% predicted, range 30–60% predicted). The test group included current and former smokers with a 1 to 224 pack year history.

**Table 1:** Demographics of COPD patients in the TPCH training and test sets.

	Training Set		Test Set	
	GOLD Stage I	GOLD Stage II	GOLD Stage I	GOLD Stage II
n	9	9	39	19
AgeGender	69±8M: 7F: 2	70±6M: 7F: 2	65±9M: 29F: 10	65±10M: 15F: 4
FEV_1_% predicted	94±7	54±3	94±11	52±8
FEV_1_/VC	0.60±0.03	0.50±0.05	0.60±0.15	0.52±0.08
Pack years#	66 [24–135]	78 [40–158]	Current: 60Former: 39	Current: 55Former: 53
Smoking Status	Former	Former	16 Current23 Former	3 Current16 Former
Site of Lobectomy*	1-LLL2-LUL1-RL1-RLL4-RUL	5-RLL1-RML3-RUL	5-LLL7-LUL5-RLL2-RML16-RUL4-LL	5-LLL6-LUL1-RLL2-RML4-RUL1-LL

*Values are shown in mean ± SD where indicated except ^#^ where values are shown in Median [Min-Max].*

*LL- Left Lung, LLL- Left Lower Lobe, LUL- Left Upper Lobe, RL -Right Lung, RLL- Right Lower Lobe, RUL- Right Upper Lobe, RML- Right Middle Lobe. FEV_1_- forced expired volume in one second, VC- vital capacity (ventilatory measurement).

### Gene discovery and Validation

After initial filtering for poor quality spots and normalization, 20,274 probes representing 12,178 known genes remained. After filtering, 2,155 genes were identified as having significant variation in expression (p<1.0E-6) compared to the average expression of per gene in all samples. Unsupervised hierarchical clustering of the filtered genelist revealed no obvious association of the genes with the age, gender or smoking history of the samples. A *t*-test identified 46 probes representing 46 genes significantly different in expression (p<0.01) between lungs from patients with mild and moderate COPD (Table S1). These 46 genes were 67% accurate in classifying mild (GOLD stage I) and moderate (GOLD stage II) COPD using LOOCV with diagonal linear discriminate algorithm in the training samples. Sixteen of the 46 genes had incomplete sequence complementarity to a corresponding gene using BLAST searches in Ensembl genome browser based on the human build 17 and were excluded from further analysis, leaving 30 probes for technical validation. Fourteen genes demonstrated consistent changes in gene expression (concordant direction of fold-change) by qRT-PCR when comparing mild and moderate COPD samples. The reduced gene list was 94% accurate (90% sensitive, 100% specific) in classifying the COPD severity groups in the training samples.

**Figure 1:**
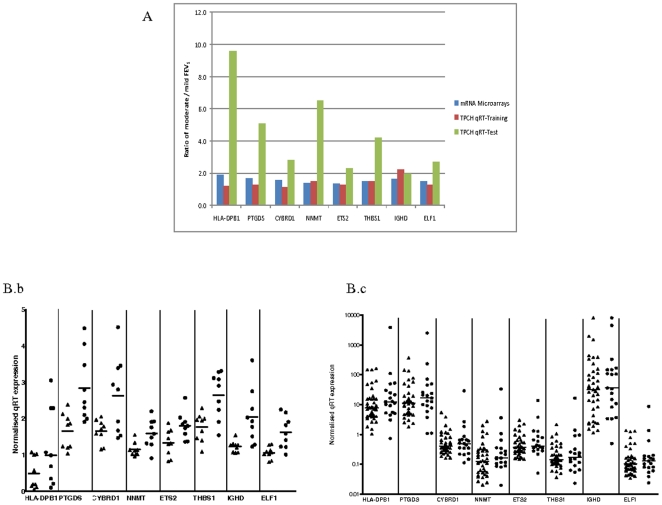
Technical Validation of eight candidate genes in the training and test set using qRT-PCR. **A**) Comparison of mRNA expression differences between mild (GOLD I) and moderate (GOLD II) COPD patients in the candidate eight genes measured by qRT-PCR and gene expression microarrays. **B**) Graph of candidate genes in TPCH training set (**b**) (n = 30) and TPCH test set (**c**) (n = 63). The figures 1b&c show the median gene expression of the mild (▴) and moderate (•) emphysema.

Biological validation of the 14 candidate genes was performed in an independent test set of 58 COPD patients selected from the TPCH tissue bank. The difference in average gene expression between mild and moderate COPD patients (based on FEV_1_) was calculated. Genes with concordant changes in direction with the microarray data and the training set were identified as candidates. The magnitude of fold difference in expression between COPD groups, based on FEV_1_, was greater than 1.8 for eight of these genes (*NNMT*, *THBS1*, *HLA-DPB1*, *IGHD*, *ETS2*, *ELF1*, *PTGDS* and *CYRBD1*) (Figure 1A and 1B (b&c)). These genes were therefore considered candidates for a functional role in COPD severity.

### Biological validation of candidate genes in Bhattacharya and Wang datasets

The ability of these eight genes to predict COPD severity based on FEV_1_ in two public datasets was tested using class prediction in BRB-ArrayTools V3.8. All eight genes were represented in the Bhattacharya *et al* dataset that used an Affymetrix platform and seven were represented in the Wang *et al* dataset that used a Rosetta platform. The candidate genes were 55% accurate (61% specificity and 46% sensitivity) and 62% accurate (63% specificity and 57% sensitivity) in classifying the Bhattacharya *et al* and Wang *et al* datasets respectively. Seven of the eight genes demonstrated a concordant change in expression between mild and moderate COPD patients in at least one of the two public datatsets. Additonally, the change in direction of three genes (*THBS1*, *NNMT* and *HLA-DPB1*) were consistent in all three studies (Figure S1).

#### Gene Enrichment and Pathway analysis

Gene Enrichment analysis and pathway analysis were performed to identify gene ontologies and functions that are commonly dysregulated in relation to COPD in published gene expression datasets. A summary of the gene ontologies enriched in TPCH-FEV_1_ study as well as the published studies where FEV_1_ or KCO (or DLCO) was used to stratify COPD patients is shown in Table S2 and Figures S2a and S2b. Several ontologies were found to be commonly enriched in gene profiles identified by at least two of the four COPD studies that used only FEV_1_ to classify COPD severity (Ning, Wang, Bhattacharya and this study), namely, chronic inflammatory response, negative regulation of fibrinolysis, regulation of fibroblast growth factor receptor signaling pathway, sprouting angiogenesis, regulation of macrophage activation, positive and negative regulation of blood vessel endothelial cell migration and regulation of protein processing.

Similarly, common ontologies were found to be enriched in gene profiles of FEV_1_ or KCO (or DLCO), namely, regulation of focal adhesion formation, regulation of oxygen and reactive oxygen species metabolic process, negative regulation of focal adhesion formation, prostaglandin biosynthetic process, positive regulation of leukocyte chemotaxis, and apoptotic cell clearance. Gene ontologies enriched in each of the published studies, Ning [6], Wang [9], Bhattacharya [10], Spira [11], Golpon [12] and our previous study (TPCH-KCO), Savarimuthu [13], as well as the TPCH-FEV_1_ study, are listed in Tables S3, S4, S5, S6, S7, S8 and S9.

**Figure 2:**
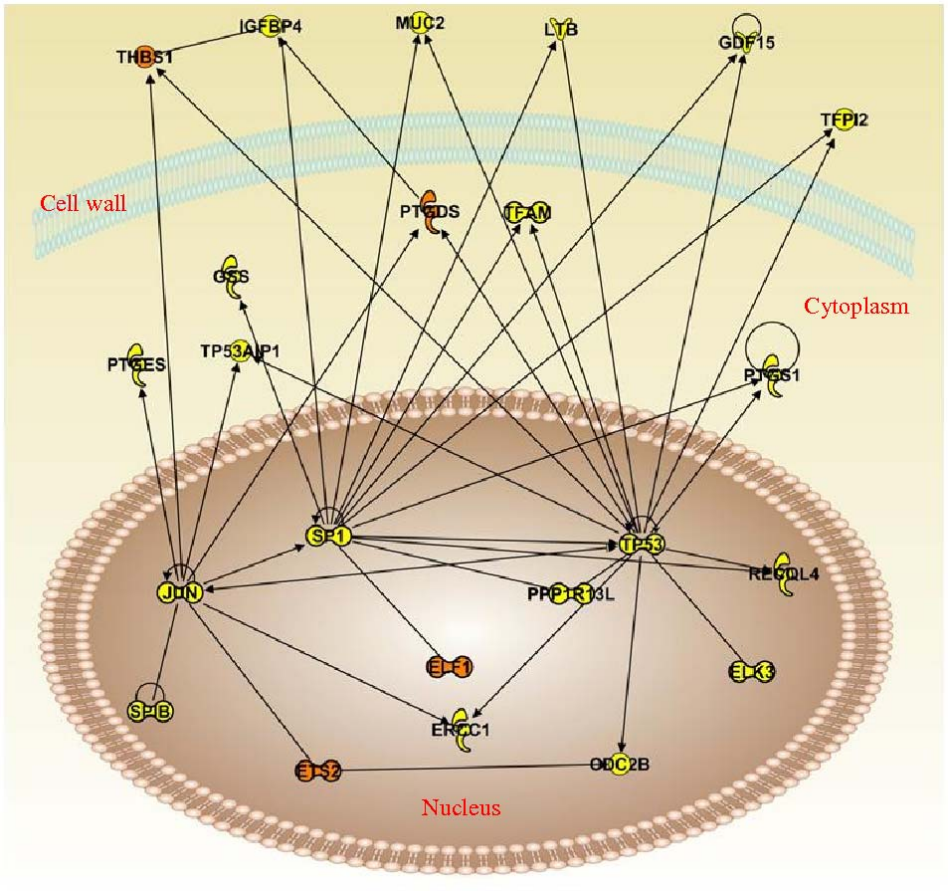
Ingenuity Pathway Analysis (IPA) on the eight biologically validated candidate genes. *Bold lines* indicates direct link, *dotted lines* indicate indirect link. Orange nodes indicate input genes into the pathway analysis and the different symbols indicate gene functions.

Pathway analysis using IPA showed direct associations between gene candidates (*THBS1*, *PTGDS*, *ELF1* and *ETS2*) and genes involved in cell cycle regulation (tumour protein 53, (*TP53*) and *JUN*), apoptosis (insulin-like growth factor binding protein, *IGFBP4*), transcription (*SP1*), mucin production (*MUC2*) and DNA repair (excision repair cross complement, *ERCC1*) (Figure 2).

## Discussion

COPD is a complex chronic lung disease characterised by irreversible airflow limitation in the lung, as clinically diagnosed by spirometric measurement using FEV_1_, and by lung parenchymal destruction, as clinically diagnosed by gas transfer measurement using KCO, in addition to radiological imaging. We previously reported on seven candidate genes involved in emphysema severity, diagnosed by reduced KCO [13], which were capable of predicting independent test sets with high accuracy [13], [14]. Here, we have investigated the lung tissue of COPD patients, and stratified patients by FEV_1_. We have identified 46 genes involved in COPD severity; these genes were differentially expressed between mild and moderate COPD (GOLD class I and II severities). Technical and biological validation found eight genes with reproducible differences in mRNA expression between the mild and moderate COPD groups based on their FEV_1_. These genes are therefore potentially associated with COPD severity.

Several studies have used expression profiling to characterise genes involved in COPD of any phenotype (especially emphysema) [6], [7], [8], [9], [10], yet only two have specifically explored the gene expression in lung tissues of COPD patients stratified by FEV_1_ [9], [10]. The first of the studies [9] identified a 203 transcipt gene panel associated with the various stages of COPD (GOLD 0-III); however the severe stage comprised of only three cases. The second study [10] identified a set of 65 transcripts that were differentially expressed when comparing normal and severe COPD (GOLD IV) patients. These transcripts were able to predict an independent dataset published by Spira *et al* [11] with 97% accuracy. We identified eight genes capable of classifying mild (GOLD I) and moderate (GOLD II) COPD patients in the TPCH-training set, TPCH-test set and in external validation datasets [9], [10].

A strength of our study is validation of results using a number of methods: technical validation (validation of technique) through replication of the microarray gene expression findings using qPCR on the same samples of the training set; biological validation in an independent test set of samples from the same tissue bank (from our hospital); biological validation in independent external training set from other datasets (publicly available from other centres). Applying these methods we identified candidate genes that are involved in molecular, biological or cellular functions that are potentially associated with COPD pathogenesis. Gene names and their function are summarized in Table 2. *NNMT* has been previously described to be involved in COPD [21], [22]. NNMT is overexpressed in quadriceps muscles of COPD patients and likely to contribute to cell proliferation and migration. The expression of *NNMT* is significantly triggered by IL6, TNFα and TGFβ [21]. We found expression of NNMT to inversely correlate with FEV_1_, a result which is similar to previously published studies. *THBS1* activates TGFβ and matrix metalloproteinases (MMP) [9], mediates cell-cell and cell-matrix interactions, and is involved in angiogenesis, proliferation and platelet aggregation. *THBS1* has been previously identified in COPD lung and its expression validated by Wang *et al* [11] using immunohistochemistry. These mechanisms are key indicators of inflammation and hypoxia in COPD patients. *CYBRD1* is as an iron (Fe+) reductase in the airway epithelial cells, an important mechanism inducing hyperoxia and oxidative stress in the lung [23]. *PTGDS* [24], *IGHD* [25] and *HLA-DPB1* regulate host immunity and inflammation. Spira *et al* found *PTGDS* expression to negatively correlate (*r* = 0.63) with FEV_1_ post-bronchodilator (% predicted) [11]. Two of the candidate genes from this study, *ETS2* and *ELF1*, are transcription factors. ETS2 regulates genes involved in stem cell development, cell senescence and death and tumourigenesis. ETS2 is related to important COPD pathways through molecules such as HRAS, HDAC2, EGFR and RAF1. ELF1 interacts with known COPD genes, NFκB1, NFYB, RB1, and SP1. ELF1 acts both as an enhancer (increases cytokine production), and repressor (negative regulator of T-cell receptor mediated signaling pathway). Therefore, integration of gene ontology and recent literature shows that the candidate genes identified in our study are involved in processes that are potentially associated with COPD pathogenesis.

**Table 2:** List of the eight candidate genes and their function.

Gene Symbol	Gene description	Function
*NNMT*	Nicotinamide N-methyltransferase	Methylating agent, regulating gene expression
*THBS1*	Thrombospondin 1	Cell-cell and cell-matrix interactions/angiogenesis, tumorigenesis and platelet aggregation
*IGHD*	Immunoglobulin heavy delta chain	Apoptosis/survival of B-cell chronic lymphocytic leukemia
*HLA-DPB1*	major histocompatability complex, class II, DP Beta 1	Antigen presenting cells that regulate the immune system
*PTGDS*	Prostaglandin D2 synthase	Positive regulator of apoptosis
*CYBRD1*	Cytochrome B-Reductase 1	Hyperoxia/oxidative stress
*ETS2*	Erythroblastosis virus E26 oncogene homologue 2	Stem cell development, cell senescence and death and tumorigenesis
*ELF1*	E74 like factor 1	Cytokine production/negative regulator of T-cell receptor mediated signaling pathway

Results of the TPCH-FEV_1_ study were compared with previously published gene expression profiling studies of COPD (Ning *et al* [6], Wang *et al* [9] and Bhattacharya *et al* [10]) to determine if the same candidate genes were discovered by others. These studies reported 327, 203 and 84 respective candidates to be involved in COPD severity. Three of the eight candidates we identified were reported by at least one of the three previous studies: *THBS1* (Wang *et al*), *IGHD1* (Bhattacharya *et al*) and *CYBRD1* (Ning *et al*) and they were similarly up-regulated in moderate/severe COPD, as observed in this study. Although there was not much overlap between individual genes, there was a significant enrichment of common gene ontologies [26], [27]. This strengthens the association of our candidates with FEV_1_ predicted COPD severity. Additionally, class prediction (LOOCV) analysis provided an unbiased estimate of the classification performance of the eight genes in independant samples with a classification accuracy of 55% in Bhattacharya *et al* and 62% in Wang *et al*. This shows that the genes are not over fitted to one study and therefore, they pass a critical validation step by predicting independent test samples that are biologically related with improved accuracy.

The eight candidate genes were significantly enriched for several gene ontologies (Table S3) including T-cell receptor signaling, extracellular matrix binding, positive regulation of phosphorylation cell-substrate adhesion cell adhesion and activation of MAPK activity. In order to identify gene ontologies consistently enriched in published expression profiling studies of COPD severity we compared the current study with three other studies that classifies COPD severity by FEV_1_ [6], [9], [10]. Negative regulation of cell-substrate adhesion, endothelial cell migration & proliferation, focal adhesion formation, angiogenesis, blood vessel and vasculature development, blood vessel morphogenesis, regulation of blood vessel, response to oxygen levels, and regulation of protein processing were common to atleast three studies (Ning *et al* and Wang *et al* and this study) with none enriched in all four studies. Only two ontologies, prostaglandin metabolic process and prostanoid metabolic process were common with the Bhattacharya *et al* gene set.

In comparing the shortlisted 46 candidate gene lists associated with distinguishing mild from moderate COPD classified by FEV_1_ (TPCH-FEV_1_) to the study where seven genes associated with distinguishing mild from moderate emphysema classified by KCO (TPCH-KCO) [13], only one gene, *COL6A3* (collagen, type VI, alpha 3) was common to both phenotypes. Although *COL6A3* was strongly associated with reduced KCO, it failed to validate biologically in the independent samples when classified by FEV_1_. Minimum overlap in genes may indicate the pathogenesis regulating FEV_1_ and KCO impairment involves different molecular mechanisms. Interestingly, the study by Bhattacharya and colleagues [10] identified 220 genes, of which a subset of 40 transcripts could distinguish lung tissues with mild or no emphysema from severe emphysema in a previously published gene expression study [11]. So although there is no commonality on a gene by gene basis which is not uncommon in microarray and replication studies [14], [26], [28], similar molecular mechanisms and gene ontologies are enriched between the phenotypes of impaired FEV_1_ and KCO (Figure S2b).

The lack of histological characterisation of the tissues arrayed could impact on the levels of expression due to the heterogeneity of the cells between tissues. However a recent study showed that differences in tissue content did not alter the relationship between lung function and expression [9]. Spirometric measurements of FEV1 rather than histology have been used previously to classify severity in COPD patients. In the international clinical guidelines, lung function parameters are used to diagnose the presence of COPD. FEV1% predicted is currently the major physiological indicator used clinically, in the presence of FEV1/FVC<0.70, to classify COPD disease severity, using the GOLD severity stages. Hence we used FEV1% predicted as the clinical physiological indicator and relevant phenotype to identify genes differentially expressed between mild and moderate COPD severities. False discovery rate (FDR) threshold was not specified to this discovery set. However, we used additional validation techniques (technical and biological) to validate candidate genes from the initial training set, to add robustness to the prioritisation of these genes.

In summary, eight robust candidate genes have been identified and validated, based on mRNA expression in lung tissue, that are potentially involved in COPD severity. These genes deserve further investigation to better understand their mechanistic association with COPD and explore their role as potential therapeutic targets or diagnostic markers. Future directions include testing gene function in *in vitro* models and analyzing portals of gene regulation that influence COPD severity in smokers e.g. mutation and microRNA regulation.

## Supporting Information

Figure S1 Histogram comparison of mRNA gene expression for the eight candidate genes in TPCH and public datasets.
(DOCX)

Figure S2 Gene ontologies enriched in TPCH and public datasets.
(DOCX)

Table S1 List of 46 genes associated with COPD severity identified by class comparison analysis.
(DOCX)

Table S2 Gene ontologies common to this study and previously published studies obtained using GOEAST analysis on the relevant datasets downloaded from GEO.
(DOCX)

Table S3 Gene ontologies enriched in TPCH-FEV_1_ dataset.
(DOCX)

Table S4 Gene ontologies enriched in Ning *et al* FEV_1_ dataset.
(DOCX)

Table S5 Gene ontologies enriched in Wang *et al* FEF_25–75%_ dataset.
(DOCX)

Table S6 Gene ontologies enriched in Bhattacharya *et al* FEV_1_ dataset.
(DOCX)

Table S7 Gene ontologies enriched in Spira *et al* DLCO dataset.
(DOCX)

Table S8 Gene ontologies enriched in Golpon *et al* DLCO dataset.
(DOCX)

Table S9 Gene ontologies enriched in Savarimuthu *et al* TPCH-KCO dataset.
(DOCX)
