# Supplementary material for: Genes and Gene Ontologies Common to Airflow Obstruction and Emphysema in the Lungs of Patients with COPD
Source: PLoS One. 2011 Mar 15;6(3):e17442. doi: 10.1371/journal.pone.0017442 (PMC3057973; doi:10.1371/journal.pone.0017442)
Supplement: Figure S1 — Histogram comparison of mRNA gene expression for the eight candidate genes in TPCH and public datasets. (DOCX) [file pone.0017442.s001.docx]

**Figure S1:** Histogram comparison of mRNA gene expression for the eight candidate genes between normal/mild and moderate/severe groups in all COPD studies including Ning *et al*, Wang *et al*, TPCH test and TPCH training. 4/8 genes had concordant change in gene expression between TPCH and Bhattacharya *et al* dataset. 6/7 (only 7 probes were present in the filtered dataset) genes were concordant between TPCH and Wang *et al* dataset.

**Comparison of expression of candidate genes in COPD studies using lung tissues**
