# Supplementary material for: Genes and Gene Ontologies Common to Airflow Obstruction and Emphysema in the Lungs of Patients with COPD
Source: PLoS One. 2011 Mar 15;6(3):e17442. doi: 10.1371/journal.pone.0017442 (PMC3057973; doi:10.1371/journal.pone.0017442)
Supplement: Figure S2 — Gene ontologies enriched in TPCH and public datasets. (DOCX) [file pone.0017442.s002.docx]

**Figure S2:** Using Gene Ontology Enrichment Analysis Software Toolkit (GOEast) we identified common ontologies that were enriched in a) Ning (4), Wang (6) and TPCH-FEV_1_. b) TPCH-KCO and FEV_1_ comparison.

**Gene Ontologies common to TPCH study and published studies**

**Gene ontologies common to genes differentially expressed between mild and moderate FEV_1_ and KCO**
