# Supplementary material for: Genes and Gene Ontologies Common to Airflow Obstruction and Emphysema in the Lungs of Patients with COPD
Source: PLoS One. 2011 Mar 15;6(3):e17442. doi: 10.1371/journal.pone.0017442 (PMC3057973; doi:10.1371/journal.pone.0017442)
Supplement: Table S1 — List of 46 genes associated with COPD severity identified by class comparison analysis. (DOCX) [file pone.0017442.s003.docx]

**Table S1: List of 46 genes associated with COPD severity identified by class comparison analysis**

| **Operon ID** | **Gene Symbol** | **GB Accession** | **Array Fold Change (FC)** | **TPCH Training set FC** | **TPCH Test set FC** |
| --- | --- | --- | --- | --- | --- |
| H200000212 | HLA-DPB1 | BC013184,M83664 | 1.8 | 1.2 | 9.7 |
| H200006311 | NNMT | U08021 | 1.1 | 1.5 | 6.5 |
| H200001631 | PTGDS | BM805807 | 1.7 | 1.3 | 5.1 |
| H200010238 | THBS1 | X14787 | 1.5 | 1.5 | 4.2 |
| H200004077 | CYBRD1 | AL136693 | 1.5 | 1.1 | 2.8 |
| H200013965 | ELF1 | M82882,BC010575 | 1.5 | 1.3 | 2.7 |
| H200007140 | ETS2 | AK096841 | 1.4 | 1.3 | 2.3 |
| H200021117 | DTC_HUMAN | AK090461,AK057614 | 1.6 | 2.2 | 1.9 |
| H200001049 | SPON1 | AK074803,AB018305 | 1.1 | 1.5 | 1.3 |
| H200000156 |  | M17017 | 1.7 | 2.5 | 1.3 |
| H200006810 | COL6A3 | X52022 | 1.5 | 1.38 | 0.8 |
| H200007193 | MCL1 | BC017197 | 1.9 | 1.7 | 0.7 |
| H200008450 | SCARB1 | Z22555 | 0.7 | 0.5 | 0.7 |
| H200000491 | PRG1 | X17042,CD359027 | 1.9 | 1.3 | 0.4 |
| H200002714 |  | AF143888 | 2.1 |  |  |
| H200020129 |  |  | 1.7 |  |  |
| H200008231 |  | AK025669 | 1.3 |  |  |
| H200001630 | TGM2 | M55153 | 1.5 |  |  |
| H200020839 |  | AK055796 | 0.7 |  |  |
| H200014192 |  | AK057336 | 1.09 |  |  |
| H200016177 |  |  | 2.11 |  |  |
| H200019031 |  | AK022008 | 0.8 |  |  |
| H200015768 |  | AL110135 | 1.7 |  |  |
| H200018399 |  | AF113689 | 1.4 |  |  |
| H200019399 |  | AK023658 | 1.4 |  |  |
| H200016878 |  |  | 2.2 |  |  |
| H200015117 |  |  | 1.4 |  |  |
| H200001565 | DRR1_HUMAN | AF089854 | 1.4 |  |  |
| H200013139 |  | BC032686 | 0.7 |  |  |
| H200019805 |  | X57812 | 1.6 |  |  |
| H200016619 | ACSL3 | AK001471 | 1.01 | -1.1 |  |
| H200010511 | PFN2 | AL096719,BC043646 | 0.7 | -1.0 |  |
| H200014576 | CND3_HUMAN | AF331796 | 1.0 | -1.0 |  |
| H200009212 | TRIT1 | AK000068,AF074918 | 0.7 | -1.0 |  |
| H200018911 | C13orf23 | AB107354 | 1.5 | 0.8 |  |
| H200009426 | MYLK | CR749364,AK025953 | 1.6 | 0.8 |  |
| H200000794 | NP_060563 | AK001301,CR625671 | 0.8 | 0.8 |  |
| H200011765 | HSPB8 | AF191017 | 1.7 | 0.9 |  |
| H200002457 | Q96NL3 | BC044615,AK055225 | 0.7 | 0.9 |  |
| H200002112 | KDR | AF035121 | 1.4 | 0.9 |  |
| H200016523 | NP_113664 | AL136861 | 2.1 | 0.9 |  |
| H200009332 |  | BC032311 | 1.5 | 0.9 |  |
| H200011042 | S100A8 | X06234 | 1.7 | 0.9 |  |
| H200005649 | PER1 | AF022991 | 1.3 | 1.0 |  |
| H200017981 | CCR1 | CR609171 | 0.8 | 1.03 |  |
| H200009225 | SECISBP2 |  | 0.9 | 1.3 |  |
