# Supplementary material for: Genes and Gene Ontologies Common to Airflow Obstruction and Emphysema in the Lungs of Patients with COPD
Source: PLoS One. 2011 Mar 15;6(3):e17442. doi: 10.1371/journal.pone.0017442 (PMC3057973; doi:10.1371/journal.pone.0017442)
Supplement: Table S2 — Gene ontologies common to this study and previously published studies obtained using GOEAST analysis on the relevant datasets downloaded from GEO. (DOCX) [file pone.0017442.s004.docx]

**Table S2: Gene ontologies common to TPCH FEV_1_ study and previously published COPD studies by Ning *et al*, Wang *et al* and Bhattacharya *et al* and KCO studies by Spira *et al*, Golpon *et al* and Savarimuthu *et al*, using GOEAST analysis on the relevant datasets downloaded from GEO.**

| **TPCH study, FEV_1_%predicted** | | | | **FEV_1_ % predicted** | | | **KCO/DLCO % predicted** | | |
| --- | --- | --- | --- | --- | --- | --- | --- | --- | --- |
| **GOID** | **Ontology** | **Term** | **p** | **Ning (4)** | **Wang (6)** | **Bhattacharya (7)** | **Spira (8)** | **Golpon (9)** | **Savarimuthu (3)** |
| GO:0050795 | biological_process | regulation of behavior | 0.00015668 |  |  |  | X |  |  |
| GO:0002683 | biological_process | negative regulation of immune system process | 0.00049555 |  |  |  |  |  | X |
| GO:0022410 | biological_process | circadian sleep/wake cycle process | 0.00065185 |  |  |  | X |  |  |
| GO:0042749 | biological_process | regulation of circadian sleep/wake cycle | 0.00065185 |  |  |  | X |  |  |
| GO:0045187 | biological_process | regulation of circadian sleep/wake cycle, sleep | 0.00065185 |  |  |  | X |  |  |
| GO:0042745 | biological_process | circadian sleep/wake cycle | 0.00072742 |  |  |  | X |  |  |
| GO:0002544 | biological_process | chronic inflammatory response | 0.00019388 |  | X |  |  |  |  |
| GO:0030194 | biological_process | positive regulation of blood coagulation | 0.0002638 |  | X |  |  |  |  |
| GO:0051918 | biological_process | negative regulation of fibrinolysis | 0.0002638 |  | X |  |  |  |  |
| GO:0032026 | biological_process | response to magnesium ion | 0.00033791 |  | X |  |  |  |  |
| GO:0032891 | biological_process | negative regulation of organic acid transport | 0.00033791 |  | X |  |  |  |  |
| GO:0040036 | biological_process | regulation of fibroblast growth factor receptor signaling pathway | 0.00033791 |  | X |  |  |  |  |
| GO:0002040 | biological_process | sprouting angiogenesis | 0.00049555 |  | X |  |  |  |  |
| GO:0050820 | biological_process | positive regulation of coagulation | 0.00049555 |  | X |  |  |  |  |
| GO:0051917 | biological_process | regulation of fibrinolysis | 0.00049555 |  | X |  |  |  |  |
| GO:0043030 | biological_process | regulation of macrophage activation | 0.00065185 |  | X |  |  |  |  |
| GO:0043536 | biological_process | positive regulation of blood vessel endothelial cell migration | 0.00065185 |  | X |  |  |  |  |
| GO:0010953 | biological_process | regulation of protein maturation by peptide bond cleavage | 0.0002638 | X | X |  |  |  |  |
| GO:0070613 | biological_process | regulation of protein processing | 0.0002638 | X | X |  |  |  |  |
| GO:0043537 | biological_process | negative regulation of blood vessel endothelial cell migration | 0.00049555 | X | X |  |  |  |  |
| GO:0051893 | biological_process | regulation of focal adhesion formation | 0.00043564 |  | X |  |  | X |  |
| GO:0002690 | biological_process | positive regulation of leukocyte chemotaxis | 0.00065185 |  | X |  |  | X |  |
| GO:0080010 | biological_process | regulation of oxygen and reactive oxygen species metabolic process | 0.00065185 |  | X |  |  | X |  |
| GO:0002688 | biological_process | regulation of leukocyte chemotaxis | 0.00072742 |  | X |  |  | X |  |
| GO:0051895 | biological_process | negative regulation of focal adhesion formation | 0.00019388 | X | X |  |  | X |  |
| GO:0001953 | biological_process | negative regulation of cell-matrix adhesion | 0.00049555 |  | X |  |  | X | X |
| GO:0010812 | biological_process | negative regulation of cell-substrate adhesion | 0.00060113 | X | X |  |  | X | X |
| GO:0043277 | biological_process | apoptotic cell clearance | 0.0002638 |  | X |  | X |  |  |
| GO:0006911 | biological_process | phagocytosis, engulfment | 0.00049555 |  | X |  | X |  |  |
| GO:0001516 | biological_process | prostaglandin biosynthetic process | 0.00072742 |  | X |  | X |  |  |
| GO:0046457 | biological_process | prostanoid biosynthetic process | 0.00072742 |  | X |  | X |  |  |
| GO:0030511 | biological_process | positive regulation of transforming growth factor beta receptor signaling pathway | 0.0008212 |  | X |  |  |  |  |
| GO:0032890 | biological_process | regulation of organic acid transport | 0.0008212 |  | X |  |  |  |  |
| GO:0034605 | biological_process | cellular response to heat | 0.0008212 |  | X |  |  |  |  |
| GO:0010596 | biological_process | negative regulation of endothelial cell migration | 0.00092686 | X | X |  |  | X |  |
| GO:0048512 | biological_process | circadian behavior | 0.00092686 |  |  |  | X |  |  |
| GO:0001937 | biological_process | negative regulation of endothelial cell proliferation | 0.0010357 | X | X |  |  |  |  |
| GO:0032369 | biological_process | negative regulation of lipid transport | 0.0010357 |  | X |  | X |  |  |
| GO:0010595 | biological_process | positive regulation of endothelial cell migration | 0.0010357 |  | X |  |  |  |  |
| GO:0002687 | biological_process | positive regulation of leukocyte migration | 0.00114742 |  | X |  |  | X |  |
| GO:0032655 | biological_process | regulation of interleukin-12 production | 0.00114742 |  | X |  |  | X |  |
| GO:0007622 | biological_process | rhythmic behavior | 0.00114742 |  |  |  | X |  |  |
| GO:0043535 | biological_process | regulation of blood vessel endothelial cell migration | 0.00141902 | X | X |  |  |  |  |
| GO:0010038 | biological_process | response to metal ion | 0.00164313 | X |  |  |  |  | X |
| GO:0032570 | biological_process | response to progesterone stimulus | 0.0016632 | X | X |  |  |  |  |
| GO:0006692 | biological_process | prostanoid metabolic process | 0.0016632 |  | X | X | X |  |  |
| GO:0006693 | biological_process | prostaglandin metabolic process | 0.0016632 |  | X | X | X |  |  |
| GO:0030431 | biological_process | sleep | 0.0016632 |  |  |  | X |  |  |
| GO:0002685 | biological_process | regulation of leukocyte migration | 0.00177205 | X |  |  |  | X |  |
| GO:0042752 | biological_process | regulation of circadian rhythm | 0.00177205 |  |  |  | X | X |  |
| GO:0016525 | biological_process | negative regulation of angiogenesis | 0.00207786 |  |  |  | X |  | X |
| GO:0045727 | biological_process | positive regulation of translation | 0.00207786 |  |  |  |  |  |  |
| GO:0010035 | biological_process | response to inorganic substance | 0.00217067 | X | X |  |  |  | X |
| GO:0045766 | biological_process | positive regulation of angiogenesis | 0.00237951 |  |  |  |  |  |  |
| GO:0001952 | biological_process | regulation of cell-matrix adhesion | 0.00251361 |  |  |  |  | X | X |
| GO:0010811 | biological_process | positive regulation of cell-substrate adhesion | 0.00251361 |  |  |  |  |  |  |
| GO:0018149 | biological_process | peptide cross-linking | 0.00251361 |  |  |  |  |  |  |
| GO:0010594 | biological_process | regulation of endothelial cell migration | 0.00269176 |  |  |  |  | X |  |
| GO:0040017 | biological_process | positive regulation of locomotion | 0.00298999 |  |  |  |  | X |  |
| GO:0050921 | biological_process | positive regulation of chemotaxis | 0.00298999 |  |  |  |  | X |  |
| GO:0001817 | biological_process | regulation of cytokine production | 0.00298999 |  |  |  |  |  |  |
| GO:0001936 | biological_process | regulation of endothelial cell proliferation | 0.00298999 |  |  |  |  |  |  |
| GO:0030193 | biological_process | regulation of blood coagulation | 0.00312918 |  | X |  | X |  |  |
| GO:0046456 | biological_process | icosanoid biosynthetic process | 0.00312918 |  |  |  | X |  |  |
| GO:0032368 | biological_process | regulation of lipid transport | 0.00312918 |  |  |  |  |  |  |
| GO:0050920 | biological_process | regulation of chemotaxis | 0.00331858 |  |  |  |  | X |  |
| GO:0006636 | biological_process | unsaturated fatty acid biosynthetic process | 0.00373744 |  |  |  | X |  |  |
| GO:0050818 | biological_process | regulation of coagulation | 0.00388218 |  | X |  | X |  |  |
| GO:0048520 | biological_process | positive regulation of behavior | 0.00388218 |  |  |  |  |  |  |
| GO:0001818 | biological_process | negative regulation of cytokine production | 0.00423237 |  | X |  |  |  |  |
| GO:0090100 | biological_process | positive regulation of transmembrane receptor protein serine/threonine kinase signaling pathway | 0.00423237 |  | X |  |  |  |  |
| GO:0007162 | biological_process | negative regulation of cell adhesion | 0.00493799 | X |  |  |  | X | X |
| GO:0017015 | biological_process | regulation of transforming growth factor beta receptor signaling pathway | 0.00512353 |  | X |  |  |  |  |
| GO:0009968 | biological_process | negative regulation of signal transduction | 0.00521828 |  |  |  | X |  |  |
| GO:0009749 | biological_process | response to glucose stimulus | 0.00531027 |  |  |  |  |  |  |
| GO:0010810 | biological_process | regulation of cell-substrate adhesion | 0.00549814 | X |  |  |  | X | X |
| GO:0009746 | biological_process | response to hexose stimulus | 0.00561178 |  | X |  |  |  |  |
| GO:0034284 | biological_process | response to monosaccharide stimulus | 0.00561178 |  | X |  |  |  |  |
| GO:0007623 | biological_process | circadian rhythm | 0.00561178 |  |  |  | X |  |  |
| GO:0006909 | biological_process | phagocytosis | 0.00583845 |  |  |  | X |  |  |
| GO:0010648 | biological_process | negative regulation of cell communication | 0.00673072 |  |  |  | X |  |  |
| GO:0006690 | biological_process | icosanoid metabolic process | 0.00677444 |  |  |  | X |  |  |
| GO:0010557 | biological_process | positive regulation of macromolecule biosynthetic process | 0.00690845 |  | X |  |  |  |  |
| GO:0033559 | biological_process | unsaturated fatty acid metabolic process | 0.00755303 |  |  |  | X |  |  |
| GO:0030336 | biological_process | negative regulation of cell migration | 0.00831678 | X |  |  |  | X |  |
| GO:0031328 | biological_process | positive regulation of cellular biosynthetic process | 0.00831678 |  | X |  |  |  |  |
| GO:0009891 | biological_process | positive regulation of biosynthetic process | 0.00849423 |  | X |  |  |  |  |
| GO:0009408 | biological_process | response to heat | 0.00849423 |  |  |  |  |  |  |
| GO:0009743 | biological_process | response to carbohydrate stimulus | 0.00866921 |  | X |  |  |  |  |
| GO:0045785 | biological_process | positive regulation of cell adhesion | 0.00866921 |  |  |  |  |  |  |
| GO:0051592 | biological_process | response to calcium ion | 0.00918862 | X |  |  | X |  | X |
| GO:0051271 | biological_process | negative regulation of cellular component movement | 0.00945169 | X |  |  |  | X |  |
| GO:0045765 | biological_process | regulation of angiogenesis | 0.01031386 |  |  |  |  |  | X |
| GO:0032103 | biological_process | positive regulation of response to external stimulus | 0.01058667 |  |  |  | X | X |  |
| GO:0010604 | biological_process | positive regulation of macromolecule metabolic process | 0.01331148 |  | X |  |  |  |  |
| GO:0031325 | biological_process | positive regulation of cellular metabolic process | 0.01506371 |  | X |  |  |  |  |
| GO:0006633 | biological_process | fatty acid biosynthetic process | 0.01543064 |  |  |  | X |  |  |
| GO:0000187 | biological_process | activation of MAPK activity | 0.01543064 |  |  |  |  |  |  |
| GO:0009266 | biological_process | response to temperature stimulus | 0.01636951 |  |  |  |  |  |  |
| GO:0090092 | biological_process | regulation of transmembrane receptor protein serine/threonine kinase signaling pathway | 0.01636951 |  |  |  |  |  |  |
| GO:0009893 | biological_process | positive regulation of metabolic process | 0.01679933 |  | X |  |  |  |  |
| GO:0002682 | biological_process | regulation of immune system process | 0.01866427 | X |  |  |  | X |  |
| GO:0001819 | biological_process | positive regulation of cytokine production | 0.01891283 |  |  |  |  |  |  |
| GO:0030335 | biological_process | positive regulation of cell migration | 0.01967725 | X |  |  |  |  |  |
| GO:0051239 | biological_process | regulation of multicellular organismal process | 0.0206055 |  | X |  | X |  | X |
| GO:0042327 | biological_process | positive regulation of phosphorylation | 0.02215168 |  |  |  |  |  |  |
| GO:0043406 | biological_process | positive regulation of MAP kinase activity | 0.02249475 |  |  |  |  |  |  |
| GO:0051272 | biological_process | positive regulation of cellular component movement | 0.02306172 | X |  |  |  |  |  |
| GO:0010562 | biological_process | positive regulation of phosphorus metabolic process | 0.02306172 |  |  |  |  |  |  |
| GO:0045937 | biological_process | positive regulation of phosphate metabolic process | 0.02306172 |  |  |  |  |  |  |
| GO:0002696 | biological_process | positive regulation of leukocyte activation | 0.02518155 |  |  |  |  | X |  |
| GO:0007050 | biological_process | cell cycle arrest | 0.02700958 |  |  |  |  |  | X |
| GO:0050867 | biological_process | positive regulation of cell activation | 0.0273685 |  |  |  |  | X |  |
| GO:0048583 | biological_process | regulation of response to stimulus | 0.02804896 | X |  |  | X |  |  |
| GO:0022900 | biological_process | electron transport chain | 0.03124453 |  |  |  |  |  |  |
| GO:0045893 | biological_process | positive regulation of transcription, DNA-dependent | 0.03219364 |  | X |  |  |  |  |
| GO:0051254 | biological_process | positive regulation of RNA metabolic process | 0.03283358 |  | X |  |  |  |  |
| GO:0051248 | biological_process | negative regulation of protein metabolic process | 0.03288801 |  |  |  | X |  |  |
| GO:0048511 | biological_process | rhythmic process | 0.03434254 |  |  |  | X |  |  |
| GO:0001525 | biological_process | angiogenesis | 0.03547219 | X | X |  | X |  |  |
| GO:0030155 | biological_process | regulation of cell adhesion | 0.03547219 | X |  |  |  |  | X |
| GO:0044087 | biological_process | regulation of cellular component biogenesis | 0.03695924 | X |  |  |  | X |  |
| GO:0051051 | biological_process | negative regulation of transport | 0.03716109 |  | X |  |  |  | X |
| GO:0001666 | biological_process | response to hypoxia | 0.03830187 |  | X |  |  |  |  |
| GO:0043405 | biological_process | regulation of MAP kinase activity | 0.03830187 |  |  |  |  |  |  |
| GO:0006417 | biological_process | regulation of translation | 0.03981609 |  |  |  | X | X |  |
| GO:0070482 | biological_process | response to oxygen levels | 0.04173751 | X | X |  |  |  |  |
| GO:0032101 | biological_process | regulation of response to external stimulus | 0.04448195 |  | X |  | X | X | X |
| GO:0051129 | biological_process | negative regulation of cellular component organization | 0.04487319 | X |  |  |  | X |  |
| GO:0051241 | biological_process | negative regulation of multicellular organismal process | 0.04526462 |  | X |  | X |  |  |
| GO:0016053 | biological_process | organic acid biosynthetic process | 0.04604799 |  |  |  | X |  |  |
| GO:0046394 | biological_process | carboxylic acid biosynthetic process | 0.04604799 |  |  |  | X |  |  |
| GO:0045941 | biological_process | positive regulation of transcription | 0.04639952 |  | X |  |  |  |  |
| GO:0010628 | biological_process | positive regulation of gene expression | 0.04994409 |  | X |  |  |  |  |
| GO:0005623 | cellular_component | cell | 0 |  | X |  |  | X |  |
| GO:0044464 | cellular_component | cell part | 0 |  | X |  |  | X |  |
| GO:0042613 | cellular_component | MHC class II protein complex | 1.5366E-09 |  |  |  |  |  |  |
| GO:0042611 | cellular_component | MHC protein complex | 8.985E-07 |  |  |  |  |  |  |
| GO:0005577 | cellular_component | fibrinogen complex | 0.00033791 |  | X |  |  |  |  |
| GO:0044459 | cellular_component | plasma membrane part | 0.00123374 |  |  |  |  |  |  |
| GO:0005791 | cellular_component | rough endoplasmic reticulum | 0.0016632 |  |  |  | X | X |  |
| GO:0031526 | cellular_component | brush border membrane | 0.00237951 |  |  |  |  |  |  |
| GO:0031093 | cellular_component | platelet alpha granule lumen | 0.00549814 | X |  |  | X |  |  |
| GO:0005903 | cellular_component | brush border | 0.00561178 |  |  |  |  |  |  |
| GO:0060205 | cellular_component | cytoplasmic membrane-bounded vesicle lumen | 0.00606811 | X |  |  | X |  |  |
| GO:0031983 | cellular_component | vesicle lumen | 0.00657861 | X |  |  | X |  |  |
| GO:0031091 | cellular_component | platelet alpha granule | 0.00849423 | X |  |  | X |  |  |
| GO:0031253 | cellular_component | cell projection membrane | 0.01031386 |  |  |  |  |  |  |
| GO:0031965 | cellular_component | nuclear membrane | 0.01614582 |  |  |  | X | X |  |
| GO:0043234 | cellular_component | protein complex | 0.02804896 |  |  |  |  | X |  |
| GO:0005886 | cellular_component | plasma membrane | 0.03495442 |  |  |  |  |  |  |
| GO:0009897 | cellular_component | external side of plasma membrane | 0.03716109 | X |  |  |  |  |  |
| GO:0016723 | molecular_function | oxidoreductase activity, oxidizing metal ions, NAD or NADP as acceptor | 0.00019388 | X |  |  |  |  |  |
| GO:0001968 | molecular_function | fibronectin binding | 0.00033791 |  | X |  |  |  |  |
| GO:0017134 | molecular_function | fibroblast growth factor binding | 0.00043564 |  | X |  |  |  |  |
| GO:0016722 | molecular_function | oxidoreductase activity, oxidizing metal ions | 0.00049555 |  |  |  |  |  |  |
| GO:0043236 | molecular_function | laminin binding | 0.00065185 |  | X |  |  |  |  |
| GO:0043394 | molecular_function | proteoglycan binding | 0.00072742 |  | X |  |  |  |  |
| GO:0050431 | molecular_function | transforming growth factor beta binding | 0.0008212 |  | X |  |  | X |  |
| GO:0043499 | molecular_function | eukaryotic cell surface binding | 0.00127425 | X | X |  |  |  |  |
| GO:0005501 | molecular_function | retinoid binding | 0.00177205 |  |  |  | X |  |  |
| GO:0030169 | molecular_function | low-density lipoprotein binding | 0.00177205 |  |  |  |  |  |  |
| GO:0019840 | molecular_function | isoprenoid binding | 0.00207786 |  |  |  | X |  |  |
| GO:0050840 | molecular_function | extracellular matrix binding | 0.00222729 |  |  |  |  |  |  |
| GO:0043498 | molecular_function | cell surface binding | 0.00298999 | X |  |  |  |  |  |
| GO:0001948 | molecular_function | glycoprotein binding | 0.00388218 |  |  |  |  |  |  |
| GO:0005518 | molecular_function | collagen binding | 0.00405648 |  | X |  | X |  |  |
| GO:0008034 | molecular_function | lipoprotein binding | 0.00405648 |  |  |  |  |  |  |
| GO:0016860 | molecular_function | intramolecular oxidoreductase activity | 0.00512353 |  |  |  | X |  |  |
| GO:0008170 | molecular_function | N-methyltransferase activity | 0.00810662 |  |  |  |  |  |  |
| GO:0005178 | molecular_function | integrin binding | 0.00918862 | X |  |  |  |  |  |
| GO:0008757 | molecular_function | S-adenosylmethionine-dependent methyltransferase activity | 0.01783567 |  |  |  |  |  |  |
| GO:0008201 | molecular_function | heparin binding | 0.02518155 |  |  |  |  |  |  |
| GO:0019838 | molecular_function | growth factor binding | 0.02772813 | X |  |  |  |  |  |
| GO:0019955 | molecular_function | cytokine binding | 0.02830577 |  |  |  |  |  |  |
| GO:0016853 | molecular_function | isomerase activity | 0.03754099 |  |  |  | X |  |  |
| GO:0005539 | molecular_function | glycosaminoglycan binding | 0.04135111 |  |  |  |  |  |  |
| GO:0001871 | molecular_function | pattern binding | 0.04783021 |  |  |  |  |  |  |
| GO:0030247 | molecular_function | polysaccharide binding | 0.04783021 |  |  |  |  |  |  |
