# Supplementary material for: Genes and Gene Ontologies Common to Airflow Obstruction and Emphysema in the Lungs of Patients with COPD
Source: PLoS One. 2011 Mar 15;6(3):e17442. doi: 10.1371/journal.pone.0017442 (PMC3057973; doi:10.1371/journal.pone.0017442)
Supplement: Table S4 — Gene ontologies enriched in Ning et al FEV1 dataset. (DOCX) [file pone.0017442.s006.docx]

**Table S4: Gene ontologies enriched in Ning *et al* dataset differentiating GOLD 0 from GOLD2 smokers.**

| **GOID** | **Ontology** | **Term** | **p** |
| --- | --- | --- | --- |
| GO:0006414 | biological_process | translational elongation | 1.43E-24 |
| GO:0006412 | biological_process | translation | 2.89E-19 |
| GO:0043284 | biological_process | biopolymer biosynthetic process | 1.11E-11 |
| GO:0034961 | biological_process | cellular biopolymer biosynthetic process | 3.21E-11 |
| GO:0009059 | biological_process | macromolecule biosynthetic process | 1.01E-10 |
| GO:0009058 | biological_process | biosynthetic process | 3.86E-10 |
| GO:0034645 | biological_process | cellular macromolecule biosynthetic process | 2.67E-09 |
| GO:0044249 | biological_process | cellular biosynthetic process | 4.75E-09 |
| GO:0009987 | biological_process | cellular process | 5.78E-09 |
| GO:0010467 | biological_process | gene expression | 7.06E-09 |
| GO:0044237 | biological_process | cellular metabolic process | 7.60E-08 |
| GO:0008152 | biological_process | metabolic process | 2.18E-07 |
| GO:0043170 | biological_process | macromolecule metabolic process | 2.18E-07 |
| GO:0044260 | biological_process | cellular macromolecule metabolic process | 1.44E-06 |
| GO:0044238 | biological_process | primary metabolic process | 1.77E-06 |
| GO:0019538 | biological_process | protein metabolic process | 2.47E-06 |
| GO:0044267 | biological_process | cellular protein metabolic process | 7.74E-06 |
| GO:0010035 | biological_process | response to inorganic substance | 8.88E-06 |
| GO:0043283 | biological_process | biopolymer metabolic process | 3.69E-05 |
| GO:0006172 | biological_process | ADP biosynthetic process | 4.18E-05 |
| GO:0009136 | biological_process | purine nucleoside diphosphate biosynthetic process | 4.18E-05 |
| GO:0009180 | biological_process | purine ribonucleoside diphosphate biosynthetic process | 4.18E-05 |
| GO:0009188 | biological_process | ribonucleoside diphosphate biosynthetic process | 4.18E-05 |
| GO:0016043 | biological_process | cellular component organization | 4.79E-05 |
| GO:0042274 | biological_process | ribosomal small subunit biogenesis | 4.99E-05 |
| GO:0044085 | biological_process | cellular component biogenesis | 7.38E-05 |
| GO:0046031 | biological_process | ADP metabolic process | 0.000116 |
| GO:0034960 | biological_process | cellular biopolymer metabolic process | 0.000173 |
| GO:0009133 | biological_process | nucleoside diphosphate biosynthetic process | 0.000256 |
| GO:0009135 | biological_process | purine nucleoside diphosphate metabolic process | 0.000256 |
| GO:0009179 | biological_process | purine ribonucleoside diphosphate metabolic process | 0.000256 |
| GO:0030155 | biological_process | regulation of cell adhesion | 0.000383 |
| GO:0022613 | biological_process | ribonucleoprotein complex biogenesis | 0.000383 |
| GO:0016192 | biological_process | vesicle-mediated transport | 0.00079 |
| GO:0033003 | biological_process | regulation of mast cell activation | 0.000823 |
| GO:0009185 | biological_process | ribonucleoside diphosphate metabolic process | 0.000823 |
| GO:0060325 | biological_process | face morphogenesis | 0.000823 |
| GO:0002886 | biological_process | regulation of myeloid leukocyte mediated immunity | 0.000823 |
| GO:0044092 | biological_process | negative regulation of molecular function | 0.00111 |
| GO:0006364 | biological_process | rRNA processing | 0.00111 |
| GO:0042273 | biological_process | ribosomal large subunit biogenesis | 0.001302 |
| GO:0002697 | biological_process | regulation of immune effector process | 0.001398 |
| GO:0016072 | biological_process | rRNA metabolic process | 0.001504 |
| GO:0042254 | biological_process | ribosome biogenesis | 0.001709 |
| GO:0048545 | biological_process | response to steroid hormone stimulus | 0.001741 |
| GO:0000302 | biological_process | response to reactive oxygen species | 0.001791 |
| GO:0010926 | biological_process | anatomical structure formation | 0.001797 |
| GO:0030260 | biological_process | entry into host cell | 0.001803 |
| GO:0044409 | biological_process | entry into host | 0.001803 |
| GO:0046718 | biological_process | entry of virus into host cell | 0.001803 |
| GO:0051806 | biological_process | entry into cell of other organism during symbiotic interaction | 0.001803 |
| GO:0051828 | biological_process | entry into other organism during symbiotic interaction | 0.001803 |
| GO:0052126 | biological_process | movement in host environment | 0.001803 |
| GO:0052192 | biological_process | movement in environment of other organism during symbiotic interaction | 0.001803 |
| GO:0009725 | biological_process | response to hormone stimulus | 0.001803 |
| GO:0060323 | biological_process | head morphogenesis | 0.001803 |
| GO:0060324 | biological_process | face development | 0.001803 |
| GO:0051179 | biological_process | localization | 0.002248 |
| GO:0010033 | biological_process | response to organic substance | 0.002379 |
| GO:0042221 | biological_process | response to chemical stimulus | 0.00244 |
| GO:0007162 | biological_process | negative regulation of cell adhesion | 0.002479 |
| GO:0016044 | biological_process | membrane organization | 0.002644 |
| GO:0010038 | biological_process | response to metal ion | 0.002981 |
| GO:0002246 | biological_process | healing during inflammatory response | 0.00383 |
| GO:0032964 | biological_process | collagen biosynthetic process | 0.00383 |
| GO:0006787 | biological_process | porphyrin catabolic process | 0.00383 |
| GO:0033015 | biological_process | tetrapyrrole catabolic process | 0.00383 |
| GO:0044275 | biological_process | cellular carbohydrate catabolic process | 0.004204 |
| GO:0032410 | biological_process | negative regulation of transporter activity | 0.004771 |
| GO:0010171 | biological_process | body morphogenesis | 0.004771 |
| GO:0060322 | biological_process | head development | 0.004771 |
| GO:0009719 | biological_process | response to endogenous stimulus | 0.005017 |
| GO:0009605 | biological_process | response to external stimulus | 0.00507 |
| GO:0006091 | biological_process | generation of precursor metabolites and energy | 0.00538 |
| GO:0006200 | biological_process | ATP catabolic process | 0.00623 |
| GO:0006901 | biological_process | vesicle coating | 0.00623 |
| GO:0009612 | biological_process | response to mechanical stimulus | 0.00664 |
| GO:0019062 | biological_process | virion attachment to host cell surface receptor | 0.007026 |
| GO:0030198 | biological_process | extracellular matrix organization | 0.007472 |
| GO:0030837 | biological_process | negative regulation of actin filament polymerization | 0.007562 |
| GO:0009132 | biological_process | nucleoside diphosphate metabolic process | 0.007963 |
| GO:0006900 | biological_process | membrane budding | 0.007963 |
| GO:0030833 | biological_process | regulation of actin filament polymerization | 0.008187 |
| GO:0032272 | biological_process | negative regulation of protein polymerization | 0.008732 |
| GO:0001568 | biological_process | blood vessel development | 0.009191 |
| GO:0030036 | biological_process | actin cytoskeleton organization | 0.009191 |
| GO:0022607 | biological_process | cellular component assembly | 0.010464 |
| GO:0051641 | biological_process | cellular localization | 0.011145 |
| GO:0033627 | biological_process | cell adhesion mediated by integrin | 0.01123 |
| GO:0043304 | biological_process | regulation of mast cell degranulation | 0.01123 |
| GO:0032365 | biological_process | intracellular lipid transport | 0.01123 |
| GO:0032409 | biological_process | regulation of transporter activity | 0.01129 |
| GO:0001944 | biological_process | vasculature development | 0.011323 |
| GO:0006007 | biological_process | glucose catabolic process | 0.011965 |
| GO:0009203 | biological_process | ribonucleoside triphosphate catabolic process | 0.012045 |
| GO:0009207 | biological_process | purine ribonucleoside triphosphate catabolic process | 0.012045 |
| GO:0009611 | biological_process | response to wounding | 0.012322 |
| GO:0006950 | biological_process | response to stress | 0.013211 |
| GO:0002703 | biological_process | regulation of leukocyte mediated immunity | 0.014215 |
| GO:0051494 | biological_process | negative regulation of cytoskeleton organization | 0.014215 |
| GO:0034660 | biological_process | ncRNA metabolic process | 0.014411 |
| GO:0051701 | biological_process | interaction with host | 0.014866 |
| GO:0051702 | biological_process | interaction with symbiont | 0.014866 |
| GO:0048519 | biological_process | negative regulation of biological process | 0.014866 |
| GO:0006810 | biological_process | transport | 0.014866 |
| GO:0046907 | biological_process | intracellular transport | 0.014866 |
| GO:0009146 | biological_process | purine nucleoside triphosphate catabolic process | 0.014866 |
| GO:0010714 | biological_process | positive regulation of collagen metabolic process | 0.014866 |
| GO:0032967 | biological_process | positive regulation of collagen biosynthetic process | 0.014866 |
| GO:0051271 | biological_process | negative regulation of cellular component movement | 0.014866 |
| GO:0030029 | biological_process | actin filament-based process | 0.014866 |
| GO:0008064 | biological_process | regulation of actin polymerization or depolymerization | 0.014866 |
| GO:0030832 | biological_process | regulation of actin filament length | 0.014866 |
| GO:0031333 | biological_process | negative regulation of protein complex assembly | 0.014866 |
| GO:0032271 | biological_process | regulation of protein polymerization | 0.014866 |
| GO:0032414 | biological_process | positive regulation of ion transmembrane transporter activity | 0.014866 |
| GO:0042542 | biological_process | response to hydrogen peroxide | 0.014866 |
| GO:0051591 | biological_process | response to cAMP | 0.014866 |
| GO:0055002 | biological_process | striated muscle cell development | 0.014866 |
| GO:0032413 | biological_process | negative regulation of ion transmembrane transporter activity | 0.014866 |
| GO:0060314 | biological_process | regulation of ryanodine-sensitive calcium-release channel activity | 0.014866 |
| GO:0034599 | biological_process | cellular response to oxidative stress | 0.014866 |
| GO:0043300 | biological_process | regulation of leukocyte degranulation | 0.014866 |
| GO:0001935 | biological_process | endothelial cell proliferation | 0.014866 |
| GO:0010639 | biological_process | negative regulation of organelle organization | 0.015499 |
| GO:0002714 | biological_process | positive regulation of B cell mediated immunity | 0.016143 |
| GO:0002891 | biological_process | positive regulation of immunoglobulin mediated immune response | 0.016143 |
| GO:0042060 | biological_process | wound healing | 0.016143 |
| GO:0001558 | biological_process | regulation of cell growth | 0.016143 |
| GO:0006098 | biological_process | pentose-phosphate shunt | 0.016143 |
| GO:0043537 | biological_process | negative regulation of blood vessel endothelial cell migration | 0.016143 |
| GO:0055093 | biological_process | response to hyperoxia | 0.016143 |
| GO:0002712 | biological_process | regulation of B cell mediated immunity | 0.016948 |
| GO:0002889 | biological_process | regulation of immunoglobulin mediated immune response | 0.016948 |
| GO:0019059 | biological_process | initiation of viral infection | 0.016948 |
| GO:0009154 | biological_process | purine ribonucleotide catabolic process | 0.016948 |
| GO:0022407 | biological_process | regulation of cell-cell adhesion | 0.016948 |
| GO:0051693 | biological_process | actin filament capping | 0.016948 |
| GO:0043242 | biological_process | negative regulation of protein complex disassembly | 0.017281 |
| GO:0050776 | biological_process | regulation of immune response | 0.017461 |
| GO:0051234 | biological_process | establishment of localization | 0.017669 |
| GO:0051129 | biological_process | negative regulation of cellular component organization | 0.018238 |
| GO:0019320 | biological_process | hexose catabolic process | 0.018795 |
| GO:0044403 | biological_process | symbiosis, encompassing mutualism through parasitism | 0.019018 |
| GO:0043062 | biological_process | extracellular structure organization | 0.019018 |
| GO:0015985 | biological_process | energy coupled proton transport, down electrochemical gradient | 0.019018 |
| GO:0015986 | biological_process | ATP synthesis coupled proton transport | 0.019018 |
| GO:0009143 | biological_process | nucleoside triphosphate catabolic process | 0.019553 |
| GO:0006754 | biological_process | ATP biosynthetic process | 0.020257 |
| GO:0051101 | biological_process | regulation of DNA binding | 0.020621 |
| GO:0051270 | biological_process | regulation of cellular component movement | 0.020799 |
| GO:0006740 | biological_process | NADPH regeneration | 0.020799 |
| GO:0032411 | biological_process | positive regulation of transporter activity | 0.020799 |
| GO:0043433 | biological_process | negative regulation of transcription factor activity | 0.020799 |
| GO:0090048 | biological_process | negative regulation of transcription regulator activity | 0.020799 |
| GO:0010812 | biological_process | negative regulation of cell-substrate adhesion | 0.020799 |
| GO:0048194 | biological_process | Golgi vesicle budding | 0.020799 |
| GO:0048200 | biological_process | Golgi transport vesicle coating | 0.020799 |
| GO:0048205 | biological_process | COPI coating of Golgi vesicle | 0.020799 |
| GO:0007010 | biological_process | cytoskeleton organization | 0.020969 |
| GO:0046365 | biological_process | monosaccharide catabolic process | 0.021004 |
| GO:0065003 | biological_process | macromolecular complex assembly | 0.021198 |
| GO:0008104 | biological_process | protein localization | 0.021418 |
| GO:0009261 | biological_process | ribonucleotide catabolic process | 0.021862 |
| GO:0050777 | biological_process | negative regulation of immune response | 0.021862 |
| GO:0030835 | biological_process | negative regulation of actin filament depolymerization | 0.021862 |
| GO:0032412 | biological_process | regulation of ion transmembrane transporter activity | 0.021862 |
| GO:0043933 | biological_process | macromolecular complex subunit organization | 0.021977 |
| GO:0034622 | biological_process | cellular macromolecular complex assembly | 0.021977 |
| GO:0033036 | biological_process | macromolecule localization | 0.022324 |
| GO:0051649 | biological_process | establishment of localization in cell | 0.022772 |
| GO:0007015 | biological_process | actin filament organization | 0.024076 |
| GO:0060627 | biological_process | regulation of vesicle-mediated transport | 0.024674 |
| GO:0048585 | biological_process | negative regulation of response to stimulus | 0.024674 |
| GO:0031032 | biological_process | actomyosin structure organization | 0.024887 |
| GO:0022898 | biological_process | regulation of transmembrane transporter activity | 0.024887 |
| GO:0034765 | biological_process | regulation of ion transmembrane transport | 0.024887 |
| GO:0055001 | biological_process | muscle cell development | 0.024887 |
| GO:0034614 | biological_process | cellular response to reactive oxygen species | 0.024887 |
| GO:0008285 | biological_process | negative regulation of cell proliferation | 0.025081 |
| GO:0032965 | biological_process | regulation of collagen biosynthetic process | 0.026092 |
| GO:0044253 | biological_process | positive regulation of multicellular organismal metabolic process | 0.026092 |
| GO:0022409 | biological_process | positive regulation of cell-cell adhesion | 0.026092 |
| GO:0007155 | biological_process | cell adhesion | 0.026679 |
| GO:0022610 | biological_process | biological adhesion | 0.027131 |
| GO:0051246 | biological_process | regulation of protein metabolic process | 0.029339 |
| GO:0051272 | biological_process | positive regulation of cellular component movement | 0.02993 |
| GO:0048583 | biological_process | regulation of response to stimulus | 0.030151 |
| GO:0030334 | biological_process | regulation of cell migration | 0.030151 |
| GO:0010712 | biological_process | regulation of collagen metabolic process | 0.030151 |
| GO:0030834 | biological_process | regulation of actin filament depolymerization | 0.030151 |
| GO:0006979 | biological_process | response to oxidative stress | 0.030151 |
| GO:0043392 | biological_process | negative regulation of DNA binding | 0.030151 |
| GO:0050727 | biological_process | regulation of inflammatory response | 0.030151 |
| GO:0048199 | biological_process | vesicle targeting, to, from or within Golgi | 0.030151 |
| GO:0051090 | biological_process | regulation of transcription factor activity | 0.030239 |
| GO:0090046 | biological_process | regulation of transcription regulator activity | 0.030239 |
| GO:0006119 | biological_process | oxidative phosphorylation | 0.030239 |
| GO:0009206 | biological_process | purine ribonucleoside triphosphate biosynthetic process | 0.030239 |
| GO:0090066 | biological_process | regulation of anatomical structure size | 0.031397 |
| GO:0009145 | biological_process | purine nucleoside triphosphate biosynthetic process | 0.031755 |
| GO:0009201 | biological_process | ribonucleoside triphosphate biosynthetic process | 0.031755 |
| GO:0016052 | biological_process | carbohydrate catabolic process | 0.031755 |
| GO:0032535 | biological_process | regulation of cellular component size | 0.03264 |
| GO:0065009 | biological_process | regulation of molecular function | 0.033735 |
| GO:0046164 | biological_process | alcohol catabolic process | 0.03425 |
| GO:0006898 | biological_process | receptor-mediated endocytosis | 0.034659 |
| GO:0009142 | biological_process | nucleoside triphosphate biosynthetic process | 0.035092 |
| GO:0050865 | biological_process | regulation of cell activation | 0.035262 |
| GO:0051668 | biological_process | localization within membrane | 0.035262 |
| GO:0043500 | biological_process | muscle adaptation | 0.035262 |
| GO:0046700 | biological_process | heterocycle catabolic process | 0.035954 |
| GO:0042476 | biological_process | odontogenesis | 0.037093 |
| GO:0043244 | biological_process | regulation of protein complex disassembly | 0.037093 |
| GO:0031347 | biological_process | regulation of defense response | 0.03742 |
| GO:0048731 | biological_process | system development | 0.037826 |
| GO:0046034 | biological_process | ATP metabolic process | 0.038235 |
| GO:0065007 | biological_process | biological regulation | 0.040556 |
| GO:0043623 | biological_process | cellular protein complex assembly | 0.040581 |
| GO:0002682 | biological_process | regulation of immune system process | 0.041208 |
| GO:0019216 | biological_process | regulation of lipid metabolic process | 0.042217 |
| GO:0009653 | biological_process | anatomical structure morphogenesis | 0.042573 |
| GO:0010596 | biological_process | negative regulation of endothelial cell migration | 0.042611 |
| GO:0051279 | biological_process | regulation of release of sequestered calcium ion into cytosol | 0.042611 |
| GO:0031960 | biological_process | response to corticosteroid stimulus | 0.042611 |
| GO:0043254 | biological_process | regulation of protein complex assembly | 0.042611 |
| GO:0006376 | biological_process | mRNA splice site selection | 0.042611 |
| GO:0030168 | biological_process | platelet activation | 0.042711 |
| GO:0034762 | biological_process | regulation of transmembrane transport | 0.042711 |
| GO:0006413 | biological_process | translational initiation | 0.042711 |
| GO:0070482 | biological_process | response to oxygen levels | 0.043202 |
| GO:0034220 | biological_process | ion transmembrane transport | 0.04584 |
| GO:0030336 | biological_process | negative regulation of cell migration | 0.04584 |
| GO:0006807 | biological_process | nitrogen compound metabolic process | 0.046188 |
| GO:0048856 | biological_process | anatomical structure development | 0.046188 |
| GO:0001937 | biological_process | negative regulation of endothelial cell proliferation | 0.046188 |
| GO:0006195 | biological_process | purine nucleotide catabolic process | 0.046188 |
| GO:0006006 | biological_process | glucose metabolic process | 0.046188 |
| GO:0006739 | biological_process | NADP metabolic process | 0.046188 |
| GO:0034621 | biological_process | cellular macromolecular complex subunit organization | 0.046188 |
| GO:0060021 | biological_process | palate development | 0.046188 |
| GO:0044246 | biological_process | regulation of multicellular organismal metabolic process | 0.046188 |
| GO:0048145 | biological_process | regulation of fibroblast proliferation | 0.046188 |
| GO:0003158 | biological_process | endothelium development | 0.046188 |
| GO:0045446 | biological_process | endothelial cell differentiation | 0.046188 |
| GO:0006984 | biological_process | ER-nuclear signaling pathway | 0.046188 |
| GO:0000097 | biological_process | sulfur amino acid biosynthetic process | 0.046188 |
| GO:0032088 | biological_process | negative regulation of NF-kappaB transcription factor activity | 0.046188 |
| GO:0002698 | biological_process | negative regulation of immune effector process | 0.046188 |
| GO:0051100 | biological_process | negative regulation of binding | 0.046252 |
| GO:0042127 | biological_process | regulation of cell proliferation | 0.047353 |
| GO:0040012 | biological_process | regulation of locomotion | 0.049357 |
| GO:0007229 | biological_process | integrin-mediated signaling pathway | 0.049879 |
| GO:0022626 | cellular_component | cytosolic ribosome | 5.92E-22 |
| GO:0005840 | cellular_component | ribosome | 1.83E-18 |
| GO:0033279 | cellular_component | ribosomal subunit | 3.59E-17 |
| GO:0005829 | cellular_component | cytosol | 5.51E-17 |
| GO:0044445 | cellular_component | cytosolic part | 3.26E-16 |
| GO:0044444 | cellular_component | cytoplasmic part | 1.69E-15 |
| GO:0005737 | cellular_component | cytoplasm | 1.07E-14 |
| GO:0030529 | cellular_component | ribonucleoprotein complex | 2.12E-12 |
| GO:0022627 | cellular_component | cytosolic small ribosomal subunit | 3.21E-11 |
| GO:0022625 | cellular_component | cytosolic large ribosomal subunit | 5.10E-10 |
| GO:0015934 | cellular_component | large ribosomal subunit | 1.39E-08 |
| GO:0015935 | cellular_component | small ribosomal subunit | 1.39E-08 |
| GO:0043228 | cellular_component | non-membrane-bounded organelle | 4.14E-07 |
| GO:0043232 | cellular_component | intracellular non-membrane-bounded organelle | 4.14E-07 |
| GO:0032991 | cellular_component | macromolecular complex | 6.87E-07 |
| GO:0044424 | cellular_component | intracellular part | 1.32E-06 |
| GO:0043229 | cellular_component | intracellular organelle | 1.29E-05 |
| GO:0043226 | cellular_component | organelle | 1.45E-05 |
| GO:0044446 | cellular_component | intracellular organelle part | 2.99E-05 |
| GO:0044422 | cellular_component | organelle part | 3.95E-05 |
| GO:0005622 | cellular_component | intracellular | 4.82E-05 |
| GO:0000275 | cellular_component | mitochondrial proton-transporting ATP synthase complex, catalytic core F(1) | 0.000116 |
| GO:0044421 | cellular_component | extracellular region part | 0.000163 |
| GO:0045261 | cellular_component | proton-transporting ATP synthase complex, catalytic core F(1) | 0.000256 |
| GO:0005578 | cellular_component | proteinaceous extracellular matrix | 0.000484 |
| GO:0031012 | cellular_component | extracellular matrix | 0.001193 |
| GO:0005753 | cellular_component | mitochondrial proton-transporting ATP synthase complex | 0.001228 |
| GO:0005581 | cellular_component | collagen | 0.001475 |
| GO:0045259 | cellular_component | proton-transporting ATP synthase complex | 0.001803 |
| GO:0009986 | cellular_component | cell surface | 0.005759 |
| GO:0031091 | cellular_component | platelet alpha granule | 0.014215 |
| GO:0033178 | cellular_component | proton-transporting two-sector ATPase complex, catalytic domain | 0.014411 |
| GO:0005615 | cellular_component | extracellular space | 0.014499 |
| GO:0044420 | cellular_component | extracellular matrix part | 0.014866 |
| GO:0016281 | cellular_component | eukaryotic translation initiation factor 4F complex | 0.016143 |
| GO:0005740 | cellular_component | mitochondrial envelope | 0.017234 |
| GO:0001725 | cellular_component | stress fiber | 0.019553 |
| GO:0016023 | cellular_component | cytoplasmic membrane-bounded vesicle | 0.019975 |
| GO:0031410 | cellular_component | cytoplasmic vesicle | 0.020257 |
| GO:0031093 | cellular_component | platelet alpha granule lumen | 0.020799 |
| GO:0032432 | cellular_component | actin filament bundle | 0.024887 |
| GO:0031966 | cellular_component | mitochondrial membrane | 0.025749 |
| GO:0044455 | cellular_component | mitochondrial membrane part | 0.026092 |
| GO:0060205 | cellular_component | cytoplasmic membrane-bounded vesicle lumen | 0.026092 |
| GO:0005583 | cellular_component | fibrillar collagen | 0.026092 |
| GO:0030126 | cellular_component | COPI vesicle coat | 0.026092 |
| GO:0031988 | cellular_component | membrane-bounded vesicle | 0.026522 |
| GO:0042641 | cellular_component | actomyosin | 0.028018 |
| GO:0016469 | cellular_component | proton-transporting two-sector ATPase complex | 0.028584 |
| GO:0031982 | cellular_component | vesicle | 0.030151 |
| GO:0031983 | cellular_component | vesicle lumen | 0.030151 |
| GO:0032587 | cellular_component | ruffle membrane | 0.030151 |
| GO:0030663 | cellular_component | COPI coated vesicle membrane | 0.030151 |
| GO:0030131 | cellular_component | clathrin adaptor complex | 0.031321 |
| GO:0031090 | cellular_component | organelle membrane | 0.034106 |
| GO:0030119 | cellular_component | AP-type membrane coat adaptor complex | 0.035092 |
| GO:0016581 | cellular_component | NuRD complex | 0.035262 |
| GO:0034362 | cellular_component | low-density lipoprotein particle | 0.035262 |
| GO:0000323 | cellular_component | lytic vacuole | 0.037826 |
| GO:0005764 | cellular_component | lysosome | 0.037826 |
| GO:0005576 | cellular_component | extracellular region | 0.04241 |
| GO:0005773 | cellular_component | vacuole | 0.042611 |
| GO:0030141 | cellular_component | secretory granule | 0.04584 |
| GO:0005743 | cellular_component | mitochondrial inner membrane | 0.046188 |
| GO:0030137 | cellular_component | COPI-coated vesicle | 0.046188 |
| GO:0003735 | molecular_function | structural constituent of ribosome | 4.58E-20 |
| GO:0005515 | molecular_function | protein binding | 2.08E-16 |
| GO:0005198 | molecular_function | structural molecule activity | 3.75E-14 |
| GO:0005488 | molecular_function | binding | 4.87E-08 |
| GO:0048407 | molecular_function | platelet-derived growth factor binding | 8.56E-07 |
| GO:0019838 | molecular_function | growth factor binding | 1.27E-06 |
| GO:0005178 | molecular_function | integrin binding | 2.77E-05 |
| GO:0032403 | molecular_function | protein complex binding | 3.48E-05 |
| GO:0003723 | molecular_function | RNA binding | 6.89E-05 |
| GO:0005102 | molecular_function | receptor binding | 7.38E-05 |
| GO:0019966 | molecular_function | interleukin-1 binding | 0.001302 |
| GO:0019899 | molecular_function | enzyme binding | 0.002052 |
| GO:0005520 | molecular_function | insulin-like growth factor binding | 0.003332 |
| GO:0046933 | molecular_function | hydrogen ion transporting ATP synthase activity, rotational mechanism | 0.004771 |
| GO:0019843 | molecular_function | rRNA binding | 0.004771 |
| GO:0047485 | molecular_function | protein N-terminus binding | 0.009769 |
| GO:0019865 | molecular_function | immunoglobulin binding | 0.009941 |
| GO:0043499 | molecular_function | eukaryotic cell surface binding | 0.009941 |
| GO:0008092 | molecular_function | cytoskeletal protein binding | 0.013889 |
| GO:0043531 | molecular_function | ADP binding | 0.014411 |
| GO:0046961 | molecular_function | proton-transporting ATPase activity, rotational mechanism | 0.014411 |
| GO:0019829 | molecular_function | cation-transporting ATPase activity | 0.017281 |
| GO:0042288 | molecular_function | MHC class I protein binding | 0.026092 |
| GO:0005161 | molecular_function | platelet-derived growth factor receptor binding | 0.026092 |
| GO:0043498 | molecular_function | cell surface binding | 0.035092 |
| GO:0051117 | molecular_function | ATPase binding | 0.035262 |
| GO:0008430 | molecular_function | selenium binding | 0.038854 |
| GO:0005509 | molecular_function | calcium ion binding | 0.041005 |
| GO:0003743 | molecular_function | translation initiation factor activity | 0.042611 |
| GO:0042803 | molecular_function | protein homodimerization activity | 0.046188 |
