# Supplementary material for: Genes and Gene Ontologies Common to Airflow Obstruction and Emphysema in the Lungs of Patients with COPD
Source: PLoS One. 2011 Mar 15;6(3):e17442. doi: 10.1371/journal.pone.0017442 (PMC3057973; doi:10.1371/journal.pone.0017442)
Supplement: Table S5 — Gene ontologies enriched in Wang et al FEF25–75% dataset. (DOCX) [file pone.0017442.s007.docx]

**Table S5: Gene ontologies enriched in Wang *et al* dataset using FEF_(25%-75%)_ as classification parameter.**

| GOID | Ontology | Term | p |
| --- | --- | --- | --- |
| GO:0048731 | biological_process | system development | 0.000216 |
| GO:0048856 | biological_process | anatomical structure development | 0.000229 |
| GO:0048513 | biological_process | organ development | 0.000373 |
| GO:0002448 | biological_process | mast cell mediated immunity | 0.000373 |
| GO:0043303 | biological_process | mast cell degranulation | 0.000373 |
| GO:0042551 | biological_process | neuron maturation | 0.00051 |
| GO:0042416 | biological_process | dopamine biosynthetic process | 0.000679 |
| GO:0002532 | biological_process | production of molecular mediator of acute inflammatory response | 0.000679 |
| GO:0043523 | biological_process | regulation of neuron apoptosis | 0.00086 |
| GO:0043524 | biological_process | negative regulation of neuron apoptosis | 0.000871 |
| GO:0046668 | biological_process | regulation of retinal cell programmed cell death | 0.001048 |
| GO:0008344 | biological_process | adult locomotory behavior | 0.001048 |
| GO:0017085 | biological_process | response to insecticide | 0.001048 |
| GO:0007185 | biological_process | transmembrane receptor protein tyrosine phosphatase signaling pathway | 0.001048 |
| GO:0007275 | biological_process | multicellular organismal development | 0.001338 |
| GO:0021700 | biological_process | developmental maturation | 0.001477 |
| GO:0042053 | biological_process | regulation of dopamine metabolic process | 0.001551 |
| GO:0045576 | biological_process | mast cell activation | 0.001551 |
| GO:0007566 | biological_process | embryo implantation | 0.001825 |
| GO:0048167 | biological_process | regulation of synaptic plasticity | 0.001933 |
| GO:0042069 | biological_process | regulation of catecholamine metabolic process | 0.0022 |
| GO:0032502 | biological_process | developmental process | 0.002574 |
| GO:0043403 | biological_process | skeletal muscle tissue regeneration | 0.00298 |
| GO:0043299 | biological_process | leukocyte degranulation | 0.00298 |
| GO:0009987 | biological_process | cellular process | 0.003136 |
| GO:0009653 | biological_process | anatomical structure morphogenesis | 0.003136 |
| GO:0009636 | biological_process | response to toxin | 0.003136 |
| GO:0021952 | biological_process | central nervous system projection neuron axonogenesis | 0.003136 |
| GO:0042423 | biological_process | catecholamine biosynthetic process | 0.003136 |
| GO:0043576 | biological_process | regulation of respiratory gaseous exchange | 0.003136 |
| GO:0002089 | biological_process | lens morphogenesis in camera-type eye | 0.003136 |
| GO:0033238 | biological_process | regulation of cellular amine metabolic process | 0.00411 |
| GO:0030511 | biological_process | positive regulation of transforming growth factor beta receptor signaling pathway | 0.00411 |
| GO:0030534 | biological_process | adult behavior | 0.00412 |
| GO:0021955 | biological_process | central nervous system neuron axonogenesis | 0.005023 |
| GO:0002444 | biological_process | myeloid leukocyte mediated immunity | 0.005023 |
| GO:0043066 | biological_process | negative regulation of apoptosis | 0.005469 |
| GO:0043069 | biological_process | negative regulation of programmed cell death | 0.005937 |
| GO:0060548 | biological_process | negative regulation of cell death | 0.006019 |
| GO:0001666 | biological_process | response to hypoxia | 0.006355 |
| GO:0010557 | biological_process | positive regulation of macromolecule biosynthetic process | 0.006867 |
| GO:0045944 | biological_process | positive regulation of transcription from RNA polymerase II promoter | 0.006867 |
| GO:0070482 | biological_process | response to oxygen levels | 0.006867 |
| GO:0001975 | biological_process | response to amphetamine | 0.006867 |
| GO:0045055 | biological_process | regulated secretory pathway | 0.006867 |
| GO:0045941 | biological_process | positive regulation of transcription | 0.007091 |
| GO:0042417 | biological_process | dopamine metabolic process | 0.007091 |
| GO:0045893 | biological_process | positive regulation of transcription, DNA-dependent | 0.008574 |
| GO:0010628 | biological_process | positive regulation of gene expression | 0.009022 |
| GO:0051254 | biological_process | positive regulation of RNA metabolic process | 0.009022 |
| GO:0048646 | biological_process | anatomical structure formation involved in morphogenesis | 0.009138 |
| GO:0050879 | biological_process | multicellular organismal movement | 0.009443 |
| GO:0050881 | biological_process | musculoskeletal movement | 0.009443 |
| GO:0009887 | biological_process | organ morphogenesis | 0.009614 |
| GO:0006950 | biological_process | response to stress | 0.009623 |
| GO:0031328 | biological_process | positive regulation of cellular biosynthetic process | 0.009626 |
| GO:0007154 | biological_process | cell communication | 0.009862 |
| GO:0048666 | biological_process | neuron development | 0.010396 |
| GO:0009891 | biological_process | positive regulation of biosynthetic process | 0.010396 |
| GO:0007599 | biological_process | hemostasis | 0.010396 |
| GO:0002088 | biological_process | lens development in camera-type eye | 0.010417 |
| GO:0007155 | biological_process | cell adhesion | 0.011746 |
| GO:0022610 | biological_process | biological adhesion | 0.011841 |
| GO:0045935 | biological_process | positive regulation of nucleobase, nucleoside, nucleotide and nucleic acid metabolic process | 0.013321 |
| GO:0008634 | biological_process | negative regulation of survival gene product expression | 0.015306 |
| GO:0014047 | biological_process | glutamate secretion | 0.015306 |
| GO:0021520 | biological_process | spinal cord motor neuron cell fate specification | 0.015306 |
| GO:0048934 | biological_process | peripheral nervous system neuron differentiation | 0.015306 |
| GO:0007253 | biological_process | cytoplasmic sequestering of NF-kappaB | 0.015306 |
| GO:0006999 | biological_process | nuclear pore organization | 0.015306 |
| GO:0010243 | biological_process | response to organic nitrogen | 0.015306 |
| GO:0014812 | biological_process | muscle cell migration | 0.015306 |
| GO:0010042 | biological_process | response to manganese ion | 0.015306 |
| GO:0002544 | biological_process | chronic inflammatory response | 0.015306 |
| GO:0051895 | biological_process | negative regulation of focal adhesion formation | 0.015306 |
| GO:0007494 | biological_process | midgut development | 0.015306 |
| GO:0032234 | biological_process | regulation of calcium ion transport via store-operated calcium channel activity | 0.015306 |
| GO:0051173 | biological_process | positive regulation of nitrogen compound metabolic process | 0.01598 |
| GO:0009605 | biological_process | response to external stimulus | 0.018491 |
| GO:0006839 | biological_process | mitochondrial transport | 0.018908 |
| GO:0007626 | biological_process | locomotory behavior | 0.019183 |
| GO:0050804 | biological_process | regulation of synaptic transmission | 0.019337 |
| GO:0007173 | biological_process | epidermal growth factor receptor signaling pathway | 0.019439 |
| GO:0007406 | biological_process | negative regulation of neuroblast proliferation | 0.019816 |
| GO:0006928 | biological_process | cellular component movement | 0.019816 |
| GO:0007610 | biological_process | behavior | 0.019816 |
| GO:0016584 | biological_process | nucleosome positioning | 0.019816 |
| GO:0017038 | biological_process | protein import | 0.019816 |
| GO:0019369 | biological_process | arachidonic acid metabolic process | 0.019816 |
| GO:0051968 | biological_process | positive regulation of synaptic transmission, glutamatergic | 0.019816 |
| GO:0006468 | biological_process | protein amino acid phosphorylation | 0.019816 |
| GO:0010953 | biological_process | regulation of protein maturation by peptide bond cleavage | 0.019816 |
| GO:0070613 | biological_process | regulation of protein processing | 0.019816 |
| GO:0030194 | biological_process | positive regulation of blood coagulation | 0.019816 |
| GO:0043277 | biological_process | apoptotic cell clearance | 0.019816 |
| GO:0051918 | biological_process | negative regulation of fibrinolysis | 0.019816 |
| GO:0032689 | biological_process | negative regulation of interferon-gamma production | 0.019816 |
| GO:0042921 | biological_process | glucocorticoid receptor signaling pathway | 0.019816 |
| GO:0048484 | biological_process | enteric nervous system development | 0.019816 |
| GO:0001736 | biological_process | establishment of planar polarity | 0.019816 |
| GO:0060395 | biological_process | SMAD protein signal transduction | 0.019816 |
| GO:0051093 | biological_process | negative regulation of developmental process | 0.019842 |
| GO:0042221 | biological_process | response to chemical stimulus | 0.020178 |
| GO:0009888 | biological_process | tissue development | 0.021272 |
| GO:0032940 | biological_process | secretion by cell | 0.021397 |
| GO:0051239 | biological_process | regulation of multicellular organismal process | 0.021397 |
| GO:0048469 | biological_process | cell maturation | 0.021611 |
| GO:0010604 | biological_process | positive regulation of macromolecule metabolic process | 0.022207 |
| GO:0001525 | biological_process | angiogenesis | 0.022382 |
| GO:0006916 | biological_process | anti-apoptosis | 0.022546 |
| GO:0021954 | biological_process | central nervous system neuron development | 0.022546 |
| GO:0042401 | biological_process | cellular biogenic amine biosynthetic process | 0.022546 |
| GO:0030193 | biological_process | regulation of blood coagulation | 0.022546 |
| GO:0042246 | biological_process | tissue regeneration | 0.022546 |
| GO:0048589 | biological_process | developmental growth | 0.023141 |
| GO:0051969 | biological_process | regulation of transmission of nerve impulse | 0.023307 |
| GO:0070227 | biological_process | lymphocyte apoptosis | 0.02381 |
| GO:0043496 | biological_process | regulation of protein homodimerization activity | 0.02381 |
| GO:0042347 | biological_process | negative regulation of NF-kappaB import into nucleus | 0.02381 |
| GO:0006584 | biological_process | catecholamine metabolic process | 0.02381 |
| GO:0009712 | biological_process | catechol metabolic process | 0.02381 |
| GO:0034311 | biological_process | diol metabolic process | 0.02381 |
| GO:0050878 | biological_process | regulation of body fluid levels | 0.02381 |
| GO:0016255 | biological_process | attachment of GPI anchor to protein | 0.02381 |
| GO:0032436 | biological_process | positive regulation of proteasomal ubiquitin-dependent protein catabolic process | 0.02381 |
| GO:0032891 | biological_process | negative regulation of organic acid transport | 0.02381 |
| GO:0032026 | biological_process | response to magnesium ion | 0.02381 |
| GO:0040036 | biological_process | regulation of fibroblast growth factor receptor signaling pathway | 0.02381 |
| GO:0031958 | biological_process | corticosteroid receptor signaling pathway | 0.02381 |
| GO:0007216 | biological_process | metabotropic glutamate receptor signaling pathway | 0.02381 |
| GO:0060041 | biological_process | retina development in camera-type eye | 0.02381 |
| GO:0006978 | biological_process | DNA damage response, signal transduction by p53 class mediator resulting in transcription of p21 class mediator | 0.02381 |
| GO:0042772 | biological_process | DNA damage response, signal transduction resulting in transcription | 0.02381 |
| GO:0033365 | biological_process | protein localization in organelle | 0.024628 |
| GO:0018958 | biological_process | phenol metabolic process | 0.024767 |
| GO:0007267 | biological_process | cell-cell signaling | 0.025498 |
| GO:0031325 | biological_process | positive regulation of cellular metabolic process | 0.026486 |
| GO:0031644 | biological_process | regulation of neurological system process | 0.02669 |
| GO:0002443 | biological_process | leukocyte mediated immunity | 0.02669 |
| GO:0006626 | biological_process | protein targeting to mitochondrion | 0.02669 |
| GO:0070585 | biological_process | protein localization in mitochondrion | 0.02669 |
| GO:0007423 | biological_process | sensory organ development | 0.027682 |
| GO:0003001 | biological_process | generation of a signal involved in cell-cell signaling | 0.027835 |
| GO:0042493 | biological_process | response to drug | 0.028191 |
| GO:0001657 | biological_process | ureteric bud development | 0.028564 |
| GO:0050818 | biological_process | regulation of coagulation | 0.028564 |
| GO:0008635 | biological_process | activation of caspase activity by cytochrome c | 0.029171 |
| GO:0043584 | biological_process | nose development | 0.029171 |
| GO:0042640 | biological_process | anagen | 0.029171 |
| GO:0060260 | biological_process | regulation of transcription initiation from RNA polymerase II promoter | 0.029171 |
| GO:0051893 | biological_process | regulation of focal adhesion formation | 0.029171 |
| GO:0032673 | biological_process | regulation of interleukin-4 production | 0.029171 |
| GO:0007164 | biological_process | establishment of tissue polarity | 0.029171 |
| GO:0032414 | biological_process | positive regulation of ion transmembrane transporter activity | 0.029171 |
| GO:0051972 | biological_process | regulation of telomerase activity | 0.029171 |
| GO:0035313 | biological_process | wound healing, spreading of epidermal cells | 0.029171 |
| GO:0006357 | biological_process | regulation of transcription from RNA polymerase II promoter | 0.029319 |
| GO:0014075 | biological_process | response to amine stimulus | 0.029725 |
| GO:0090100 | biological_process | positive regulation of transmembrane receptor protein serine/threonine kinase signaling pathway | 0.032002 |
| GO:0001818 | biological_process | negative regulation of cytokine production | 0.032002 |
| GO:0001568 | biological_process | blood vessel development | 0.032869 |
| GO:0009893 | biological_process | positive regulation of metabolic process | 0.033718 |
| GO:0032101 | biological_process | regulation of response to external stimulus | 0.033782 |
| GO:0070584 | biological_process | mitochondrion morphogenesis | 0.033956 |
| GO:0010926 | biological_process | anatomical structure formation | 0.033956 |
| GO:0030182 | biological_process | neuron differentiation | 0.033956 |
| GO:0009611 | biological_process | response to wounding | 0.033956 |
| GO:0006743 | biological_process | ubiquinone metabolic process | 0.033956 |
| GO:0006744 | biological_process | ubiquinone biosynthetic process | 0.033956 |
| GO:0045426 | biological_process | quinone cofactor biosynthetic process | 0.033956 |
| GO:0042730 | biological_process | fibrinolysis | 0.033956 |
| GO:0051241 | biological_process | negative regulation of multicellular organismal process | 0.033956 |
| GO:0002040 | biological_process | sprouting angiogenesis | 0.033956 |
| GO:0050820 | biological_process | positive regulation of coagulation | 0.033956 |
| GO:0043537 | biological_process | negative regulation of blood vessel endothelial cell migration | 0.033956 |
| GO:0006911 | biological_process | phagocytosis, engulfment | 0.033956 |
| GO:0001953 | biological_process | negative regulation of cell-matrix adhesion | 0.033956 |
| GO:0051917 | biological_process | regulation of fibrinolysis | 0.033956 |
| GO:0008211 | biological_process | glucocorticoid metabolic process | 0.033956 |
| GO:0042447 | biological_process | hormone catabolic process | 0.033956 |
| GO:0001738 | biological_process | morphogenesis of a polarized epithelium | 0.033956 |
| GO:0007080 | biological_process | mitotic metaphase plate congression | 0.033956 |
| GO:0014706 | biological_process | striated muscle tissue development | 0.034151 |
| GO:0021953 | biological_process | central nervous system neuron differentiation | 0.034151 |
| GO:0042303 | biological_process | molting cycle | 0.034151 |
| GO:0042633 | biological_process | hair cycle | 0.034151 |
| GO:0001838 | biological_process | embryonic epithelial tube formation | 0.034151 |
| GO:0001944 | biological_process | vasculature development | 0.034326 |
| GO:0043193 | biological_process | positive regulation of gene-specific transcription | 0.035457 |
| GO:0048518 | biological_process | positive regulation of biological process | 0.035603 |
| GO:0046879 | biological_process | hormone secretion | 0.036344 |
| GO:0010035 | biological_process | response to inorganic substance | 0.03655 |
| GO:0048699 | biological_process | generation of neurons | 0.037886 |
| GO:0007519 | biological_process | skeletal muscle tissue development | 0.037946 |
| GO:0060538 | biological_process | skeletal muscle organ development | 0.037946 |
| GO:0034329 | biological_process | cell junction assembly | 0.038489 |
| GO:0017015 | biological_process | regulation of transforming growth factor beta receptor signaling pathway | 0.038489 |
| GO:0002526 | biological_process | acute inflammatory response | 0.038753 |
| GO:0060537 | biological_process | muscle tissue development | 0.038753 |
| GO:0051085 | biological_process | chaperone mediated protein folding requiring cofactor | 0.038753 |
| GO:0032225 | biological_process | regulation of synaptic transmission, dopaminergic | 0.038753 |
| GO:0048820 | biological_process | hair follicle maturation | 0.038753 |
| GO:0010812 | biological_process | negative regulation of cell-substrate adhesion | 0.038753 |
| GO:0032411 | biological_process | positive regulation of transporter activity | 0.038753 |
| GO:0048593 | biological_process | camera-type eye morphogenesis | 0.039702 |
| GO:0007596 | biological_process | blood coagulation | 0.040745 |
| GO:0050817 | biological_process | coagulation | 0.040745 |
| GO:0007167 | biological_process | enzyme linked receptor protein signaling pathway | 0.040831 |
| GO:0001822 | biological_process | kidney development | 0.042077 |
| GO:0051789 | biological_process | response to protein stimulus | 0.042077 |
| GO:0051881 | biological_process | regulation of mitochondrial membrane potential | 0.043663 |
| GO:0001656 | biological_process | metanephros development | 0.043663 |
| GO:0007165 | biological_process | signal transduction | 0.043663 |
| GO:0042994 | biological_process | cytoplasmic sequestering of transcription factor | 0.043663 |
| GO:0009746 | biological_process | response to hexose stimulus | 0.043663 |
| GO:0034284 | biological_process | response to monosaccharide stimulus | 0.043663 |
| GO:0032434 | biological_process | regulation of proteasomal ubiquitin-dependent protein catabolic process | 0.043663 |
| GO:0080010 | biological_process | regulation of oxygen and reactive oxygen species metabolic process | 0.043663 |
| GO:0002690 | biological_process | positive regulation of leukocyte chemotaxis | 0.043663 |
| GO:0043030 | biological_process | regulation of macrophage activation | 0.043663 |
| GO:0043536 | biological_process | positive regulation of blood vessel endothelial cell migration | 0.043663 |
| GO:0048814 | biological_process | regulation of dendrite morphogenesis | 0.043663 |
| GO:0009914 | biological_process | hormone transport | 0.043663 |
| GO:0051310 | biological_process | metaphase plate congression | 0.043663 |
| GO:0032006 | biological_process | regulation of TOR signaling pathway | 0.043663 |
| GO:0043010 | biological_process | camera-type eye development | 0.045999 |
| GO:0050793 | biological_process | regulation of developmental process | 0.046792 |
| GO:0065007 | biological_process | biological regulation | 0.047481 |
| GO:0050768 | biological_process | negative regulation of neurogenesis | 0.047481 |
| GO:0002274 | biological_process | myeloid leukocyte activation | 0.047481 |
| GO:0006464 | biological_process | protein modification process | 0.04833 |
| GO:0008285 | biological_process | negative regulation of cell proliferation | 0.049032 |
| GO:0043687 | biological_process | post-translational protein modification | 0.049032 |
| GO:0001708 | biological_process | cell fate specification | 0.049032 |
| GO:0021522 | biological_process | spinal cord motor neuron differentiation | 0.049032 |
| GO:0051084 | biological_process | 'de novo' posttranslational protein folding | 0.049032 |
| GO:0016477 | biological_process | cell migration | 0.049032 |
| GO:0001516 | biological_process | prostaglandin biosynthetic process | 0.049032 |
| GO:0046457 | biological_process | prostanoid biosynthetic process | 0.049032 |
| GO:0030282 | biological_process | bone mineralization | 0.049032 |
| GO:0051926 | biological_process | negative regulation of calcium ion transport | 0.049032 |
| GO:0002688 | biological_process | regulation of leukocyte chemotaxis | 0.049032 |
| GO:0003009 | biological_process | skeletal muscle contraction | 0.049032 |
| GO:0005623 | cellular_component | cell | 0.010396 |
| GO:0044464 | cellular_component | cell part | 0.010396 |
| GO:0005922 | cellular_component | connexon complex | 0.010417 |
| GO:0030054 | cellular_component | cell junction | 0.015306 |
| GO:0012505 | cellular_component | endomembrane system | 0.019816 |
| GO:0005921 | cellular_component | gap junction | 0.019816 |
| GO:0014069 | cellular_component | postsynaptic density | 0.019816 |
| GO:0005593 | cellular_component | FACIT collagen | 0.019816 |
| GO:0005577 | cellular_component | fibrinogen complex | 0.02381 |
| GO:0030934 | cellular_component | anchoring collagen | 0.033956 |
| GO:0046930 | cellular_component | pore complex | 0.034151 |
| GO:0005912 | cellular_component | adherens junction | 0.034151 |
| GO:0031080 | cellular_component | Nup107-160 complex | 0.038753 |
| GO:0044456 | cellular_component | synapse part | 0.043663 |
| GO:0043034 | cellular_component | costamere | 0.043663 |
| GO:0005622 | cellular_component | intracellular | 0.044808 |
| GO:0070161 | cellular_component | anchoring junction | 0.046982 |
| GO:0031982 | cellular_component | vesicle | 0.049032 |
| GO:0005488 | molecular_function | binding | 0.000529 |
| GO:0016705 | molecular_function | oxidoreductase activity, acting on paired donors, with incorporation or reduction of molecular oxygen | 0.004543 |
| GO:0005001 | molecular_function | transmembrane receptor protein tyrosine phosphatase activity | 0.008363 |
| GO:0019198 | molecular_function | transmembrane receptor protein phosphatase activity | 0.008363 |
| GO:0003707 | molecular_function | steroid hormone receptor activity | 0.009022 |
| GO:0004879 | molecular_function | ligand-dependent nuclear receptor activity | 0.010396 |
| GO:0070513 | molecular_function | death domain binding | 0.015306 |
| GO:0070330 | molecular_function | aromatase activity | 0.015306 |
| GO:0017056 | molecular_function | structural constituent of nuclear pore | 0.015306 |
| GO:0035259 | molecular_function | glucocorticoid receptor binding | 0.015306 |
| GO:0015184 | molecular_function | L-cystine transmembrane transporter activity | 0.015306 |
| GO:0016712 | molecular_function | oxidoreductase activity, acting on paired donors, with incorporation or reduction of molecular oxygen, reduced flavin or flavoprotein as one donor, and incorporation of one atom of oxygen | 0.017748 |
| GO:0008020 | molecular_function | G-protein coupled photoreceptor activity | 0.019816 |
| GO:0030020 | molecular_function | extracellular matrix structural constituent conferring tensile strength | 0.019816 |
| GO:0000099 | molecular_function | sulfur amino acid transmembrane transporter activity | 0.019816 |
| GO:0046790 | molecular_function | virion binding | 0.019816 |
| GO:0005529 | molecular_function | sugar binding | 0.02173 |
| GO:0005515 | molecular_function | protein binding | 0.022546 |
| GO:0001848 | molecular_function | complement binding | 0.02381 |
| GO:0017025 | molecular_function | TATA-binding protein binding | 0.02381 |
| GO:0001968 | molecular_function | fibronectin binding | 0.02381 |
| GO:0047555 | molecular_function | 3',5'-cyclic-GMP phosphodiesterase activity | 0.02381 |
| GO:0017134 | molecular_function | fibroblast growth factor binding | 0.029171 |
| GO:0005518 | molecular_function | collagen binding | 0.029725 |
| GO:0016563 | molecular_function | transcription activator activity | 0.029838 |
| GO:0042813 | molecular_function | Wnt receptor activity | 0.033956 |
| GO:0048531 | molecular_function | beta-1,3-galactosyltransferase activity | 0.033956 |
| GO:0005243 | molecular_function | gap junction channel activity | 0.038753 |
| GO:0016861 | molecular_function | intramolecular oxidoreductase activity, interconverting aldoses and ketoses | 0.038753 |
| GO:0008191 | molecular_function | metalloendopeptidase inhibitor activity | 0.038753 |
| GO:0010576 | molecular_function | metalloenzyme regulator activity | 0.038753 |
| GO:0048551 | molecular_function | metalloenzyme inhibitor activity | 0.038753 |
| GO:0030553 | molecular_function | cGMP binding | 0.038753 |
| GO:0004497 | molecular_function | monooxygenase activity | 0.043663 |
| GO:0022829 | molecular_function | wide pore channel activity | 0.043663 |
| GO:0016986 | molecular_function | transcription initiation factor activity | 0.043663 |
| GO:0043236 | molecular_function | laminin binding | 0.043663 |
| GO:0004926 | molecular_function | non-G-protein coupled 7TM receptor activity | 0.043663 |
| GO:0019002 | molecular_function | GMP binding | 0.043663 |
| GO:0005313 | molecular_function | L-glutamate transmembrane transporter activity | 0.043663 |
| GO:0043394 | molecular_function | proteoglycan binding | 0.049032 |
| GO:0008536 | molecular_function | Ran GTPase binding | 0.049032 |
| GO:0017127 | molecular_function | cholesterol transporter activity | 0.049032 |
| GO:0015172 | molecular_function | acidic amino acid transmembrane transporter activity | 0.049032 |
| GO:0004303 | molecular_function | estradiol 17-beta-dehydrogenase activity | 0.049032 |
