# Supplementary material for: Genes and Gene Ontologies Common to Airflow Obstruction and Emphysema in the Lungs of Patients with COPD
Source: PLoS One. 2011 Mar 15;6(3):e17442. doi: 10.1371/journal.pone.0017442 (PMC3057973; doi:10.1371/journal.pone.0017442)
Supplement: Table S6 — Gene ontologies enriched in Bhattacharya et al FEV1 dataset. (DOCX) [file pone.0017442.s008.docx]

**Table S6: Gene ontologies enriched in Bhattacharya *et al* dataset differentiating normals (FEV_1_ >80% predicted) from COPD patients (FEV_1_ <70% predicted)**

| **GOID** | **Ontology** | **Term** | **p** |
| --- | --- | --- | --- |
| GO:0004957 | molecular_function | prostaglandin E receptor activity | 0.002955 |
| GO:0004415 | molecular_function | hyalurononglucosaminidase activity | 0.005186 |
| GO:0047372 | molecular_function | acylglycerol lipase activity | 0.005186 |
| GO:0030177 | biological_process | positive regulation of Wnt receptor signaling pathway | 0.013565 |
| GO:0004953 | molecular_function | icosanoid receptor activity | 0.015701 |
| GO:0004954 | molecular_function | prostanoid receptor activity | 0.015701 |
| GO:0004955 | molecular_function | prostaglandin receptor activity | 0.015701 |
| GO:0007567 | biological_process | parturition | 0.015701 |
| GO:0045786 | biological_process | negative regulation of cell cycle | 0.015701 |
| GO:0070403 | molecular_function | NAD binding | 0.015701 |
| GO:0007342 | biological_process | fusion of sperm to egg plasma membrane | 0.015701 |
| GO:0031519 | cellular_component | PcG protein complex | 0.015701 |
| GO:0045026 | biological_process | plasma membrane fusion | 0.017694 |
| GO:0015929 | molecular_function | hexosaminidase activity | 0.022892 |
| GO:0007271 | biological_process | synaptic transmission, cholinergic | 0.032644 |
| GO:0004889 | molecular_function | nicotinic acetylcholine-activated cation-selective channel activity | 0.03896 |
| GO:0005892 | cellular_component | nicotinic acetylcholine-gated receptor-channel complex | 0.03896 |
| GO:0007339 | biological_process | binding of sperm to zona pellucida | 0.03896 |
| GO:0035036 | biological_process | sperm-egg recognition | 0.03896 |
