# Supplementary material for: Genes and Gene Ontologies Common to Airflow Obstruction and Emphysema in the Lungs of Patients with COPD
Source: PLoS One. 2011 Mar 15;6(3):e17442. doi: 10.1371/journal.pone.0017442 (PMC3057973; doi:10.1371/journal.pone.0017442)
Supplement: Table S7 — Gene ontologies enriched in Spira et al DLCO dataset. (DOCX) [file pone.0017442.s009.docx]

**Table S7: Gene ontologies enriched in Spira *et al* dataset using DLCO to classify normal/mild and severe emphysema subjects**

| **GOID** | **Ontology** | **Term** | **p** |
| --- | --- | --- | --- |
| GO:0001516 | biological_process | prostaglandin biosynthetic process | 4.07E-07 |
| GO:0046457 | biological_process | prostanoid biosynthetic process | 4.07E-07 |
| GO:0006692 | biological_process | prostanoid metabolic process | 6.85E-06 |
| GO:0006693 | biological_process | prostaglandin metabolic process | 6.85E-06 |
| GO:0048583 | biological_process | regulation of response to stimulus | 1.28E-05 |
| GO:0030199 | biological_process | collagen fibril organization | 2.56E-05 |
| GO:0022410 | biological_process | circadian sleep/wake cycle process | 2.76E-05 |
| GO:0042749 | biological_process | regulation of circadian sleep/wake cycle | 2.76E-05 |
| GO:0045187 | biological_process | regulation of circadian sleep/wake cycle, sleep | 2.76E-05 |
| GO:0006958 | biological_process | complement activation, classical pathway | 3.58E-05 |
| GO:0042745 | biological_process | circadian sleep/wake cycle | 3.80E-05 |
| GO:0046456 | biological_process | icosanoid biosynthetic process | 4.06E-05 |
| GO:0002455 | biological_process | humoral immune response mediated by circulating immunoglobulin | 4.71E-05 |
| GO:0006636 | biological_process | unsaturated fatty acid biosynthetic process | 6.37E-05 |
| GO:0048512 | biological_process | circadian behavior | 6.86E-05 |
| GO:0007622 | biological_process | rhythmic behavior | 0.000119 |
| GO:0006956 | biological_process | complement activation | 0.000157 |
| GO:0002541 | biological_process | activation of plasma proteins involved in acute inflammatory response | 0.000175 |
| GO:0006959 | biological_process | humoral immune response | 0.000191 |
| GO:0030431 | biological_process | sleep | 0.00028 |
| GO:0042752 | biological_process | regulation of circadian rhythm | 0.000326 |
| GO:0016064 | biological_process | immunoglobulin mediated immune response | 0.000332 |
| GO:0006690 | biological_process | icosanoid metabolic process | 0.000332 |
| GO:0019724 | biological_process | B cell mediated immunity | 0.000362 |
| GO:0033559 | biological_process | unsaturated fatty acid metabolic process | 0.000422 |
| GO:0006030 | biological_process | chitin metabolic process | 0.000422 |
| GO:0006032 | biological_process | chitin catabolic process | 0.000422 |
| GO:0030198 | biological_process | extracellular matrix organization | 0.000477 |
| GO:0009987 | biological_process | cellular process | 0.000837 |
| GO:0042730 | biological_process | fibrinolysis | 0.000943 |
| GO:0002449 | biological_process | lymphocyte mediated immunity | 0.000996 |
| GO:0051604 | biological_process | protein maturation | 0.001471 |
| GO:0002250 | biological_process | adaptive immune response | 0.001591 |
| GO:0002460 | biological_process | adaptive immune response based on somatic recombination of immune receptors built from immunoglobulin superfamily domains | 0.001591 |
| GO:0002443 | biological_process | leukocyte mediated immunity | 0.001958 |
| GO:0006633 | biological_process | fatty acid biosynthetic process | 0.001958 |
| GO:0051605 | biological_process | protein maturation by peptide bond cleavage | 0.002904 |
| GO:0045087 | biological_process | innate immune response | 0.002904 |
| GO:0006458 | biological_process | 'de novo' protein folding | 0.003148 |
| GO:0006957 | biological_process | complement activation, alternative pathway | 0.003148 |
| GO:0007623 | biological_process | circadian rhythm | 0.003163 |
| GO:0002253 | biological_process | activation of immune response | 0.003271 |
| GO:0032101 | biological_process | regulation of response to external stimulus | 0.003467 |
| GO:0043062 | biological_process | extracellular structure organization | 0.003682 |
| GO:0002526 | biological_process | acute inflammatory response | 0.003709 |
| GO:0050795 | biological_process | regulation of behavior | 0.003914 |
| GO:0001501 | biological_process | skeletal system development | 0.004296 |
| GO:0030195 | biological_process | negative regulation of blood coagulation | 0.004676 |
| GO:0001503 | biological_process | ossification | 0.005397 |
| GO:0007517 | biological_process | muscle organ development | 0.005466 |
| GO:0009065 | biological_process | glutamine family amino acid catabolic process | 0.006287 |
| GO:0050819 | biological_process | negative regulation of coagulation | 0.006287 |
| GO:0016485 | biological_process | protein processing | 0.007198 |
| GO:0006026 | biological_process | aminoglycan catabolic process | 0.007198 |
| GO:0060348 | biological_process | bone development | 0.007221 |
| GO:0019752 | biological_process | carboxylic acid metabolic process | 0.007421 |
| GO:0043436 | biological_process | oxoacid metabolic process | 0.007421 |
| GO:0006082 | biological_process | organic acid metabolic process | 0.00777 |
| GO:0042180 | biological_process | cellular ketone metabolic process | 0.008495 |
| GO:0002252 | biological_process | immune effector process | 0.010013 |
| GO:0007155 | biological_process | cell adhesion | 0.010013 |
| GO:0022610 | biological_process | biological adhesion | 0.010013 |
| GO:0045988 | biological_process | negative regulation of striated muscle contraction | 0.010013 |
| GO:0010871 | biological_process | negative regulation of receptor biosynthetic process | 0.010013 |
| GO:0001957 | biological_process | intramembranous ossification | 0.010013 |
| GO:0022417 | biological_process | protein maturation by protein folding | 0.010013 |
| GO:0050435 | biological_process | beta-amyloid metabolic process | 0.010013 |
| GO:0042026 | biological_process | protein refolding | 0.010013 |
| GO:0060347 | biological_process | heart trabecula formation | 0.010013 |
| GO:0007589 | biological_process | body fluid secretion | 0.010021 |
| GO:0006631 | biological_process | fatty acid metabolic process | 0.010985 |
| GO:0009063 | biological_process | cellular amino acid catabolic process | 0.011081 |
| GO:0000272 | biological_process | polysaccharide catabolic process | 0.012341 |
| GO:0050778 | biological_process | positive regulation of immune response | 0.013442 |
| GO:0032787 | biological_process | monocarboxylic acid metabolic process | 0.013442 |
| GO:0055078 | biological_process | sodium ion homeostasis | 0.013442 |
| GO:0050727 | biological_process | regulation of inflammatory response | 0.013442 |
| GO:0050729 | biological_process | positive regulation of inflammatory response | 0.013442 |
| GO:0051153 | biological_process | regulation of striated muscle cell differentiation | 0.013442 |
| GO:0043277 | biological_process | apoptotic cell clearance | 0.013442 |
| GO:0050748 | biological_process | negative regulation of lipoprotein metabolic process | 0.013442 |
| GO:0032268 | biological_process | regulation of cellular protein metabolic process | 0.01347 |
| GO:0030193 | biological_process | regulation of blood coagulation | 0.013819 |
| GO:0050776 | biological_process | regulation of immune response | 0.01456 |
| GO:0006417 | biological_process | regulation of translation | 0.014575 |
| GO:0051239 | biological_process | regulation of multicellular organismal process | 0.016429 |
| GO:0009310 | biological_process | amine catabolic process | 0.016492 |
| GO:0001568 | biological_process | blood vessel development | 0.016492 |
| GO:0048584 | biological_process | positive regulation of response to stimulus | 0.016711 |
| GO:0002029 | biological_process | desensitization of G-protein coupled receptor protein signaling pathway | 0.016711 |
| GO:0022401 | biological_process | adaptation of signaling pathway | 0.016711 |
| GO:0033605 | biological_process | positive regulation of catecholamine secretion | 0.016711 |
| GO:0002861 | biological_process | regulation of inflammatory response to antigenic stimulus | 0.016711 |
| GO:0032092 | biological_process | positive regulation of protein binding | 0.016711 |
| GO:0043206 | biological_process | fibril organization | 0.016711 |
| GO:0060343 | biological_process | trabecula formation | 0.016711 |
| GO:0001944 | biological_process | vasculature development | 0.017564 |
| GO:0017148 | biological_process | negative regulation of translation | 0.017864 |
| GO:0050818 | biological_process | regulation of coagulation | 0.017864 |
| GO:0016053 | biological_process | organic acid biosynthetic process | 0.019569 |
| GO:0046394 | biological_process | carboxylic acid biosynthetic process | 0.019569 |
| GO:0043567 | biological_process | regulation of insulin-like growth factor receptor signaling pathway | 0.02036 |
| GO:0007217 | biological_process | tachykinin receptor signaling pathway | 0.02036 |
| GO:0006527 | biological_process | arginine catabolic process | 0.02036 |
| GO:0051149 | biological_process | positive regulation of muscle cell differentiation | 0.02036 |
| GO:0050746 | biological_process | regulation of lipoprotein metabolic process | 0.02036 |
| GO:0007183 | biological_process | SMAD protein complex assembly | 0.02036 |
| GO:0060314 | biological_process | regulation of ryanodine-sensitive calcium-release channel activity | 0.02036 |
| GO:0044238 | biological_process | primary metabolic process | 0.020886 |
| GO:0051591 | biological_process | response to cAMP | 0.022925 |
| GO:0010033 | biological_process | response to organic substance | 0.024089 |
| GO:0006911 | biological_process | phagocytosis, engulfment | 0.024089 |
| GO:0006548 | biological_process | histidine catabolic process | 0.024089 |
| GO:0009077 | biological_process | histidine family amino acid catabolic process | 0.024089 |
| GO:0006809 | biological_process | nitric oxide biosynthetic process | 0.024089 |
| GO:0010869 | biological_process | regulation of receptor biosynthetic process | 0.024089 |
| GO:0002714 | biological_process | positive regulation of B cell mediated immunity | 0.024089 |
| GO:0002886 | biological_process | regulation of myeloid leukocyte mediated immunity | 0.024089 |
| GO:0002891 | biological_process | positive regulation of immunoglobulin mediated immune response | 0.024089 |
| GO:0042987 | biological_process | amyloid precursor protein catabolic process | 0.024089 |
| GO:0048660 | biological_process | regulation of smooth muscle cell proliferation | 0.024838 |
| GO:0051789 | biological_process | response to protein stimulus | 0.025145 |
| GO:0044270 | biological_process | cellular nitrogen compound catabolic process | 0.027034 |
| GO:0051085 | biological_process | chaperone mediated protein folding requiring cofactor | 0.028699 |
| GO:0046209 | biological_process | nitric oxide metabolic process | 0.028699 |
| GO:0032925 | biological_process | regulation of activin receptor signaling pathway | 0.028699 |
| GO:0080134 | biological_process | regulation of response to stress | 0.029298 |
| GO:0048731 | biological_process | system development | 0.031257 |
| GO:0006909 | biological_process | phagocytosis | 0.031257 |
| GO:0016044 | biological_process | membrane organization | 0.031464 |
| GO:0051246 | biological_process | regulation of protein metabolic process | 0.032574 |
| GO:0007213 | biological_process | muscarinic acetylcholine receptor signaling pathway | 0.032915 |
| GO:0045932 | biological_process | negative regulation of muscle contraction | 0.032915 |
| GO:0002675 | biological_process | positive regulation of acute inflammatory response | 0.032915 |
| GO:0006183 | biological_process | GTP biosynthetic process | 0.032915 |
| GO:0006228 | biological_process | UTP biosynthetic process | 0.032915 |
| GO:0046051 | biological_process | UTP metabolic process | 0.032915 |
| GO:0042982 | biological_process | amyloid precursor protein metabolic process | 0.032915 |
| GO:0014910 | biological_process | regulation of smooth muscle cell migration | 0.036832 |
| GO:0051084 | biological_process | 'de novo' posttranslational protein folding | 0.036832 |
| GO:0051954 | biological_process | positive regulation of amine transport | 0.036832 |
| GO:0006547 | biological_process | histidine metabolic process | 0.036832 |
| GO:0009075 | biological_process | histidine family amino acid metabolic process | 0.036832 |
| GO:0010745 | biological_process | negative regulation of macrophage derived foam cell differentiation | 0.036832 |
| GO:0010888 | biological_process | negative regulation of lipid storage | 0.036832 |
| GO:0045453 | biological_process | bone resorption | 0.036832 |
| GO:0006613 | biological_process | cotranslational protein targeting to membrane | 0.036832 |
| GO:0050766 | biological_process | positive regulation of phagocytosis | 0.036832 |
| GO:0009208 | biological_process | pyrimidine ribonucleoside triphosphate metabolic process | 0.036832 |
| GO:0009209 | biological_process | pyrimidine ribonucleoside triphosphate biosynthetic process | 0.036832 |
| GO:0006241 | biological_process | CTP biosynthetic process | 0.036832 |
| GO:0046036 | biological_process | CTP metabolic process | 0.036832 |
| GO:0043666 | biological_process | regulation of phosphoprotein phosphatase activity | 0.036832 |
| GO:0032269 | biological_process | negative regulation of cellular protein metabolic process | 0.037154 |
| GO:0016054 | biological_process | organic acid catabolic process | 0.038199 |
| GO:0046395 | biological_process | carboxylic acid catabolic process | 0.038199 |
| GO:0048514 | biological_process | blood vessel morphogenesis | 0.038298 |
| GO:0008277 | biological_process | regulation of G-protein coupled receptor protein signaling pathway | 0.039574 |
| GO:0048771 | biological_process | tissue remodeling | 0.039574 |
| GO:0009308 | biological_process | amine metabolic process | 0.041344 |
| GO:0048662 | biological_process | negative regulation of smooth muscle cell proliferation | 0.041344 |
| GO:0045822 | biological_process | negative regulation of heart contraction | 0.041344 |
| GO:0009064 | biological_process | glutamine family amino acid metabolic process | 0.041344 |
| GO:0051482 | biological_process | elevation of cytosolic calcium ion concentration during G-protein signaling, coupled to IP3 second messenger (phospholipase C activating) | 0.041344 |
| GO:0006525 | biological_process | arginine metabolic process | 0.041344 |
| GO:0042375 | biological_process | quinone cofactor metabolic process | 0.041344 |
| GO:0009148 | biological_process | pyrimidine nucleoside triphosphate biosynthetic process | 0.041344 |
| GO:0045744 | biological_process | negative regulation of G-protein coupled receptor protein signaling pathway | 0.047137 |
| GO:0007263 | biological_process | nitric oxide mediated signal transduction | 0.047137 |
| GO:0046039 | biological_process | GTP metabolic process | 0.047137 |
| GO:0006892 | biological_process | post-Golgi vesicle-mediated transport | 0.049262 |
| GO:0006897 | biological_process | endocytosis | 0.051235 |
| GO:0010324 | biological_process | membrane invagination | 0.051235 |
| GO:0042542 | biological_process | response to hydrogen peroxide | 0.051357 |
| GO:0051248 | biological_process | negative regulation of protein metabolic process | 0.051804 |
| GO:0008152 | biological_process | metabolic process | 0.052001 |
| GO:0032369 | biological_process | negative regulation of lipid transport | 0.052336 |
| GO:0031398 | biological_process | positive regulation of protein ubiquitination | 0.052336 |
| GO:0048856 | biological_process | anatomical structure development | 0.054049 |
| GO:0051592 | biological_process | response to calcium ion | 0.055186 |
| GO:0048511 | biological_process | rhythmic process | 0.055186 |
| GO:0006986 | biological_process | response to unfolded protein | 0.055186 |
| GO:0048519 | biological_process | negative regulation of biological process | 0.056787 |
| GO:0010608 | biological_process | posttranscriptional regulation of gene expression | 0.057199 |
| GO:0002026 | biological_process | regulation of the force of heart contraction | 0.057789 |
| GO:0050764 | biological_process | regulation of phagocytosis | 0.057789 |
| GO:0009147 | biological_process | pyrimidine nucleoside triphosphate metabolic process | 0.057789 |
| GO:0001525 | biological_process | angiogenesis | 0.058853 |
| GO:0001502 | biological_process | cartilage condensation | 0.063167 |
| GO:0008610 | biological_process | lipid biosynthetic process | 0.063167 |
| GO:0034199 | biological_process | activation of protein kinase A activity | 0.063167 |
| GO:0009220 | biological_process | pyrimidine ribonucleotide biosynthetic process | 0.063167 |
| GO:0010921 | biological_process | regulation of phosphatase activity | 0.063167 |
| GO:0051004 | biological_process | regulation of lipoprotein lipase activity | 0.063167 |
| GO:0006516 | biological_process | glycoprotein catabolic process | 0.063167 |
| GO:0016192 | biological_process | vesicle-mediated transport | 0.063171 |
| GO:0032103 | biological_process | positive regulation of response to external stimulus | 0.065939 |
| GO:0009968 | biological_process | negative regulation of signal transduction | 0.066195 |
| GO:0006810 | biological_process | transport | 0.06747 |
| GO:0018107 | biological_process | peptidyl-threonine phosphorylation | 0.06825 |
| GO:0050433 | biological_process | regulation of catecholamine secretion | 0.06825 |
| GO:0060048 | biological_process | cardiac muscle contraction | 0.06825 |
| GO:0006541 | biological_process | glutamine metabolic process | 0.06825 |
| GO:0006022 | biological_process | aminoglycan metabolic process | 0.06825 |
| GO:0018205 | biological_process | peptidyl-lysine modification | 0.06825 |
| GO:0045445 | biological_process | myoblast differentiation | 0.06825 |
| GO:0046849 | biological_process | bone remodeling | 0.06825 |
| GO:0009218 | biological_process | pyrimidine ribonucleotide metabolic process | 0.06825 |
| GO:0031347 | biological_process | regulation of defense response | 0.072162 |
| GO:0002684 | biological_process | positive regulation of immune system process | 0.073182 |
| GO:0051234 | biological_process | establishment of localization | 0.073237 |
| GO:0019835 | biological_process | cytolysis | 0.07469 |
| GO:0030968 | biological_process | endoplasmic reticulum unfolded protein response | 0.07469 |
| GO:0034620 | biological_process | cellular response to unfolded protein | 0.07469 |
| GO:0046131 | biological_process | pyrimidine ribonucleoside metabolic process | 0.07469 |
| GO:0043462 | biological_process | regulation of ATPase activity | 0.080671 |
| GO:0001974 | biological_process | blood vessel remodeling | 0.080671 |
| GO:0030574 | biological_process | collagen catabolic process | 0.080671 |
| GO:0055002 | biological_process | striated muscle cell development | 0.080671 |
| GO:0002673 | biological_process | regulation of acute inflammatory response | 0.080671 |
| GO:0042036 | biological_process | negative regulation of cytokine biosynthetic process | 0.080671 |
| GO:0031349 | biological_process | positive regulation of defense response | 0.082257 |
| GO:0018210 | biological_process | peptidyl-threonine modification | 0.087157 |
| GO:0030004 | biological_process | cellular monovalent inorganic cation homeostasis | 0.087157 |
| GO:0010883 | biological_process | regulation of lipid storage | 0.087157 |
| GO:0003208 | biological_process | cardiac ventricle morphogenesis | 0.087157 |
| GO:0003229 | biological_process | ventricular cardiac muscle tissue development | 0.087157 |
| GO:0055010 | biological_process | ventricular cardiac muscle tissue morphogenesis | 0.087157 |
| GO:0051241 | biological_process | negative regulation of multicellular organismal process | 0.089331 |
| GO:0014706 | biological_process | striated muscle tissue development | 0.091054 |
| GO:0003015 | biological_process | heart process | 0.0933 |
| GO:0060047 | biological_process | heart contraction | 0.0933 |
| GO:0002712 | biological_process | regulation of B cell mediated immunity | 0.0933 |
| GO:0002889 | biological_process | regulation of immunoglobulin mediated immune response | 0.0933 |
| GO:0003231 | biological_process | cardiac ventricle development | 0.0933 |
| GO:0010648 | biological_process | negative regulation of cell communication | 0.094455 |
| GO:0030003 | biological_process | cellular cation homeostasis | 0.094455 |
| GO:0000302 | biological_process | response to reactive oxygen species | 0.094919 |
| GO:0051384 | biological_process | response to glucocorticoid stimulus | 0.094919 |
| GO:0006942 | biological_process | regulation of striated muscle contraction | 0.099502 |
| GO:0050810 | biological_process | regulation of steroid biosynthetic process | 0.099502 |
| GO:0016525 | biological_process | negative regulation of angiogenesis | 0.099502 |
| GO:0005576 | cellular_component | extracellular region | 3.80E-09 |
| GO:0005578 | cellular_component | proteinaceous extracellular matrix | 2.57E-08 |
| GO:0031012 | cellular_component | extracellular matrix | 6.49E-08 |
| GO:0044421 | cellular_component | extracellular region part | 1.39E-07 |
| GO:0044420 | cellular_component | extracellular matrix part | 7.31E-06 |
| GO:0042470 | cellular_component | melanosome | 2.72E-05 |
| GO:0048770 | cellular_component | pigment granule | 2.72E-05 |
| GO:0005791 | cellular_component | rough endoplasmic reticulum | 0.00028 |
| GO:0030934 | cellular_component | anchoring collagen | 0.000943 |
| GO:0005581 | cellular_component | collagen | 0.00188 |
| GO:0005615 | cellular_component | extracellular space | 0.00301 |
| GO:0005783 | cellular_component | endoplasmic reticulum | 0.004297 |
| GO:0016023 | cellular_component | cytoplasmic membrane-bounded vesicle | 0.007221 |
| GO:0031988 | cellular_component | membrane-bounded vesicle | 0.008185 |
| GO:0042383 | cellular_component | sarcolemma | 0.008496 |
| GO:0044444 | cellular_component | cytoplasmic part | 0.010013 |
| GO:0017059 | cellular_component | serine C-palmitoyltransferase complex | 0.010013 |
| GO:0031211 | cellular_component | palmitoyltransferase complex | 0.010013 |
| GO:0001527 | cellular_component | microfibril | 0.010013 |
| GO:0005604 | cellular_component | basement membrane | 0.012949 |
| GO:0005593 | cellular_component | FACIT collagen | 0.013442 |
| GO:0001931 | cellular_component | uropod | 0.013442 |
| GO:0031254 | cellular_component | trailing edge | 0.013442 |
| GO:0031410 | cellular_component | cytoplasmic vesicle | 0.016429 |
| GO:0005788 | cellular_component | endoplasmic reticulum lumen | 0.016711 |
| GO:0031965 | cellular_component | nuclear membrane | 0.016711 |
| GO:0005579 | cellular_component | membrane attack complex | 0.016711 |
| GO:0005885 | cellular_component | Arp2/3 protein complex | 0.016711 |
| GO:0005771 | cellular_component | multivesicular body | 0.016711 |
| GO:0031982 | cellular_component | vesicle | 0.020317 |
| GO:0043205 | cellular_component | fibril | 0.02036 |
| GO:0044432 | cellular_component | endoplasmic reticulum part | 0.023378 |
| GO:0031093 | cellular_component | platelet alpha granule lumen | 0.02825 |
| GO:0005952 | cellular_component | cAMP-dependent protein kinase complex | 0.028699 |
| GO:0005737 | cellular_component | cytoplasm | 0.032147 |
| GO:0060205 | cellular_component | cytoplasmic membrane-bounded vesicle lumen | 0.032741 |
| GO:0005583 | cellular_component | fibrillar collagen | 0.032915 |
| GO:0031983 | cellular_component | vesicle lumen | 0.035899 |
| GO:0005744 | cellular_component | mitochondrial inner membrane presequence translocase complex | 0.036832 |
| GO:0043198 | cellular_component | dendritic shaft | 0.041344 |
| GO:0048471 | cellular_component | perinuclear region of cytoplasm | 0.044724 |
| GO:0005625 | cellular_component | soluble fraction | 0.049262 |
| GO:0031091 | cellular_component | platelet alpha granule | 0.049262 |
| GO:0045263 | cellular_component | proton-transporting ATP synthase complex, coupling factor F(o) | 0.052336 |
| GO:0005865 | cellular_component | striated muscle thin filament | 0.06825 |
| GO:0005753 | cellular_component | mitochondrial proton-transporting ATP synthase complex | 0.080671 |
| GO:0045259 | cellular_component | proton-transporting ATP synthase complex | 0.0933 |
| GO:0031984 | cellular_component | organelle subcompartment | 0.099502 |
| GO:0016860 | molecular_function | intramolecular oxidoreductase activity | 6.85E-06 |
| GO:0016853 | molecular_function | isomerase activity | 2.76E-05 |
| GO:0005488 | molecular_function | binding | 3.38E-05 |
| GO:0005509 | molecular_function | calcium ion binding | 6.86E-05 |
| GO:0005201 | molecular_function | extracellular matrix structural constituent | 0.00028 |
| GO:0005501 | molecular_function | retinoid binding | 0.000326 |
| GO:0019840 | molecular_function | isoprenoid binding | 0.000422 |
| GO:0004568 | molecular_function | chitinase activity | 0.000422 |
| GO:0005546 | molecular_function | phosphatidylinositol-4,5-bisphosphate binding | 0.001303 |
| GO:0005515 | molecular_function | protein binding | 0.00167 |
| GO:0005518 | molecular_function | collagen binding | 0.00188 |
| GO:0004859 | molecular_function | phospholipase inhibitor activity | 0.002093 |
| GO:0055102 | molecular_function | lipase inhibitor activity | 0.003682 |
| GO:0008289 | molecular_function | lipid binding | 0.007527 |
| GO:0005520 | molecular_function | insulin-like growth factor binding | 0.010013 |
| GO:0004758 | molecular_function | serine C-palmitoyltransferase activity | 0.010013 |
| GO:0016454 | molecular_function | C-palmitoyltransferase activity | 0.010013 |
| GO:0004857 | molecular_function | enzyme inhibitor activity | 0.010013 |
| GO:0008157 | molecular_function | protein phosphatase 1 binding | 0.010013 |
| GO:0004500 | molecular_function | dopamine beta-monooxygenase activity | 0.010013 |
| GO:0005218 | molecular_function | intracellular ligand-gated calcium channel activity | 0.010013 |
| GO:0005219 | molecular_function | ryanodine-sensitive calcium-release channel activity | 0.010013 |
| GO:0005544 | molecular_function | calcium-dependent phospholipid binding | 0.010021 |
| GO:0031994 | molecular_function | insulin-like growth factor I binding | 0.013442 |
| GO:0016715 | molecular_function | oxidoreductase activity, acting on paired donors, with incorporation or reduction of molecular oxygen, reduced ascorbate as one donor, and incorporation of one atom of oxygen | 0.013442 |
| GO:0017091 | molecular_function | AU-rich element binding | 0.013442 |
| GO:0017137 | molecular_function | Rab GTPase binding | 0.013819 |
| GO:0019798 | molecular_function | procollagen-proline dioxygenase activity | 0.016711 |
| GO:0004703 | molecular_function | G-protein coupled receptor kinase activity | 0.016711 |
| GO:0005198 | molecular_function | structural molecule activity | 0.017286 |
| GO:0003755 | molecular_function | peptidyl-prolyl cis-trans isomerase activity | 0.019392 |
| GO:0031543 | molecular_function | peptidyl-proline dioxygenase activity | 0.02036 |
| GO:0008143 | molecular_function | poly(A) RNA binding | 0.02036 |
| GO:0005527 | molecular_function | macrolide binding | 0.02036 |
| GO:0005528 | molecular_function | FK506 binding | 0.02036 |
| GO:0016859 | molecular_function | cis-trans isomerase activity | 0.021268 |
| GO:0003756 | molecular_function | protein disulfide isomerase activity | 0.024089 |
| GO:0016864 | molecular_function | intramolecular oxidoreductase activity, transposing S-S bonds | 0.024089 |
| GO:0019864 | molecular_function | IgG binding | 0.024089 |
| GO:0003810 | molecular_function | protein-glutamine gamma-glutamyltransferase activity | 0.024089 |
| GO:0070717 | molecular_function | poly-purine tract binding | 0.024089 |
| GO:0015278 | molecular_function | calcium-release channel activity | 0.024089 |
| GO:0004497 | molecular_function | monooxygenase activity | 0.026078 |
| GO:0016862 | molecular_function | intramolecular oxidoreductase activity, interconverting keto- and enol-groups | 0.028699 |
| GO:0034713 | molecular_function | type I transforming growth factor beta receptor binding | 0.028699 |
| GO:0048185 | molecular_function | activin binding | 0.028699 |
| GO:0035091 | molecular_function | phosphoinositide binding | 0.032292 |
| GO:0004550 | molecular_function | nucleoside diphosphate kinase activity | 0.032915 |
| GO:0015450 | molecular_function | P-P-bond-hydrolysis-driven protein transmembrane transporter activity | 0.036832 |
| GO:0022884 | molecular_function | macromolecule transmembrane transporter activity | 0.036832 |
| GO:0016813 | molecular_function | hydrolase activity, acting on carbon-nitrogen (but not peptide) bonds, in linear amidines | 0.036832 |
| GO:0008092 | molecular_function | cytoskeletal protein binding | 0.041344 |
| GO:0016409 | molecular_function | palmitoyltransferase activity | 0.047137 |
| GO:0030552 | molecular_function | cAMP binding | 0.047137 |
| GO:0005217 | molecular_function | intracellular ligand-gated ion channel activity | 0.052336 |
| GO:0016408 | molecular_function | C-acyltransferase activity | 0.057789 |
| GO:0008603 | molecular_function | cAMP-dependent protein kinase regulator activity | 0.057789 |
| GO:0019865 | molecular_function | immunoglobulin binding | 0.063167 |
| GO:0008320 | molecular_function | protein transmembrane transporter activity | 0.063167 |
| GO:0004675 | molecular_function | transmembrane receptor protein serine/threonine kinase activity | 0.063167 |
| GO:0005024 | molecular_function | transforming growth factor beta receptor activity | 0.063167 |
| GO:0005507 | molecular_function | copper ion binding | 0.080671 |
| GO:0035250 | molecular_function | UDP-galactosyltransferase activity | 0.080671 |
| GO:0005160 | molecular_function | transforming growth factor beta receptor binding | 0.080671 |
| GO:0016755 | molecular_function | transferase activity, transferring amino-acyl groups | 0.087157 |
| GO:0004866 | molecular_function | endopeptidase inhibitor activity | 0.091054 |
| GO:0003746 | molecular_function | translation elongation factor activity | 0.0933 |
| GO:0016208 | molecular_function | AMP binding | 0.099502 |
| GO:0030551 | molecular_function | cyclic nucleotide binding | 0.099502 |
