# Supplementary material for: Genes and Gene Ontologies Common to Airflow Obstruction and Emphysema in the Lungs of Patients with COPD
Source: PLoS One. 2011 Mar 15;6(3):e17442. doi: 10.1371/journal.pone.0017442 (PMC3057973; doi:10.1371/journal.pone.0017442)
Supplement: Table S8 — Gene ontologies enriched in Golpon et al DLCO dataset. (DOCX) [file pone.0017442.s010.docx]

**Table S8: Gene ontologies enriched in Golpon *et al* dataset using DLCO to classify normal and severe emphysema subjects**

| **GOID** | **Ontology** | **Term** | **p** |
| --- | --- | --- | --- |
| GO:0009653 | biological_process | anatomical structure morphogenesis | 0.016016 |
| GO:0010926 | biological_process | anatomical structure formation | 0.002609 |
| GO:0048646 | biological_process | anatomical structure formation involved in morphogenesis | 0.021632 |
| GO:0048731 | biological_process | system development | 0.074319 |
| GO:0048856 | biological_process | anatomical structure development | 0.062016 |
| GO:0001701 | biological_process | in utero embryonic development | 0.052106 |
| GO:0008152 | biological_process | metabolic process | 0.017833 |
| GO:0009987 | biological_process | cellular process | 0.001856 |
| GO:0044237 | biological_process | cellular metabolic process | 0.017311 |
| GO:0044238 | biological_process | primary metabolic process | 0.052106 |
| GO:0007162 | biological_process | negative regulation of cell adhesion | 0.015705 |
| GO:0048519 | biological_process | negative regulation of biological process | 0.049806 |
| GO:0048523 | biological_process | negative regulation of cellular process | 0.058786 |
| GO:0007178 | biological_process | transmembrane receptor protein serine/threonine kinase signaling pathway | 0.087413 |
| GO:0007179 | biological_process | transforming growth factor beta receptor signaling pathway | 0.027958 |
| GO:0003013 | biological_process | circulatory system process | 0.070947 |
| GO:0008015 | biological_process | blood circulation | 0.070947 |
| GO:0008217 | biological_process | regulation of blood pressure | 0.087413 |
| GO:0065008 | biological_process | regulation of biological quality | 0.057811 |
| GO:0010594 | biological_process | regulation of endothelial cell migration | 0.078415 |
| GO:0010596 | biological_process | negative regulation of endothelial cell migration | 0.032279 |
| GO:0030334 | biological_process | regulation of cell migration | 0.05726 |
| GO:0030336 | biological_process | negative regulation of cell migration | 0.029127 |
| GO:0032879 | biological_process | regulation of localization | 0.029556 |
| GO:0040012 | biological_process | regulation of locomotion | 0.07666 |
| GO:0051270 | biological_process | regulation of cellular component movement | 0.078038 |
| GO:0051271 | biological_process | negative regulation of cellular component movement | 0.034428 |
| GO:0032535 | biological_process | regulation of cellular component size | 0.04061 |
| GO:0090066 | biological_process | regulation of anatomical structure size | 0.017833 |
| GO:0030510 | biological_process | regulation of BMP signaling pathway | 0.08335 |
| GO:0030513 | biological_process | positive regulation of BMP signaling pathway | 0.00803 |
| GO:0048518 | biological_process | positive regulation of biological process | 0.005745 |
| GO:0048522 | biological_process | positive regulation of cellular process | 0.012776 |
| GO:0006928 | biological_process | cellular component movement | 0.009179 |
| GO:0016477 | biological_process | cell migration | 0.046708 |
| GO:0035313 | biological_process | wound healing, spreading of epidermal cells | 0.015378 |
| GO:0040011 | biological_process | locomotion | 0.064224 |
| GO:0048870 | biological_process | cell motility | 0.066068 |
| GO:0051179 | biological_process | localization | 0.092768 |
| GO:0051674 | biological_process | localization of cell | 0.066068 |
| GO:0001952 | biological_process | regulation of cell-matrix adhesion | 0.007664 |
| GO:0001953 | biological_process | negative regulation of cell-matrix adhesion | 0.001332 |
| GO:0010810 | biological_process | regulation of cell-substrate adhesion | 0.017833 |
| GO:0010812 | biological_process | negative regulation of cell-substrate adhesion | 0.001787 |
| GO:0044087 | biological_process | regulation of cellular component biogenesis | 0.033422 |
| GO:0051128 | biological_process | regulation of cellular component organization | 0.009131 |
| GO:0051129 | biological_process | negative regulation of cellular component organization | 0.002011 |
| GO:0051893 | biological_process | regulation of focal adhesion formation | 0.000919 |
| GO:0051895 | biological_process | negative regulation of focal adhesion formation | 0.000177 |
| GO:0001990 | biological_process | regulation of systemic arterial blood pressure by hormone | 0.052106 |
| GO:0002016 | biological_process | regulation of blood volume by renin-angiotensin | 0.015378 |
| GO:0003014 | biological_process | renal system process | 0.071082 |
| GO:0003044 | biological_process | regulation of systemic arterial blood pressure mediated by a chemical signal | 0.074319 |
| GO:0003081 | biological_process | regulation of systemic arterial blood pressure by renin-angiotensin | 0.023335 |
| GO:0050886 | biological_process | endocrine process | 0.052106 |
| GO:0003071 | biological_process | renal system process involved in regulation of systemic arterial blood pressure | 0.012776 |
| GO:0003078 | biological_process | regulation of natriuresis | 0.015378 |
| GO:0044062 | biological_process | regulation of excretion | 0.017833 |
| GO:0051049 | biological_process | regulation of transport | 0.070726 |
| GO:0010743 | biological_process | regulation of macrophage derived foam cell differentiation | 0.071082 |
| GO:0010744 | biological_process | positive regulation of macrophage derived foam cell differentiation | 0.029127 |
| GO:0010872 | biological_process | regulation of cholesterol esterification | 0.015378 |
| GO:0010873 | biological_process | positive regulation of cholesterol esterification | 0.012776 |
| GO:0045940 | biological_process | positive regulation of steroid metabolic process | 0.032279 |
| GO:0032268 | biological_process | regulation of cellular protein metabolic process | 0.057712 |
| GO:0010517 | biological_process | regulation of phospholipase activity | 0.00803 |
| GO:0010518 | biological_process | positive regulation of phospholipase activity | 0.00764 |
| GO:0032429 | biological_process | regulation of phospholipase A2 activity | 0.00803 |
| GO:0032430 | biological_process | positive regulation of phospholipase A2 activity | 0.00803 |
| GO:0051345 | biological_process | positive regulation of hydrolase activity | 0.070947 |
| GO:0060191 | biological_process | regulation of lipase activity | 0.012966 |
| GO:0060193 | biological_process | positive regulation of lipase activity | 0.009079 |
| GO:0051353 | biological_process | positive regulation of oxidoreductase activity | 0.074319 |
| GO:0016043 | biological_process | cellular component organization | 0.018462 |
| GO:0034367 | biological_process | macromolecular complex remodeling | 0.052106 |
| GO:0034368 | biological_process | protein-lipid complex remodeling | 0.052106 |
| GO:0034369 | biological_process | plasma lipoprotein particle remodeling | 0.052106 |
| GO:0034374 | biological_process | low-density lipoprotein particle remodeling | 0.017833 |
| GO:0043933 | biological_process | macromolecular complex subunit organization | 0.023215 |
| GO:0042312 | biological_process | regulation of vasodilation | 0.048753 |
| GO:0032101 | biological_process | regulation of response to external stimulus | 0.044302 |
| GO:0032103 | biological_process | positive regulation of response to external stimulus | 0.005745 |
| GO:0050729 | biological_process | positive regulation of inflammatory response | 0.087308 |
| GO:0007200 | biological_process | activation of phospholipase C activity by G-protein coupled receptor protein signaling pathway coupled to IP3 second messenger | 0.026012 |
| GO:0007202 | biological_process | activation of phospholipase C activity | 0.006083 |
| GO:0010863 | biological_process | positive regulation of phospholipase C activity | 0.006083 |
| GO:0048015 | biological_process | phosphoinositide-mediated signaling | 0.07426 |
| GO:0051482 | biological_process | elevation of cytosolic calcium ion concentration during G-protein signaling, coupled to IP3 second messenger (phospholipase C activating) | 0.029127 |
| GO:0000902 | biological_process | cell morphogenesis | 0.07207 |
| GO:0007409 | biological_process | axonogenesis | 0.068794 |
| GO:0007411 | biological_process | axon guidance | 0.080485 |
| GO:0008045 | biological_process | motor axon guidance | 0.023335 |
| GO:0030030 | biological_process | cell projection organization | 0.010223 |
| GO:0031175 | biological_process | neuron projection development | 0.036155 |
| GO:0032989 | biological_process | cellular component morphogenesis | 0.032279 |
| GO:0048666 | biological_process | neuron development | 0.080916 |
| GO:0048667 | biological_process | cell morphogenesis involved in neuron differentiation | 0.087308 |
| GO:0048812 | biological_process | neuron projection morphogenesis | 0.08504 |
| GO:0048858 | biological_process | cell projection morphogenesis | 0.092929 |
| GO:0006082 | biological_process | organic acid metabolic process | 0.06789 |
| GO:0006519 | biological_process | cellular amino acid and derivative metabolic process | 0.03646 |
| GO:0006520 | biological_process | cellular amino acid metabolic process | 0.009079 |
| GO:0006560 | biological_process | proline metabolic process | 0.017833 |
| GO:0006561 | biological_process | proline biosynthetic process | 0.010223 |
| GO:0006807 | biological_process | nitrogen compound metabolic process | 0.012515 |
| GO:0008652 | biological_process | cellular amino acid biosynthetic process | 0.025012 |
| GO:0009058 | biological_process | biosynthetic process | 0.040938 |
| GO:0009064 | biological_process | glutamine family amino acid metabolic process | 0.003922 |
| GO:0009084 | biological_process | glutamine family amino acid biosynthetic process | 0.045401 |
| GO:0009308 | biological_process | amine metabolic process | 0.055898 |
| GO:0009309 | biological_process | amine biosynthetic process | 0.062273 |
| GO:0019752 | biological_process | carboxylic acid metabolic process | 0.066813 |
| GO:0034641 | biological_process | cellular nitrogen compound metabolic process | 0.019955 |
| GO:0042180 | biological_process | cellular ketone metabolic process | 0.071082 |
| GO:0043436 | biological_process | oxoacid metabolic process | 0.066813 |
| GO:0044106 | biological_process | cellular amine metabolic process | 0.023335 |
| GO:0044249 | biological_process | cellular biosynthetic process | 0.030156 |
| GO:0044271 | biological_process | cellular nitrogen compound biosynthetic process | 0.050581 |
| GO:0009056 | biological_process | catabolic process | 0.037608 |
| GO:0009063 | biological_process | cellular amino acid catabolic process | 0.04508 |
| GO:0009065 | biological_process | glutamine family amino acid catabolic process | 0.004811 |
| GO:0009310 | biological_process | amine catabolic process | 0.058786 |
| GO:0044270 | biological_process | cellular nitrogen compound catabolic process | 0.086576 |
| GO:0006105 | biological_process | succinate metabolic process | 0.017833 |
| GO:0043648 | biological_process | dicarboxylic acid metabolic process | 0.001974 |
| GO:0006536 | biological_process | glutamate metabolic process | 0.002741 |
| GO:0006541 | biological_process | glutamine metabolic process | 0.045401 |
| GO:0006644 | biological_process | phospholipid metabolic process | 0.02099 |
| GO:0006650 | biological_process | glycerophospholipid metabolic process | 0.026012 |
| GO:0019637 | biological_process | organophosphate metabolic process | 0.023805 |
| GO:0046486 | biological_process | glycerolipid metabolic process | 0.051605 |
| GO:0006664 | biological_process | glycolipid metabolic process | 0.087308 |
| GO:0006677 | biological_process | glycosylceramide metabolic process | 0.012776 |
| GO:0006687 | biological_process | glycosphingolipid metabolic process | 0.067768 |
| GO:0006749 | biological_process | glutathione metabolic process | 0.074319 |
| GO:0042219 | biological_process | cellular amino acid derivative catabolic process | 0.048753 |
| GO:0045333 | biological_process | cellular respiration | 0.089578 |
| GO:0042133 | biological_process | neurotransmitter metabolic process | 0.055898 |
| GO:0042135 | biological_process | neurotransmitter catabolic process | 0.017833 |
| GO:0022607 | biological_process | cellular component assembly | 0.024392 |
| GO:0044085 | biological_process | cellular component biogenesis | 0.046048 |
| GO:0051289 | biological_process | protein homotetramerization | 0.074319 |
| GO:0051641 | biological_process | cellular localization | 0.013363 |
| GO:0051649 | biological_process | establishment of localization in cell | 0.054056 |
| GO:0006139 | biological_process | nucleobase, nucleoside, nucleotide and nucleic acid metabolic process | 0.026012 |
| GO:0006917 | biological_process | induction of apoptosis | 0.087413 |
| GO:0012502 | biological_process | induction of programmed cell death | 0.088392 |
| GO:0008633 | biological_process | activation of pro-apoptotic gene products | 0.055898 |
| GO:0002682 | biological_process | regulation of immune system process | 0.046066 |
| GO:0002684 | biological_process | positive regulation of immune system process | 0.035195 |
| GO:0002694 | biological_process | regulation of leukocyte activation | 0.055898 |
| GO:0002696 | biological_process | positive regulation of leukocyte activation | 0.019096 |
| GO:0045577 | biological_process | regulation of B cell differentiation | 0.00228 |
| GO:0045579 | biological_process | positive regulation of B cell differentiation | 0.000595 |
| GO:0045619 | biological_process | regulation of lymphocyte differentiation | 0.032279 |
| GO:0045621 | biological_process | positive regulation of lymphocyte differentiation | 0.01061 |
| GO:0050864 | biological_process | regulation of B cell activation | 0.023028 |
| GO:0050865 | biological_process | regulation of cell activation | 0.064224 |
| GO:0050867 | biological_process | positive regulation of cell activation | 0.021679 |
| GO:0050871 | biological_process | positive regulation of B cell activation | 0.01061 |
| GO:0051249 | biological_process | regulation of lymphocyte activation | 0.041541 |
| GO:0051251 | biological_process | positive regulation of lymphocyte activation | 0.015378 |
| GO:0045580 | biological_process | regulation of T cell differentiation | 0.023335 |
| GO:0045582 | biological_process | positive regulation of T cell differentiation | 0.00914 |
| GO:0050863 | biological_process | regulation of T cell activation | 0.024083 |
| GO:0050870 | biological_process | positive regulation of T cell activation | 0.008259 |
| GO:0000045 | biological_process | autophagic vacuole formation | 0.026012 |
| GO:0016050 | biological_process | vesicle organization | 0.035461 |
| GO:0016236 | biological_process | macroautophagy | 0.032279 |
| GO:0070925 | biological_process | organelle assembly | 0.011388 |
| GO:0009615 | biological_process | response to virus | 0.019096 |
| GO:0010506 | biological_process | regulation of autophagy | 0.010223 |
| GO:0032104 | biological_process | regulation of response to extracellular stimulus | 0.029127 |
| GO:0032106 | biological_process | positive regulation of response to extracellular stimulus | 0.00803 |
| GO:0032107 | biological_process | regulation of response to nutrient levels | 0.029127 |
| GO:0032109 | biological_process | positive regulation of response to nutrient levels | 0.00803 |
| GO:0001824 | biological_process | blastocyst development | 0.012776 |
| GO:0000768 | biological_process | syncytium formation by plasma membrane fusion | 0.026012 |
| GO:0006949 | biological_process | syncytium formation | 0.029127 |
| GO:0007520 | biological_process | myoblast fusion | 0.023335 |
| GO:0014902 | biological_process | myotube differentiation | 0.03833 |
| GO:0070646 | biological_process | protein modification by small protein removal | 0.087308 |
| GO:0009059 | biological_process | macromolecule biosynthetic process | 0.02808 |
| GO:0010467 | biological_process | gene expression | 0.029127 |
| GO:0016070 | biological_process | RNA metabolic process | 0.033495 |
| GO:0034645 | biological_process | cellular macromolecule biosynthetic process | 0.025161 |
| GO:0034961 | biological_process | cellular biopolymer biosynthetic process | 0.050581 |
| GO:0043284 | biological_process | biopolymer biosynthetic process | 0.05328 |
| GO:0006412 | biological_process | translation | 0.080916 |
| GO:0007595 | biological_process | lactation | 0.06014 |
| GO:0006357 | biological_process | regulation of transcription from RNA polymerase II promoter | 0.065134 |
| GO:0010551 | biological_process | regulation of gene-specific transcription from RNA polymerase II promoter | 0.082578 |
| GO:0010552 | biological_process | positive regulation of gene-specific transcription from RNA polymerase II promoter | 0.032279 |
| GO:0043193 | biological_process | positive regulation of gene-specific transcription | 0.072258 |
| GO:0021983 | biological_process | pituitary gland development | 0.071082 |
| GO:0040018 | biological_process | positive regulation of multicellular organism growth | 0.067768 |
| GO:0042752 | biological_process | regulation of circadian rhythm | 0.055898 |
| GO:0008064 | biological_process | regulation of actin polymerization or depolymerization | 0.031122 |
| GO:0030832 | biological_process | regulation of actin filament length | 0.033072 |
| GO:0032956 | biological_process | regulation of actin cytoskeleton organization | 0.06995 |
| GO:0032970 | biological_process | regulation of actin filament-based process | 0.07426 |
| GO:0033043 | biological_process | regulation of organelle organization | 0.026012 |
| GO:0051493 | biological_process | regulation of cytoskeleton organization | 0.029127 |
| GO:0002685 | biological_process | regulation of leukocyte migration | 0.055898 |
| GO:0002687 | biological_process | positive regulation of leukocyte migration | 0.03833 |
| GO:0002688 | biological_process | regulation of leukocyte chemotaxis | 0.026012 |
| GO:0002690 | biological_process | positive regulation of leukocyte chemotaxis | 0.023335 |
| GO:0040017 | biological_process | positive regulation of locomotion | 0.087308 |
| GO:0050920 | biological_process | regulation of chemotaxis | 0.095663 |
| GO:0050921 | biological_process | positive regulation of chemotaxis | 0.087308 |
| GO:0006206 | biological_process | pyrimidine base metabolic process | 0.017833 |
| GO:0009112 | biological_process | nucleobase metabolic process | 0.06014 |
| GO:0034404 | biological_process | nucleobase, nucleoside and nucleotide biosynthetic process | 0.048753 |
| GO:0034654 | biological_process | nucleobase, nucleoside, nucleotide and nucleic acid biosynthetic process | 0.048753 |
| GO:0046112 | biological_process | nucleobase biosynthetic process | 0.026012 |
| GO:0006221 | biological_process | pyrimidine nucleotide biosynthetic process | 0.067768 |
| GO:0006213 | biological_process | pyrimidine nucleoside metabolic process | 0.078415 |
| GO:0009156 | biological_process | ribonucleoside monophosphate biosynthetic process | 0.067768 |
| GO:0009161 | biological_process | ribonucleoside monophosphate metabolic process | 0.074319 |
| GO:0009218 | biological_process | pyrimidine ribonucleotide metabolic process | 0.045401 |
| GO:0009220 | biological_process | pyrimidine ribonucleotide biosynthetic process | 0.041541 |
| GO:0046131 | biological_process | pyrimidine ribonucleoside metabolic process | 0.048753 |
| GO:0031579 | biological_process | membrane raft organization | 0.012776 |
| GO:0051668 | biological_process | localization within membrane | 0.001974 |
| GO:0030010 | biological_process | establishment of cell polarity | 0.029127 |
| GO:0046649 | biological_process | lymphocyte activation | 0.07666 |
| GO:0009988 | biological_process | cell-cell recognition | 0.048753 |
| GO:0030098 | biological_process | lymphocyte differentiation | 0.084307 |
| GO:0030217 | biological_process | T cell differentiation | 0.03796 |
| GO:0033077 | biological_process | T cell differentiation in the thymus | 0.071082 |
| GO:0043368 | biological_process | positive T cell selection | 0.015378 |
| GO:0045058 | biological_process | T cell selection | 0.041541 |
| GO:0045059 | biological_process | positive thymic T cell selection | 0.010223 |
| GO:0045061 | biological_process | thymic T cell selection | 0.023335 |
| GO:0043383 | biological_process | negative T cell selection | 0.015378 |
| GO:0045060 | biological_process | negative thymic T cell selection | 0.012776 |
| GO:0042098 | biological_process | T cell proliferation | 0.06014 |
| GO:0046631 | biological_process | alpha-beta T cell activation | 0.003922 |
| GO:0048247 | biological_process | lymphocyte chemotaxis | 0.010223 |
| GO:0007015 | biological_process | actin filament organization | 0.043589 |
| GO:0008154 | biological_process | actin polymerization or depolymerization | 0.003922 |
| GO:0034621 | biological_process | cellular macromolecular complex subunit organization | 0.032422 |
| GO:0030834 | biological_process | regulation of actin filament depolymerization | 0.078415 |
| GO:0043243 | biological_process | positive regulation of protein complex disassembly | 0.015378 |
| GO:0043244 | biological_process | regulation of protein complex disassembly | 0.026012 |
| GO:0001755 | biological_process | neural crest cell migration | 0.048753 |
| GO:0014032 | biological_process | neural crest cell development | 0.087308 |
| GO:0014033 | biological_process | neural crest cell differentiation | 0.087308 |
| GO:0006413 | biological_process | translational initiation | 0.095663 |
| GO:0006084 | biological_process | acetyl-CoA metabolic process | 0.095663 |
| GO:0006099 | biological_process | tricarboxylic acid cycle | 0.064224 |
| GO:0009109 | biological_process | coenzyme catabolic process | 0.074319 |
| GO:0046356 | biological_process | acetyl-CoA catabolic process | 0.064224 |
| GO:0051187 | biological_process | cofactor catabolic process | 0.095663 |
| GO:0006108 | biological_process | malate metabolic process | 0.015378 |
| GO:0048873 | biological_process | homeostasis of number of cells within a tissue | 0.035461 |
| GO:0006418 | biological_process | tRNA aminoacylation for protein translation | 0.023335 |
| GO:0043038 | biological_process | amino acid activation | 0.023335 |
| GO:0043039 | biological_process | tRNA aminoacylation | 0.023335 |
| GO:0045055 | biological_process | regulated secretory pathway | 0.03833 |
| GO:0006107 | biological_process | oxaloacetate metabolic process | 0.02099 |
| GO:0009066 | biological_process | aspartate family amino acid metabolic process | 0.067768 |
| GO:0009067 | biological_process | aspartate family amino acid biosynthetic process | 0.035461 |
| GO:0009068 | biological_process | aspartate family amino acid catabolic process | 0.010223 |
| GO:0015718 | biological_process | monocarboxylic acid transport | 0.023335 |
| GO:0015908 | biological_process | fatty acid transport | 0.00803 |
| GO:0006538 | biological_process | glutamate catabolic process | 0.010223 |
| GO:0006103 | biological_process | 2-oxoglutarate metabolic process | 0.02099 |
| GO:0045471 | biological_process | response to ethanol | 0.041369 |
| GO:0006493 | biological_process | protein amino acid O-linked glycosylation | 0.071082 |
| GO:0043370 | biological_process | regulation of CD4-positive, alpha beta T cell differentiation | 0.032279 |
| GO:0043372 | biological_process | positive regulation of CD4-positive, alpha beta T cell differentiation | 0.02099 |
| GO:0045589 | biological_process | regulation of regulatory T cell differentiation | 0.00803 |
| GO:0046635 | biological_process | positive regulation of alpha-beta T cell activation | 0.067768 |
| GO:0046637 | biological_process | regulation of alpha-beta T cell differentiation | 0.064224 |
| GO:0046638 | biological_process | positive regulation of alpha-beta T cell differentiation | 0.045401 |
| GO:0033081 | biological_process | regulation of T cell differentiation in the thymus | 0.023335 |
| GO:0046632 | biological_process | alpha-beta T cell differentiation | 0.032279 |
| GO:0032655 | biological_process | regulation of interleukin-12 production | 0.03833 |
| GO:0045075 | biological_process | regulation of interleukin-12 biosynthetic process | 0.015378 |
| GO:0045084 | biological_process | positive regulation of interleukin-12 biosynthetic process | 0.012776 |
| GO:0006020 | biological_process | inositol metabolic process | 0.00228 |
| GO:0019751 | biological_process | polyol metabolic process | 0.016813 |
| GO:0043647 | biological_process | inositol phosphate metabolic process | 0.00803 |
| GO:0010970 | biological_process | microtubule-based transport | 0.074319 |
| GO:0034643 | biological_process | mitochondrion localization, microtubule-mediated | 0.00803 |
| GO:0047497 | biological_process | mitochondrion transport along microtubule | 0.00803 |
| GO:0051640 | biological_process | organelle localization | 0.072258 |
| GO:0051646 | biological_process | mitochondrion localization | 0.026012 |
| GO:0051654 | biological_process | establishment of mitochondrion localization | 0.00803 |
| GO:0051656 | biological_process | establishment of organelle localization | 0.041369 |
| GO:0051028 | biological_process | mRNA transport | 0.093431 |
| GO:0007026 | biological_process | negative regulation of microtubule depolymerization | 0.035461 |
| GO:0010639 | biological_process | negative regulation of organelle organization | 0.06995 |
| GO:0031110 | biological_process | regulation of microtubule polymerization or depolymerization | 0.064224 |
| GO:0031111 | biological_process | negative regulation of microtubule polymerization or depolymerization | 0.03833 |
| GO:0031114 | biological_process | regulation of microtubule depolymerization | 0.035461 |
| GO:0006417 | biological_process | regulation of translation | 0.038296 |
| GO:0010608 | biological_process | posttranscriptional regulation of gene expression | 0.027852 |
| GO:0031365 | biological_process | N-terminal protein amino acid modification | 0.032279 |
| GO:0007128 | biological_process | meiotic prophase I | 0.023335 |
| GO:0051324 | biological_process | prophase | 0.026012 |
| GO:0000288 | biological_process | nuclear-transcribed mRNA catabolic process, deadenylation-dependent decay | 0.00803 |
| GO:0000956 | biological_process | nuclear-transcribed mRNA catabolic process | 0.012776 |
| GO:0006396 | biological_process | RNA processing | 0.023496 |
| GO:0006397 | biological_process | mRNA processing | 0.029556 |
| GO:0006401 | biological_process | RNA catabolic process | 0.041369 |
| GO:0006402 | biological_process | mRNA catabolic process | 0.017833 |
| GO:0016071 | biological_process | mRNA metabolic process | 0.048753 |
| GO:0031123 | biological_process | RNA 3'-end processing | 0.074319 |
| GO:0031124 | biological_process | mRNA 3'-end processing | 0.03833 |
| GO:0007131 | biological_process | reciprocal meiotic recombination | 0.067768 |
| GO:0045132 | biological_process | meiotic chromosome segregation | 0.015378 |
| GO:0006298 | biological_process | mismatch repair | 0.078415 |
| GO:0000726 | biological_process | non-recombinational repair | 0.032279 |
| GO:0006303 | biological_process | double-strand break repair via nonhomologous end joining | 0.02099 |
| GO:0007140 | biological_process | male meiosis | 0.052106 |
| GO:0008630 | biological_process | DNA damage response, signal transduction resulting in induction of apoptosis | 0.09168 |
| GO:0002200 | biological_process | somatic diversification of immune receptors | 0.067768 |
| GO:0002377 | biological_process | immunoglobulin production | 0.071082 |
| GO:0002440 | biological_process | production of molecular mediator of immune response | 0.078415 |
| GO:0002566 | biological_process | somatic diversification of immune receptors via somatic mutation | 0.015378 |
| GO:0016445 | biological_process | somatic diversification of immunoglobulins | 0.048753 |
| GO:0016446 | biological_process | somatic hypermutation of immunoglobulin genes | 0.015378 |
| GO:0050000 | biological_process | chromosome localization | 0.035461 |
| GO:0051303 | biological_process | establishment of chromosome localization | 0.035461 |
| GO:0051310 | biological_process | metaphase plate congression | 0.023335 |
| GO:0002204 | biological_process | somatic recombination of immunoglobulin genes during immune response | 0.023335 |
| GO:0002208 | biological_process | somatic diversification of immunoglobulins during immune response | 0.023335 |
| GO:0002381 | biological_process | immunoglobulin production during immune response | 0.023335 |
| GO:0002562 | biological_process | somatic diversification of immune receptors via germline recombination within a single locus | 0.06014 |
| GO:0016444 | biological_process | somatic cell DNA recombination | 0.06014 |
| GO:0016447 | biological_process | somatic recombination of immunoglobulin gene segments | 0.03833 |
| GO:0045190 | biological_process | isotype switching | 0.023335 |
| GO:0000018 | biological_process | regulation of DNA recombination | 0.074319 |
| GO:0045910 | biological_process | negative regulation of DNA recombination | 0.012776 |
| GO:0034622 | biological_process | cellular macromolecular complex assembly | 0.074911 |
| GO:0051225 | biological_process | spindle assembly | 0.041541 |
| GO:0016254 | biological_process | preassembly of GPI anchor in ER membrane | 0.03833 |
| GO:0030384 | biological_process | phosphoinositide metabolic process | 0.054236 |
| GO:0001580 | biological_process | detection of chemical stimulus involved in sensory perception of bitter taste | 0.010223 |
| GO:0050906 | biological_process | detection of stimulus involved in sensory perception | 0.09168 |
| GO:0050907 | biological_process | detection of chemical stimulus involved in sensory perception | 0.017833 |
| GO:0050912 | biological_process | detection of chemical stimulus involved in sensory perception of taste | 0.015378 |
| GO:0050913 | biological_process | sensory perception of bitter taste | 0.017833 |
| GO:0006284 | biological_process | base-excision repair | 0.078415 |
| GO:0019439 | biological_process | aromatic compound catabolic process | 0.052106 |
| GO:0010043 | biological_process | response to zinc ion | 0.03833 |
| GO:0033198 | biological_process | response to ATP | 0.029127 |
| GO:0043542 | biological_process | endothelial cell migration | 0.064224 |
| GO:0046685 | biological_process | response to arsenic | 0.02099 |
| GO:0046839 | biological_process | phospholipid dephosphorylation | 0.00803 |
| GO:0048008 | biological_process | platelet-derived growth factor receptor signaling pathway | 0.045401 |
| GO:0030100 | biological_process | regulation of endocytosis | 0.03646 |
| GO:0045806 | biological_process | negative regulation of endocytosis | 0.002741 |
| GO:0050764 | biological_process | regulation of phagocytosis | 0.03833 |
| GO:0060627 | biological_process | regulation of vesicle-mediated transport | 0.084307 |
| GO:0051896 | biological_process | regulation of protein kinase B signaling cascade | 0.041541 |
| GO:0051898 | biological_process | negative regulation of protein kinase B signaling cascade | 0.00803 |
| GO:0006904 | biological_process | vesicle docking during exocytosis | 0.067768 |
| GO:0022406 | biological_process | membrane docking | 0.095663 |
| GO:0048278 | biological_process | vesicle docking | 0.074319 |
| GO:0042384 | biological_process | cilium assembly | 0.067768 |
| GO:0006906 | biological_process | vesicle fusion | 0.017833 |
| GO:0048284 | biological_process | organelle fusion | 0.041541 |
| GO:0010310 | biological_process | regulation of hydrogen peroxide metabolic process | 0.012776 |
| GO:0080010 | biological_process | regulation of oxygen and reactive oxygen species metabolic process | 0.023335 |
| GO:0060491 | biological_process | regulation of cell projection assembly | 0.045401 |
| GO:0030041 | biological_process | actin filament polymerization | 0.023335 |
| GO:0031529 | biological_process | ruffle organization | 0.015378 |
| GO:0046579 | biological_process | positive regulation of Ras protein signal transduction | 0.035461 |
| GO:0051057 | biological_process | positive regulation of small GTPase mediated signal transduction | 0.03833 |
| GO:0048259 | biological_process | regulation of receptor-mediated endocytosis | 0.03833 |
| GO:0048261 | biological_process | negative regulation of receptor-mediated endocytosis | 0.00803 |
| GO:0060263 | biological_process | regulation of respiratory burst | 0.012776 |
| GO:0000184 | biological_process | nuclear-transcribed mRNA catabolic process, nonsense-mediated decay | 0.09168 |
| GO:0000375 | biological_process | RNA splicing, via transesterification reactions | 0.010921 |
| GO:0000377 | biological_process | RNA splicing, via transesterification reactions with bulged adenosine as nucleophile | 0.010921 |
| GO:0000398 | biological_process | nuclear mRNA splicing, via spliceosome | 0.010921 |
| GO:0008380 | biological_process | RNA splicing | 0.067752 |
| GO:0006337 | biological_process | nucleosome disassembly | 0.010223 |
| GO:0031498 | biological_process | chromatin disassembly | 0.010223 |
| GO:0032986 | biological_process | protein-DNA complex disassembly | 0.010223 |
| GO:0034623 | biological_process | cellular macromolecular complex disassembly | 0.067768 |
| GO:0031056 | biological_process | regulation of histone modification | 0.029127 |
| GO:0031057 | biological_process | negative regulation of histone modification | 0.012776 |
| GO:0035065 | biological_process | regulation of histone acetylation | 0.00803 |
| GO:0007090 | biological_process | regulation of S phase of mitotic cell cycle | 0.03833 |
| GO:0010948 | biological_process | negative regulation of cell cycle process | 0.071082 |
| GO:0033261 | biological_process | regulation of S phase | 0.052106 |
| GO:0045749 | biological_process | negative regulation of S phase of mitotic cell cycle | 0.017833 |
| GO:0045930 | biological_process | negative regulation of mitotic cell cycle | 0.055898 |
| GO:0006613 | biological_process | cotranslational protein targeting to membrane | 0.026012 |
| GO:0006614 | biological_process | SRP-dependent cotranslational protein targeting to membrane | 0.010223 |
| GO:0045047 | biological_process | protein targeting to ER | 0.017833 |
| GO:0070972 | biological_process | protein localization in endoplasmic reticulum | 0.017833 |
| GO:0006448 | biological_process | regulation of translational elongation | 0.012776 |
| GO:0016246 | biological_process | RNA interference | 0.010223 |
| GO:0016441 | biological_process | posttranscriptional gene silencing | 0.055898 |
| GO:0031050 | biological_process | dsRNA fragmentation | 0.029127 |
| GO:0035194 | biological_process | posttranscriptional gene silencing by RNA | 0.055898 |
| GO:0043331 | biological_process | response to dsRNA | 0.064224 |
| GO:0070918 | biological_process | production of small RNA involved in gene silencing by RNA | 0.029127 |
| GO:0031054 | biological_process | pre-microRNA processing | 0.010223 |
| GO:0035195 | biological_process | gene silencing by miRNA | 0.041541 |
| GO:0035196 | biological_process | production of miRNAs involved in gene silencing by miRNA | 0.026012 |
| GO:0045069 | biological_process | regulation of viral genome replication | 0.032279 |
| GO:0048524 | biological_process | positive regulation of viral reproduction | 0.026012 |
| GO:0050792 | biological_process | regulation of viral reproduction | 0.064224 |
| GO:0046782 | biological_process | regulation of viral transcription | 0.017833 |
| GO:0002698 | biological_process | negative regulation of immune effector process | 0.035461 |
| GO:0002831 | biological_process | regulation of response to biotic stimulus | 0.087308 |
| GO:0043900 | biological_process | regulation of multi-organism process | 0.095663 |
| GO:0050688 | biological_process | regulation of defense response to virus | 0.052106 |
| GO:0050691 | biological_process | regulation of defense response to virus by host | 0.02099 |
| GO:0001825 | biological_process | blastocyst formation | 0.041541 |
| GO:0001829 | biological_process | trophectodermal cell differentiation | 0.032279 |
| GO:0007566 | biological_process | embryo implantation | 0.074319 |
| GO:0005622 | cellular_component | intracellular | 0.025215 |
| GO:0005623 | cellular_component | cell | 0.017833 |
| GO:0005737 | cellular_component | cytoplasm | 0.052106 |
| GO:0044424 | cellular_component | intracellular part | 0.023215 |
| GO:0044464 | cellular_component | cell part | 0.017833 |
| GO:0005739 | cellular_component | mitochondrion | 0.00601 |
| GO:0043226 | cellular_component | organelle | 0.017833 |
| GO:0043227 | cellular_component | membrane-bounded organelle | 0.023335 |
| GO:0043229 | cellular_component | intracellular organelle | 0.017311 |
| GO:0043231 | cellular_component | intracellular membrane-bounded organelle | 0.023335 |
| GO:0044444 | cellular_component | cytoplasmic part | 0.010223 |
| GO:0005759 | cellular_component | mitochondrial matrix | 0.009727 |
| GO:0031974 | cellular_component | membrane-enclosed lumen | 0.049806 |
| GO:0031980 | cellular_component | mitochondrial lumen | 0.009727 |
| GO:0043233 | cellular_component | organelle lumen | 0.043852 |
| GO:0044422 | cellular_component | organelle part | 0.013363 |
| GO:0044429 | cellular_component | mitochondrial part | 0.011162 |
| GO:0044446 | cellular_component | intracellular organelle part | 0.012776 |
| GO:0070013 | cellular_component | intracellular organelle lumen | 0.038049 |
| GO:0005625 | cellular_component | soluble fraction | 0.075871 |
| GO:0048471 | cellular_component | perinuclear region of cytoplasm | 0.071137 |
| GO:0005829 | cellular_component | cytosol | 0.036785 |
| GO:0032991 | cellular_component | macromolecular complex | 0.047516 |
| GO:0033176 | cellular_component | proton-transporting V-type ATPase complex | 0.052106 |
| GO:0033178 | cellular_component | proton-transporting two-sector ATPase complex, catalytic domain | 0.048753 |
| GO:0033180 | cellular_component | proton-transporting V-type ATPase, V1 domain | 0.017833 |
| GO:0043234 | cellular_component | protein complex | 0.064224 |
| GO:0005741 | cellular_component | mitochondrial outer membrane | 0.074838 |
| GO:0019867 | cellular_component | outer membrane | 0.021096 |
| GO:0031967 | cellular_component | organelle envelope | 0.035195 |
| GO:0031968 | cellular_component | organelle outer membrane | 0.019096 |
| GO:0031975 | cellular_component | envelope | 0.035461 |
| GO:0044428 | cellular_component | nuclear part | 0.010223 |
| GO:0005852 | cellular_component | eukaryotic translation initiation factor 3 complex | 0.032279 |
| GO:0008180 | cellular_component | signalosome | 0.02099 |
| GO:0019866 | cellular_component | organelle inner membrane | 0.095663 |
| GO:0030863 | cellular_component | cortical cytoskeleton | 0.018925 |
| GO:0030864 | cellular_component | cortical actin cytoskeleton | 0.074319 |
| GO:0044448 | cellular_component | cell cortex part | 0.057183 |
| GO:0045239 | cellular_component | tricarboxylic acid cycle enzyme complex | 0.010223 |
| GO:0005637 | cellular_component | nuclear inner membrane | 0.006083 |
| GO:0005639 | cellular_component | integral to nuclear inner membrane | 0.00803 |
| GO:0031229 | cellular_component | intrinsic to nuclear inner membrane | 0.00803 |
| GO:0031965 | cellular_component | nuclear membrane | 0.060301 |
| GO:0044453 | cellular_component | nuclear membrane part | 0.00803 |
| GO:0005643 | cellular_component | nuclear pore | 0.050131 |
| GO:0046930 | cellular_component | pore complex | 0.071082 |
| GO:0000794 | cellular_component | condensed nuclear chromosome | 0.021679 |
| GO:0005640 | cellular_component | nuclear outer membrane | 0.032279 |
| GO:0000795 | cellular_component | synaptonemal complex | 0.045401 |
| GO:0001673 | cellular_component | male germ cell nucleus | 0.03833 |
| GO:0043073 | cellular_component | germ cell nucleus | 0.048753 |
| GO:0032300 | cellular_component | mismatch repair complex | 0.012776 |
| GO:0043209 | cellular_component | myelin sheath | 0.029127 |
| GO:0005876 | cellular_component | spindle microtubule | 0.087308 |
| GO:0031513 | cellular_component | nonmotile primary cilium | 0.095663 |
| GO:0030532 | cellular_component | small nuclear ribonucleoprotein complex | 0.006083 |
| GO:0016514 | cellular_component | SWI/SNF complex | 0.035461 |
| GO:0070603 | cellular_component | SWI/SNF-type complex | 0.035461 |
| GO:0005791 | cellular_component | rough endoplasmic reticulum | 0.052106 |
| GO:0030867 | cellular_component | rough endoplasmic reticulum membrane | 0.017833 |
| GO:0005786 | cellular_component | signal recognition particle, endoplasmic reticulum targeting | 0.010223 |
| GO:0048500 | cellular_component | signal recognition particle | 0.010223 |
| GO:0005742 | cellular_component | mitochondrial outer membrane translocase complex | 0.012776 |
| GO:0005488 | molecular_function | binding | 0.019955 |
| GO:0004675 | molecular_function | transmembrane receptor protein serine/threonine kinase activity | 0.041541 |
| GO:0005024 | molecular_function | transforming growth factor beta receptor activity | 0.041541 |
| GO:0005025 | molecular_function | transforming growth factor beta receptor activity, type I | 0.010223 |
| GO:0017002 | molecular_function | activin receptor activity | 0.010223 |
| GO:0005515 | molecular_function | protein binding | 0.001489 |
| GO:0032403 | molecular_function | protein complex binding | 0.092929 |
| GO:0048185 | molecular_function | activin binding | 0.02099 |
| GO:0050431 | molecular_function | transforming growth factor beta binding | 0.029127 |
| GO:0016645 | molecular_function | oxidoreductase activity, acting on the CH-NH group of donors | 0.078415 |
| GO:0016646 | molecular_function | oxidoreductase activity, acting on the CH-NH group of donors, NAD or NADP as acceptor | 0.041541 |
| GO:0004029 | molecular_function | aldehyde dehydrogenase (NAD) activity | 0.015378 |
| GO:0016620 | molecular_function | oxidoreductase activity, acting on the aldehyde or oxo group of donors, NAD or NADP as acceptor | 0.005745 |
| GO:0016903 | molecular_function | oxidoreductase activity, acting on the aldehyde or oxo group of donors | 0.01061 |
| GO:0046933 | molecular_function | hydrogen ion transporting ATP synthase activity, rotational mechanism | 0.032279 |
| GO:0046961 | molecular_function | proton-transporting ATPase activity, rotational mechanism | 0.048753 |
| GO:0004198 | molecular_function | calcium-dependent cysteine-type endopeptidase activity | 0.03833 |
| GO:0003676 | molecular_function | nucleic acid binding | 0.043589 |
| GO:0003743 | molecular_function | translation initiation factor activity | 0.027958 |
| GO:0008135 | molecular_function | translation factor activity, nucleic acid binding | 0.07426 |
| GO:0003690 | molecular_function | double-stranded DNA binding | 0.095663 |
| GO:0043566 | molecular_function | structure-specific DNA binding | 0.044302 |
| GO:0016627 | molecular_function | oxidoreductase activity, acting on the CH-CH group of donors | 0.026651 |
| GO:0016634 | molecular_function | oxidoreductase activity, acting on the CH-CH group of donors, oxygen as acceptor | 0.017833 |
| GO:0005099 | molecular_function | Ras GTPase activator activity | 0.084307 |
| GO:0005100 | molecular_function | Rho GTPase activator activity | 0.08335 |
| GO:0030675 | molecular_function | Rac GTPase activator activity | 0.015378 |
| GO:0030676 | molecular_function | Rac guanyl-nucleotide exchange factor activity | 0.010223 |
| GO:0046875 | molecular_function | ephrin receptor binding | 0.015378 |
| GO:0003723 | molecular_function | RNA binding | 0.033502 |
| GO:0005527 | molecular_function | macrolide binding | 0.015378 |
| GO:0005528 | molecular_function | FK506 binding | 0.015378 |
| GO:0004812 | molecular_function | aminoacyl-tRNA ligase activity | 0.023805 |
| GO:0016875 | molecular_function | ligase activity, forming carbon-oxygen bonds | 0.023805 |
| GO:0016876 | molecular_function | ligase activity, forming aminoacyl-tRNA and related compounds | 0.023805 |
| GO:0008483 | molecular_function | transaminase activity | 0.055898 |
| GO:0016769 | molecular_function | transferase activity, transferring nitrogenous groups | 0.09168 |
| GO:0005095 | molecular_function | GTPase inhibitor activity | 0.02099 |
| GO:0051766 | molecular_function | inositol trisphosphate kinase activity | 0.015378 |
| GO:0005521 | molecular_function | lamin binding | 0.010223 |
| GO:0016628 | molecular_function | oxidoreductase activity, acting on the CH-CH group of donors, NAD or NADP as acceptor | 0.048753 |
| GO:0003697 | molecular_function | single-stranded DNA binding | 0.029675 |
| GO:0008017 | molecular_function | microtubule binding | 0.046066 |
| GO:0015631 | molecular_function | tubulin binding | 0.089578 |
| GO:0048487 | molecular_function | beta-tubulin binding | 0.03833 |
| GO:0004177 | molecular_function | aminopeptidase activity | 0.095663 |
| GO:0050897 | molecular_function | cobalt ion binding | 0.041541 |
| GO:0030983 | molecular_function | mismatched DNA binding | 0.055898 |
| GO:0032404 | molecular_function | mismatch repair complex binding | 0.015378 |
| GO:0032407 | molecular_function | MutSalpha complex binding | 0.00803 |
| GO:0043531 | molecular_function | ADP binding | 0.048753 |
| GO:0001608 | molecular_function | nucleotide receptor activity, G-protein coupled | 0.09168 |
| GO:0045028 | molecular_function | purinergic nucleotide receptor activity, G-protein coupled | 0.09168 |
| GO:0017176 | molecular_function | phosphatidylinositol N-acetylglucosaminyltransferase activity | 0.00803 |
| GO:0004434 | molecular_function | inositol or phosphatidylinositol phosphodiesterase activity | 0.071082 |
| GO:0004435 | molecular_function | phosphoinositide phospholipase C activity | 0.071082 |
| GO:0004629 | molecular_function | phospholipase C activity | 0.095663 |
| GO:0004437 | molecular_function | inositol or phosphatidylinositol phosphatase activity | 0.071082 |
| GO:0042577 | molecular_function | lipid phosphatase activity | 0.015378 |
| GO:0005161 | molecular_function | platelet-derived growth factor receptor binding | 0.023335 |
| GO:0034593 | molecular_function | phosphatidylinositol bisphosphate phosphatase activity | 0.017833 |
| GO:0030742 | molecular_function | GTP-dependent protein binding | 0.023335 |
| GO:0004864 | molecular_function | phosphoprotein phosphatase inhibitor activity | 0.074319 |
| GO:0019212 | molecular_function | phosphatase inhibitor activity | 0.08335 |
| GO:0008601 | molecular_function | protein phosphatase type 2A regulator activity | 0.041541 |
| GO:0042393 | molecular_function | histone binding | 0.019955 |
| GO:0008028 | molecular_function | monocarboxylic acid transmembrane transporter activity | 0.067768 |
| GO:0002039 | molecular_function | p53 binding | 0.041541 |
| GO:0008312 | molecular_function | 7S RNA binding | 0.010223 |
| GO:0005062 | molecular_function | hematopoietin/interferon-class (D200-domain) cytokine receptor signal transducer activity | 0.010223 |
| GO:0035197 | molecular_function | siRNA binding | 0.00803 |
| GO:0008320 | molecular_function | protein transmembrane transporter activity | 0.041541 |
| GO:0015450 | molecular_function | P-P-bond-hydrolysis-driven protein transmembrane transporter activity | 0.026012 |
| GO:0022884 | molecular_function | macromolecule transmembrane transporter activity | 0.026012 |
