# Supplementary material for: Genes and Gene Ontologies Common to Airflow Obstruction and Emphysema in the Lungs of Patients with COPD
Source: PLoS One. 2011 Mar 15;6(3):e17442. doi: 10.1371/journal.pone.0017442 (PMC3057973; doi:10.1371/journal.pone.0017442)
Supplement: Table S9 — Gene ontologies enriched in Savarimuthu et al TPCH-KCO dataset. (DOCX) [file pone.0017442.s011.docx]

**Table S9: Gene ontologies enriched in Savarimuthu *et al* dataset using KCO to classify mild and moderate emphysema subjects**

| **GOID** | **Ontology** | **Term** | **p** |
| --- | --- | --- | --- |
| GO:0010149 | biological_process | senescence | 9.49E-07 |
| GO:0004861 | molecular_function | cyclin-dependent protein kinase inhibitor activity | 2.48E-06 |
| GO:0045736 | biological_process | negative regulation of cyclin-dependent protein kinase activity | 2.48E-06 |
| GO:0016538 | molecular_function | cyclin-dependent protein kinase regulator activity | 9.68E-06 |
| GO:0030291 | molecular_function | protein serine/threonine kinase inhibitor activity | 1.10E-05 |
| GO:0004857 | molecular_function | enzyme inhibitor activity | 1.30E-05 |
| GO:0004860 | molecular_function | protein kinase inhibitor activity | 6.36E-05 |
| GO:0019210 | molecular_function | kinase inhibitor activity | 6.49E-05 |
| GO:0000079 | biological_process | regulation of cyclin-dependent protein kinase activity | 0.000164 |
| GO:0001652 | cellular_component | granular component | 0.000164 |
| GO:0010389 | biological_process | regulation of G2/M transition of mitotic cell cycle | 0.000164 |
| GO:0043618 | biological_process | regulation of transcription from RNA polymerase II promoter in response to stress | 0.000164 |
| GO:0043620 | biological_process | regulation of transcription in response to stress | 0.000164 |
| GO:0046822 | biological_process | regulation of nucleocytoplasmic transport | 0.000165 |
| GO:0070761 | cellular_component | pre-snoRNP complex | 0.000225 |
| GO:0006622 | biological_process | protein targeting to lysosome | 0.000225 |
| GO:0042921 | biological_process | glucocorticoid receptor signaling pathway | 0.000225 |
| GO:0032386 | biological_process | regulation of intracellular transport | 0.000285 |
| GO:0048103 | biological_process | somatic stem cell division | 0.000285 |
| GO:0017080 | molecular_function | sodium channel regulator activity | 0.000285 |
| GO:0031958 | biological_process | corticosteroid receptor signaling pathway | 0.000285 |
| GO:0001885 | biological_process | endothelial cell development | 0.000285 |
| GO:0030889 | biological_process | negative regulation of B cell proliferation | 0.00036 |
| GO:0070667 | biological_process | negative regulation of mast cell proliferation | 0.00036 |
| GO:0070064 | molecular_function | proline-rich region binding | 0.00036 |
| GO:0045786 | biological_process | negative regulation of cell cycle | 0.000384 |
| GO:0019887 | molecular_function | protein kinase regulator activity | 0.000407 |
| GO:0030934 | cellular_component | anchoring collagen | 0.000411 |
| GO:0001953 | biological_process | negative regulation of cell-matrix adhesion | 0.000411 |
| GO:0030308 | biological_process | negative regulation of cell growth | 0.000411 |
| GO:0017145 | biological_process | stem cell division | 0.000411 |
| GO:0006623 | biological_process | protein targeting to vacuole | 0.000411 |
| GO:0006469 | biological_process | negative regulation of protein kinase activity | 0.000456 |
| GO:0010812 | biological_process | negative regulation of cell-substrate adhesion | 0.000491 |
| GO:0045792 | biological_process | negative regulation of cell size | 0.000491 |
| GO:0033673 | biological_process | negative regulation of kinase activity | 0.000498 |
| GO:0019207 | molecular_function | kinase regulator activity | 0.000524 |
| GO:0004867 | molecular_function | serine-type endopeptidase inhibitor activity | 0.000533 |
| GO:0033081 | biological_process | regulation of T cell differentiation in the thymus | 0.000557 |
| GO:0031623 | biological_process | receptor internalization | 0.000557 |
| GO:0051348 | biological_process | negative regulation of transferase activity | 0.000561 |
| GO:0045926 | biological_process | negative regulation of growth | 0.000633 |
| GO:0014068 | biological_process | positive regulation of phosphoinositide 3-kinase cascade | 0.000633 |
| GO:0045309 | molecular_function | protein phosphorylated amino acid binding | 0.000633 |
| GO:0007568 | biological_process | aging | 0.000672 |
| GO:0007050 | biological_process | cell cycle arrest | 0.000682 |
| GO:0002028 | biological_process | regulation of sodium ion transport | 0.00071 |
| GO:0014066 | biological_process | regulation of phosphoinositide 3-kinase cascade | 0.00071 |
| GO:0008200 | molecular_function | ion channel inhibitor activity | 0.000818 |
| GO:0030234 | molecular_function | enzyme regulator activity | 0.000877 |
| GO:0032088 | biological_process | negative regulation of NF-kappaB transcription factor activity | 0.000877 |
| GO:0050869 | biological_process | negative regulation of B cell activation | 0.000877 |
| GO:0016248 | molecular_function | channel inhibitor activity | 0.000877 |
| GO:0003158 | biological_process | endothelium development | 0.000877 |
| GO:0045446 | biological_process | endothelial cell differentiation | 0.000877 |
| GO:0051059 | molecular_function | NF-kappaB binding | 0.00098 |
| GO:0042787 | biological_process | protein ubiquitination during ubiquitin-dependent protein catabolic process | 0.00098 |
| GO:0006309 | biological_process | DNA fragmentation involved in apoptosis | 0.001085 |
| GO:0009303 | biological_process | rRNA transcription | 0.001085 |
| GO:0043271 | biological_process | negative regulation of ion transport | 0.001349 |
| GO:0004866 | molecular_function | endopeptidase inhibitor activity | 0.001539 |
| GO:0006921 | biological_process | cell structure disassembly during apoptosis | 0.001586 |
| GO:0046824 | biological_process | positive regulation of nucleocytoplasmic transport | 0.001586 |
| GO:0004364 | molecular_function | glutathione transferase activity | 0.001586 |
| GO:0030414 | molecular_function | peptidase inhibitor activity | 0.001724 |
| GO:0016525 | biological_process | negative regulation of angiogenesis | 0.00187 |
| GO:0000737 | biological_process | DNA catabolic process, endonucleolytic | 0.001979 |
| GO:0030262 | biological_process | apoptotic nuclear changes | 0.001979 |
| GO:0007041 | biological_process | lysosomal transport | 0.001979 |
| GO:0001952 | biological_process | regulation of cell-matrix adhesion | 0.002283 |
| GO:0001558 | biological_process | regulation of cell growth | 0.002283 |
| GO:0008361 | biological_process | regulation of cell size | 0.002298 |
| GO:0044452 | cellular_component | nucleolar part | 0.002298 |
| GO:0022613 | biological_process | ribonucleoprotein complex biogenesis | 0.002298 |
| GO:0030888 | biological_process | regulation of B cell proliferation | 0.002298 |
| GO:0042130 | biological_process | negative regulation of T cell proliferation | 0.002298 |
| GO:0032182 | molecular_function | small conjugating protein binding | 0.002298 |
| GO:0043130 | molecular_function | ubiquitin binding | 0.002298 |
| GO:0051219 | molecular_function | phosphoprotein binding | 0.002298 |
| GO:0070666 | biological_process | regulation of mast cell proliferation | 0.002417 |
| GO:0050728 | biological_process | negative regulation of inflammatory response | 0.002417 |
| GO:0032502 | biological_process | developmental process | 0.00245 |
| GO:0008637 | biological_process | apoptotic mitochondrial changes | 0.002506 |
| GO:0043112 | biological_process | receptor metabolic process | 0.002506 |
| GO:0032388 | biological_process | positive regulation of intracellular transport | 0.002506 |
| GO:0007034 | biological_process | vacuolar transport | 0.002844 |
| GO:0032945 | biological_process | negative regulation of mononuclear cell proliferation | 0.002957 |
| GO:0050672 | biological_process | negative regulation of lymphocyte proliferation | 0.002957 |
| GO:0070664 | biological_process | negative regulation of leukocyte proliferation | 0.002957 |
| GO:0007569 | biological_process | cell aging | 0.003096 |
| GO:0031348 | biological_process | negative regulation of defense response | 0.003096 |
| GO:0005581 | cellular_component | collagen | 0.003431 |
| GO:0045732 | biological_process | positive regulation of protein catabolic process | 0.003431 |
| GO:0007422 | biological_process | peripheral nervous system development | 0.003602 |
| GO:0051701 | biological_process | interaction with host | 0.003777 |
| GO:0007162 | biological_process | negative regulation of cell adhesion | 0.004135 |
| GO:0042326 | biological_process | negative regulation of phosphorylation | 0.004135 |
| GO:0031647 | biological_process | regulation of protein stability | 0.004317 |
| GO:0060341 | biological_process | regulation of cellular localization | 0.004377 |
| GO:0032535 | biological_process | regulation of cellular component size | 0.004397 |
| GO:0044403 | biological_process | symbiosis, encompassing mutualism through parasitism | 0.004435 |
| GO:0010810 | biological_process | regulation of cell-substrate adhesion | 0.004453 |
| GO:0043433 | biological_process | negative regulation of transcription factor activity | 0.004453 |
| GO:0090048 | biological_process | negative regulation of transcription regulator activity | 0.004453 |
| GO:0010563 | biological_process | negative regulation of phosphorus metabolic process | 0.004453 |
| GO:0045936 | biological_process | negative regulation of phosphate metabolic process | 0.004453 |
| GO:0050868 | biological_process | negative regulation of T cell activation | 0.004453 |
| GO:0048731 | biological_process | system development | 0.004563 |
| GO:0032102 | biological_process | negative regulation of response to external stimulus | 0.005043 |
| GO:0050864 | biological_process | regulation of B cell activation | 0.005465 |
| GO:0000082 | biological_process | G1/S transition of mitotic cell cycle | 0.005582 |
| GO:0043392 | biological_process | negative regulation of DNA binding | 0.005582 |
| GO:0045580 | biological_process | regulation of T cell differentiation | 0.005582 |
| GO:0006898 | biological_process | receptor-mediated endocytosis | 0.005976 |
| GO:0016765 | molecular_function | transferase activity, transferring alkyl or aryl (other than methyl) groups | 0.005976 |
| GO:0090066 | biological_process | regulation of anatomical structure size | 0.005991 |
| GO:0048856 | biological_process | anatomical structure development | 0.006053 |
| GO:0006997 | biological_process | nucleus organization | 0.006053 |
| GO:0051250 | biological_process | negative regulation of lymphocyte activation | 0.006053 |
| GO:0043086 | biological_process | negative regulation of catalytic activity | 0.006085 |
| GO:0006919 | biological_process | activation of caspase activity | 0.00621 |
| GO:0002695 | biological_process | negative regulation of leukocyte activation | 0.006655 |
| GO:0009411 | biological_process | response to UV | 0.006815 |
| GO:0016247 | molecular_function | channel regulator activity | 0.006815 |
| GO:0051100 | biological_process | negative regulation of binding | 0.006932 |
| GO:0030518 | biological_process | steroid hormone receptor signaling pathway | 0.006932 |
| GO:0050769 | biological_process | positive regulation of neurogenesis | 0.006932 |
| GO:0006308 | biological_process | DNA catabolic process | 0.006962 |
| GO:0010952 | biological_process | positive regulation of peptidase activity | 0.006962 |
| GO:0043280 | biological_process | positive regulation of caspase activity | 0.006962 |
| GO:0045619 | biological_process | regulation of lymphocyte differentiation | 0.006962 |
| GO:0009896 | biological_process | positive regulation of catabolic process | 0.006962 |
| GO:0050866 | biological_process | negative regulation of cell activation | 0.007121 |
| GO:0051592 | biological_process | response to calcium ion | 0.007121 |
| GO:0006917 | biological_process | induction of apoptosis | 0.007516 |
| GO:0012502 | biological_process | induction of programmed cell death | 0.007516 |
| GO:0016597 | molecular_function | amino acid binding | 0.007516 |
| GO:0042383 | cellular_component | sarcolemma | 0.007691 |
| GO:0051726 | biological_process | regulation of cell cycle | 0.007717 |
| GO:0040008 | biological_process | regulation of growth | 0.007717 |
| GO:0042129 | biological_process | regulation of T cell proliferation | 0.007717 |
| GO:0022415 | biological_process | viral reproductive process | 0.007717 |
| GO:0045765 | biological_process | regulation of angiogenesis | 0.007717 |
| GO:0048468 | biological_process | cell development | 0.007729 |
| GO:0051352 | biological_process | negative regulation of ligase activity | 0.008084 |
| GO:0051444 | biological_process | negative regulation of ubiquitin-protein ligase activity | 0.008084 |
| GO:0022411 | biological_process | cellular component disassembly | 0.008292 |
| GO:0010720 | biological_process | positive regulation of cell development | 0.008501 |
| GO:0044092 | biological_process | negative regulation of molecular function | 0.008565 |
| GO:0045859 | biological_process | regulation of protein kinase activity | 0.008744 |
| GO:0008285 | biological_process | negative regulation of cell proliferation | 0.009001 |
| GO:0022618 | biological_process | ribonucleoprotein complex assembly | 0.009087 |
| GO:0043549 | biological_process | regulation of kinase activity | 0.009369 |
| GO:0030522 | biological_process | intracellular receptor-mediated signaling pathway | 0.009517 |
| GO:0050727 | biological_process | regulation of inflammatory response | 0.009734 |
| GO:0010959 | biological_process | regulation of metal ion transport | 0.010226 |
| GO:0051338 | biological_process | regulation of transferase activity | 0.010293 |
| GO:0051438 | biological_process | regulation of ubiquitin-protein ligase activity | 0.010337 |
| GO:0042176 | biological_process | regulation of protein catabolic process | 0.010337 |
| GO:0043281 | biological_process | regulation of caspase activity | 0.010837 |
| GO:0048519 | biological_process | negative regulation of biological process | 0.010838 |
| GO:0007275 | biological_process | multicellular organismal development | 0.010892 |
| GO:0002683 | biological_process | negative regulation of immune system process | 0.010892 |
| GO:0051340 | biological_process | regulation of ligase activity | 0.010892 |
| GO:0048522 | biological_process | positive regulation of cellular process | 0.011308 |
| GO:0052548 | biological_process | regulation of endopeptidase activity | 0.011308 |
| GO:0016032 | biological_process | viral reproduction | 0.011308 |
| GO:0050670 | biological_process | regulation of lymphocyte proliferation | 0.011791 |
| GO:0032944 | biological_process | regulation of mononuclear cell proliferation | 0.011959 |
| GO:0070663 | biological_process | regulation of leukocyte proliferation | 0.011959 |
| GO:0052547 | biological_process | regulation of peptidase activity | 0.012187 |
| GO:0000075 | biological_process | cell cycle checkpoint | 0.012708 |
| GO:0042470 | cellular_component | melanosome | 0.012877 |
| GO:0048770 | cellular_component | pigment granule | 0.012877 |
| GO:0000151 | cellular_component | ubiquitin ligase complex | 0.013109 |
| GO:0043269 | biological_process | regulation of ion transport | 0.013343 |
| GO:0043065 | biological_process | positive regulation of apoptosis | 0.013964 |
| GO:0006364 | biological_process | rRNA processing | 0.014048 |
| GO:0051049 | biological_process | regulation of transport | 0.014048 |
| GO:0043068 | biological_process | positive regulation of programmed cell death | 0.014057 |
| GO:0010942 | biological_process | positive regulation of cell death | 0.014186 |
| GO:0051329 | biological_process | interphase of mitotic cell cycle | 0.014388 |
| GO:0048585 | biological_process | negative regulation of response to stimulus | 0.014388 |
| GO:0016072 | biological_process | rRNA metabolic process | 0.014933 |
| GO:0051325 | biological_process | interphase | 0.015033 |
| GO:0007399 | biological_process | nervous system development | 0.015033 |
| GO:0043627 | biological_process | response to estrogen stimulus | 0.015033 |
| GO:0048518 | biological_process | positive regulation of biological process | 0.01594 |
| GO:0051090 | biological_process | regulation of transcription factor activity | 0.016068 |
| GO:0090046 | biological_process | regulation of transcription regulator activity | 0.016068 |
| GO:0042325 | biological_process | regulation of phosphorylation | 0.016125 |
| GO:0016327 | cellular_component | apicolateral plasma membrane | 0.016238 |
| GO:0010564 | biological_process | regulation of cell cycle process | 0.01773 |
| GO:0045595 | biological_process | regulation of cell differentiation | 0.01773 |
| GO:0019220 | biological_process | regulation of phosphate metabolic process | 0.017794 |
| GO:0051174 | biological_process | regulation of phosphorus metabolic process | 0.017794 |
| GO:0044420 | cellular_component | extracellular matrix part | 0.018037 |
| GO:0001664 | molecular_function | G-protein-coupled receptor binding | 0.018037 |
| GO:0016567 | biological_process | protein ubiquitination | 0.018618 |
| GO:0043176 | molecular_function | amine binding | 0.018869 |
| GO:0050863 | biological_process | regulation of T cell activation | 0.019458 |
| GO:0051101 | biological_process | regulation of DNA binding | 0.021003 |
| GO:0009894 | biological_process | regulation of catabolic process | 0.021003 |
| GO:0019899 | molecular_function | enzyme binding | 0.022138 |
| GO:0042254 | biological_process | ribosome biogenesis | 0.022138 |
| GO:0032446 | biological_process | protein modification by small protein conjugation | 0.022138 |
| GO:0048699 | biological_process | generation of neurons | 0.022536 |
| GO:0030155 | biological_process | regulation of cell adhesion | 0.023289 |
| GO:0030855 | biological_process | epithelial cell differentiation | 0.023289 |
| GO:0051051 | biological_process | negative regulation of transport | 0.024556 |
| GO:0009416 | biological_process | response to light stimulus | 0.024556 |
| GO:0007005 | biological_process | mitochondrion organization | 0.025094 |
| GO:0010038 | biological_process | response to metal ion | 0.025094 |
| GO:0005938 | cellular_component | cell cortex | 0.025364 |
| GO:0031347 | biological_process | regulation of defense response | 0.026389 |
| GO:0022008 | biological_process | neurogenesis | 0.027227 |
| GO:0022402 | biological_process | cell cycle process | 0.027539 |
| GO:0051249 | biological_process | regulation of lymphocyte activation | 0.027981 |
| GO:0031406 | molecular_function | carboxylic acid binding | 0.028257 |
| GO:0019901 | molecular_function | protein kinase binding | 0.028421 |
| GO:0004842 | molecular_function | ubiquitin-protein ligase activity | 0.028421 |
| GO:0032101 | biological_process | regulation of response to external stimulus | 0.029088 |
| GO:0032879 | biological_process | regulation of localization | 0.030487 |
| GO:0051098 | biological_process | regulation of binding | 0.030719 |
| GO:0070647 | biological_process | protein modification by small protein conjugation or removal | 0.030719 |
| GO:0010035 | biological_process | response to inorganic substance | 0.030999 |
| GO:0007346 | biological_process | regulation of mitotic cell cycle | 0.03209 |
| GO:0002694 | biological_process | regulation of leukocyte activation | 0.033895 |
| GO:0050767 | biological_process | regulation of neurogenesis | 0.033895 |
| GO:0019787 | molecular_function | small conjugating protein ligase activity | 0.034887 |
| GO:0010740 | biological_process | positive regulation of protein kinase cascade | 0.034887 |
| GO:0050865 | biological_process | regulation of cell activation | 0.037312 |
| GO:0042802 | molecular_function | identical protein binding | 0.037435 |
| GO:0019900 | molecular_function | kinase binding | 0.037896 |
| GO:0031324 | biological_process | negative regulation of cellular metabolic process | 0.037993 |
| GO:0051345 | biological_process | positive regulation of hydrolase activity | 0.039651 |
| GO:0008544 | biological_process | epidermis development | 0.039651 |
| GO:0051247 | biological_process | positive regulation of protein metabolic process | 0.039945 |
| GO:0009888 | biological_process | tissue development | 0.040154 |
| GO:0048545 | biological_process | response to steroid hormone stimulus | 0.040979 |
| GO:0051960 | biological_process | regulation of nervous system development | 0.041274 |
| GO:0034470 | biological_process | ncRNA processing | 0.043836 |
| GO:0005615 | cellular_component | extracellular space | 0.044576 |
| GO:0007398 | biological_process | ectoderm development | 0.044576 |
| GO:0009314 | biological_process | response to radiation | 0.045194 |
| GO:0060284 | biological_process | regulation of cell development | 0.045958 |
| GO:0009892 | biological_process | negative regulation of metabolic process | 0.047076 |
| GO:0016881 | molecular_function | acid-amino acid ligase activity | 0.048444 |
| GO:0000785 | cellular_component | chromatin | 0.048747 |
| GO:0006897 | biological_process | endocytosis | 0.04983 |
| GO:0010324 | biological_process | membrane invagination | 0.04983 |
